# Metallic nanoparticles in precision medicine for gastrointestinal cancers: Diagnostic and therapeutic advances

**DOI:** 10.1016/j.jpha.2025.101544

**Published:** 2025-12-31

**Authors:** Xu Han, Ding Ding, Milad Ashrafizadeh, Gautam Sethi, Ziwen Wang, Yuting Zhang

**Affiliations:** a Department of Emergency, Shengjing Hospital of China Medical University, Shenyang, 110004, China; b Department of Clinical Nutrition, Shengjing Hospital of China Medical University, Shenyang, 110004, China; c Department of Radiation Oncology and Shandong Provincial Key Laboratory of Radiation Oncology, Shandong Cancer Hospital and Institute, Shandong First Medical University and Shandong Academy of Medical Sciences, Jinan, 250117, China; d Department of Pharmacology, Yong Loo Lin School of Medicine, National University of Singapore, 16 Medical Drive, Singapore, 117600, Singapore; e Department of Radiology, Shengjing Hospital of China Medical University, Shenyang, 110004, China; f Department of Pulmonary and Critical Care Medicine, Shengjing Hospital of China Medical University, Shenyang, 110004, China

**Keywords:** Gastrointestinal cancer, metallic nanoparticles, Drug delivery, Photothermal therapy, Theranostic platforms

## Abstract

Gastrointestinal cancers (GI) rank among the most common and lethal tumors. The traditional therapies face limitations in the early diagnosis, accurate targeting, and treatment effectiveness. Metallic nanoparticles (MNPs) such as Au, Ag, and iron oxide present various promising opportunities owing to their exceptional physicochemical properties including high surface area-to-volume ratios, tunable surface chemistry, and biological interactions. Therefore, understanding the function and application of MNPs in the treatment of specific cancers and advancing cancer diagnosis can provide valuable clinical insights. This review assesses the capabilities of MNPs in GI cancers, focusing on the drug delivery, imaging, photothermal therapy (PTT), and the enhancement of radiotherapy. The primary findings encompass using gold nanoparticles (AuNPs) for the targeted drug delivery, photothermal-induced tumor ablation, and radiosensitization in colorectal, pancreatic, and gastric cancers. AgNPs can not only enhance chemotherapy and photodynamic therapy (PDT) but also induce apoptosis. Iron oxide nanoparticles (IONPs) aid in magnetic drug targeting, enhance magnetic resonance imaging (MRI) contrast, and enable combined chemo-photothermal therapy. Metal-organic frameworks (MOFs) facilitate drug delivery, multimodal imaging, and combination treatments, including photodynamic and immunotherapy, for colorectal and pancreatic cancers. The review emphasizes the recent advancements in MNP-based theranostic platforms, showcasing their ability to enhance the early detection, overcome drug resistance, and boost the therapeutic index. The obstacles such as off-target effects, scalability and chronic toxicity are addressed, along with methods for enhancing MNP design and its clinical use. This review emphasizes the transformative possibilities of MNPs in tackling the worldwide challenge of GI cancers.

## Highlights

• Metallic nanoparticles facilitate precise drug delivery to gastrointestinal cancers.
• Metalic nanoparticles and MOFs enhance diagnostic imaging.
• Gold and silver nanoparticles improve photothermal ablation and radiotherapy.
• Metallic nanoparticles increase combination therapy.
• Metallic nanoparticles and MOFs improve theranostic application.

## Graphical abstract

**Figure:**
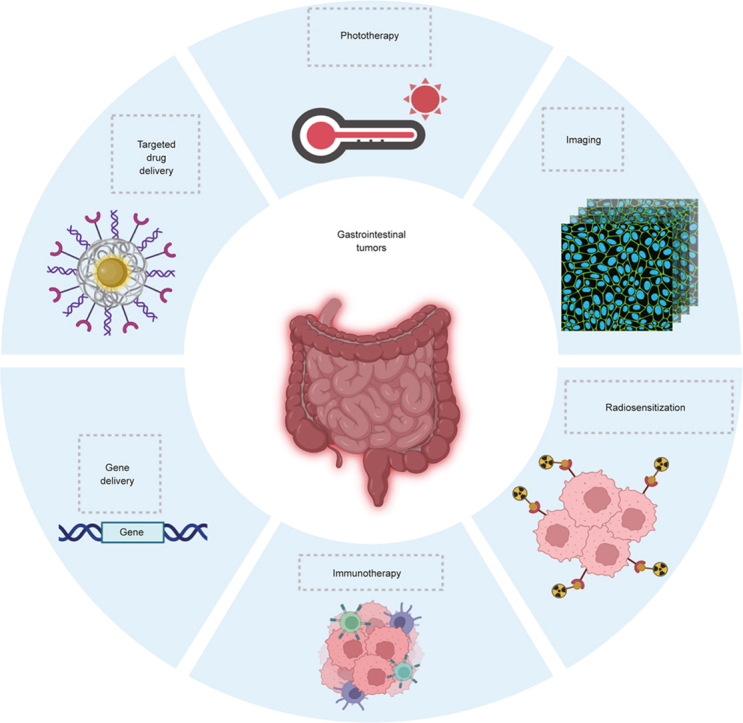

## 1 Introduction

Gastrointestinal cancers (GI) are classified as cancers affecting the esophagus, stomach, liver, pancreas, gallbladder, bile ducts, small intestine, colon, rectum, and anus, presenting a significant global health issue due to rising rates of incidence and mortality. Colorectal cancer (CRC) is the leading form of GI cancer and a major factor in cancer-related deaths worldwide, with risk factors including age, family history, inflammatory bowel disease, and lifestyle factors such as diet, obesity, and tobacco consumption [1,2]. Gastric cancer (GC) may arise from *Helicobacter pylori* infection, dietary factors, and genetic predisposition, and it is one of the most common cancers observed in East Asia. The identification of GC typically takes place at the later and advanced stages, leading to the poor survival rates [[3], [4], [5], [6]]. Hepatocellular carcinoma (HCC) is the most common type of liver cancer and is strongly linked to chronic hepatitis B and C infections, alcohol consumption, and non-alcoholic fatty liver disease (NAFLD), with limited treatment choices for advanced stages [[7], [8], [9]]. Pancreatic cancer (PC), noted for its aggressive nature and late diagnosis, has a poor prognosis, with surgical resection being a treatment option that only a limited number of patients can receive [[10], [11], [12], [13], [14]]. Esophageal cancer (EC), such as adenocarcinoma, is related to the obesity and gastroesophageal reflux disease (ERGE), although it is known that the squamous cell carcinoma is caused by tobacco and alcohol consumption and presents a poor prognosis due to diagnosis in advanced stages [[15], [16], [17], [18]]. Advancements in the early diagnosis, targeted therapies, and immunotherapy, such as immune checkpoint inhibitors, have shown promise in improving outcomes for certain GI tumors; however, the issues with access to care and screening remain challenging. Multifaceted approaches, including precision medicine and biomarker-based accurate diagnosis, are essential for addressing the diversity and complexity of GI tumors, aiming ultimately to reduce their worldwide impact.

The field of cancer treatment has been revolutionized by the emergence of nanotechnology. Nanotechnology enables the precise delivery of drugs while minimizing damage to the healthy tissues. There are various types of nanoparticles, such as liposomes, polymeric nanoparticles, and gold nanoparticles (AuNPs), delivering anti-cancer drugs directed to tumor locations by utilizing enhanced permeability and retention (EPR) effects or through active targeting with ligands binding to the cancer-specific receptors and those that are upregulated in cancer cells. This focused and specific delivery minimizes systemic toxicity and enhances therapeutic effectiveness. Moreover, nanotechnology enables combination therapies, since nanostructures can simultaneously co-deliver drugs, imaging agents, and gene therapies for a multifunctional and combination strategy, resulting in a synergistic effect in cancer treatment. Different examples exist of employing nanoparticles in cancer treatment. Iron oxide nanoparticles (IONPs) can be utilized for magnetic resonance imaging (MRI) and hyperthermia therapy, in which an external magnetic field induces hyperthermia-driven tumor destruction. In an alternative strategy, a hollow mesoporous manganese dioxide-based nanoparticle (M-HMnO_2_@ICG) was developed by incorporating indocyanine green (ICG) through counterion aggregation and additional functionalization with HeLa cell membranes [19]. The nano prodrug irinotecan (ISL) @MIL-101 (a mesoporous Material of Institute Lavoisier-101)-adamantane (ADT) facilitates synergistic cancer treatment by co-delivery of H_2_S and ISL, enhancing tumor targeting, imaging, and therapeutic effectiveness via mitochondrial localization, phototherapy activation, and signaling pathway blockade, showing significant *in vivo* tumor suppression [20]. Moreover, theranostic platforms have been designed to provide both diagnosis and treatment, facilitating real-time observation of drug administration and tumor reaction. The effectiveness of immunotherapy could be enhanced through the use of nanoparticles to activate the immune system or to deliver checkpoint inhibitors. Special attention should also be paid to the development and progress of stimuli-responsive nanoparticles that can react to tumor-specific triggers including pH or enzymes, enhancing the area of precision medicine and minimizing negative effects. Metallic nanoparticles (MNPs) exhibit several properties owing to their superior physicochemical traits, such as a high surface area-to-volume ratio, adjustable surface chemistry, and ability to engage with biological systems, enhancing their use in biomedicine. MNPs have considerable promise in the targeted drug delivery, diagnostic applications, photothermal therapy (PTT), and enhancing radiotherapy. MNPs are frequently used for the delivery of chemotherapeutics. The surface can be functionalized with targeting ligands to precisely deliver drugs, reducing off-target effects. AuNPs possess biocompatibility and can be linked with anti-cancer agents such as doxorubicin. These structures can remarkably enhance the accumulation of drugs via EPR [21]. IONPs are utilized for the magnetic drug targeting, focusing on tumor cells [22]. Gold nanoparticles (AuNPs) of different sizes and shapes can enter and damage the nuclei of cancer cells, disrupting nuclear structure, stress regulation, and nucleolar RNA synthesis, with smaller nanospheres and nanoflowers showing greater toxicity than larger nanospheres. Mild hyperthermia intensified many of these nuclear defects, indicating that AuNPs combined with heat treatment may enhance cancer cell destruction by impairing nuclear organization and ribosome production [23]. Silver nanoparticles (AgNPs) also demonstrate significant plasmonic properties and have been investigated for PTT [24]. MNPs can improve the effectiveness of radiotherapy by boosting the localized radiation dose in tumors. High-Z elements including gold and platinum nanoparticles function as radiosensitizers, absorbing X-rays and emitting secondary electrons damaging cancer cells. AuNPs have been shown to amplify the impact of ionizing radiation in the pre-clinical models [25]. Platinum-based nanoparticles, such as PtNPs, have also been studied for their ability to sensitize to radiation [26]. AuNPs provide high contrast in CT imaging due to a high X-ray absorption coefficient [27].

**Fig. 1:**
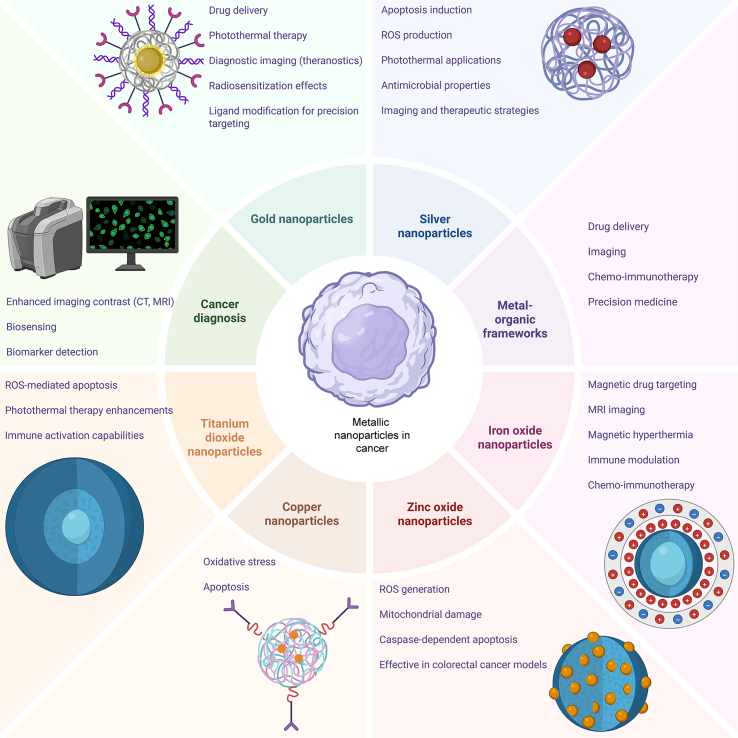
The application of metallic nanoparticles (MNPs) for the treatment of gastrointestinal (GI) tumors. The figure demonstrates the multiple functions of MNPs in cancer therapy, demonstrating their therapeutic and diagnostic applications. Gold nanostructures are widely applied for the drug delivery, photothermal therapy, and theranostics, whereas silver nanostructures show apoptosis stimulation, reactive oxygen species (ROS) production, and antimicrobial characteristics. Iron oxide nanoparticles provide magnetic drug targeting, magnetic resonance imaging (MRI) imaging, and immune regulation, while zinc oxide nanoparticles have been highlighted for the potential for ROS-driven apoptosis. Copper and titanium dioxide nanoparticles participate in cancer therapy through oxidative stress and photothermal enhancements, respectively. Drug delivery and precision medicine can be increased by metal-organic frameworks. CT: computed tomography. Created with biorender.com.

This review focuses on assessing the capabilities of MNPs in diagnosing and treating GI tumors, which pose a significant health challenge due to their prevalence and high mortality rate. This review emphasizes the distinctive physicochemical characteristics of MNPs, such as a high surface area-to-volume ratio, tunable surface chemistry, and the ability to engage with biological systems, rendering them potential candidates in cancer treatment. Moreover, this review illustrates the use of MNPs for the drug delivery, imaging, PTT, and enhancing radiotherapy for GI tumors. Through examining the advancements and obstacles in this area, the review emphasizes the effectiveness of MNPs in improving the early cancer detection, and targeted treatments, enhancing therapeutic outcomes in GI tumors, and finally reducing their global impact. Fig. 1 demonstrates the application of different MNPs in the treatment of GI tumors.

## 2 MNPs in diagnosis and treatment of GI cancers

### 2.1 AuNPs

#### 2.1.1 CRC

AuNPs have been attractive options in CRC therapy due to their unique physicochemical attributes, such as biocompatibility, ease of surface modification, and effective light absorption and scattering. These characteristics have shown potential for uses including targeted drug delivery, PTT, and diagnostic imaging (theranostic applications). Their capacity to be modified with ligands that specifically attach to cancer cells enables them to improve precision medicine, thus reducing damage to the adjacent healthy tissue. The significance of employing AuNPs in cancer treatment results from their ability to surpass the restrictions of conventional therapies by enhancing therapeutic effectiveness, reducing side effects, and enabling real-time observation of treatment responses. The incorporation of AuNPs considerably reduced the survival of HT29 cancerous stem-like cells (CSLCs) and modified radiobiological parameters after irradiation. The γ-H2AX assay showed a significant increase in the persistent DNA double-strand breaks. The relative biological effectiveness (RBE) of AuNPs in conjunction with X-rays was around 1.6 at the 10% cell survival fraction (D10 level). This impact was considerably greater than the impact of X-rays on their own [28]. According to these studies, further studies have evaluated how ligand functionalization on AuNPs influences cellular uptake dynamics in CRC models. A study examined the cellular absorption of hydrophilic mono- and dual-ligand AuNPs in CRC, demonstrating that the rates of uptake are determined by ligand arrangement rather than by charge or chemical composition. The absorption of tiny AuNPs by HCT-116 colon cancer cells is significantly affected by the composition of surface ligands, cell density, and glucose availability. Glutathione-coated AuNPs are absorbed at a significantly greater rate than glucose-coated counterparts, with absorption rising linearly with time and in relation to cell density. The combination of glutathione and glucose significantly diminishes AuNPs internalization, demonstrating that surface ligand composition plays a crucial role in the cellular absorption. Uptake occurs even when conventional endocytic routes are pharmacologically obstructed, suggesting a non-energy-dependent process that is separated from typical endocytosis. These nanoparticles circumvent conventional vesicular trafficking, directly infiltrating the cytoplasm and then aggregating in the endosome-like structures, presumably as a result of intracellular ligand exchange. These findings display a distinct, passive absorption process and emphasize how ligand chemistry and cellular metabolic conditions, such as glucose restriction, influence nanoparticle internalization in cancer cells [29]. Building on the therapeutic potential, another experiment investigated the synergistic effects of AuNP-mediated PTT combined with chemotherapy in three dimensional (3D) CRC spheroid models. It was aimed to assess the synergistic therapeutic potential of integrating PTT facilitated by polyethylene glycol-functionalized gold nanoparticles (AuNP@PEG) with the chemotherapeutic agent doxorubicin (Dox) in three-dimensional CRC spheroid models, including drug-resistant variants (HCT116-DoxR). Spheroids were administered AuNP@PEG and exposed to 532 nm laser irradiation, leading to the increased cell mortality, especially at the spheroid periphery, which gradually advanced into the center. The impact was exacerbated by photothermal activity, observed by rising temperatures and structural degradation over time. Dox penetration was restricted in untreated spheroids but significantly enhanced during AuNP@PEG incubation and irradiation. The time of irradiation was identified as a crucial element in optimizing drug effectiveness and spheroid disintegration. Augmented diffusion and expedited disintegration were highlighted in both sensitive and resistant spheroids, particularly when irradiation was administered following the combination treatment of AuNP@PEG and Dox. These data indicate that this integrated approach may facilitate the efficient tumor targeting while minimizing the necessary dosage of chemotherapeutic drugs [30]. While these results highlight promising avenues, future research provides the necessity of improving AuNP designs to optimize specificity and therapeutic performance. The future studies in CRC treatment should enhance the design of AuNPs to boost targeted uptake and treatment efficacy, while minimizing off-target impact. Altering ligand arrangements on AuNP surfaces to enhance tumor-specific microenvironmental signals such as nutrient availability and cell density could improve the targeted delivery. The combination of AuNP-based PTT with chemotherapy in adaptive, time-controlled protocols shows great potential for tackling multidrug resistance. Additionally, incorporating real-time imaging capabilities into theranostic AuNPs will be crucial for dynamically evaluating therapeutic responses and adjusting treatments on-site. The conversion of these nanotechnologies into clinical use requires comprehensive *in vivo* validation, toxicity evaluations, and the development of scalable and consistent manufacturing methods to ensure safety and effectiveness among various patient populations.

**Fig. 2:**
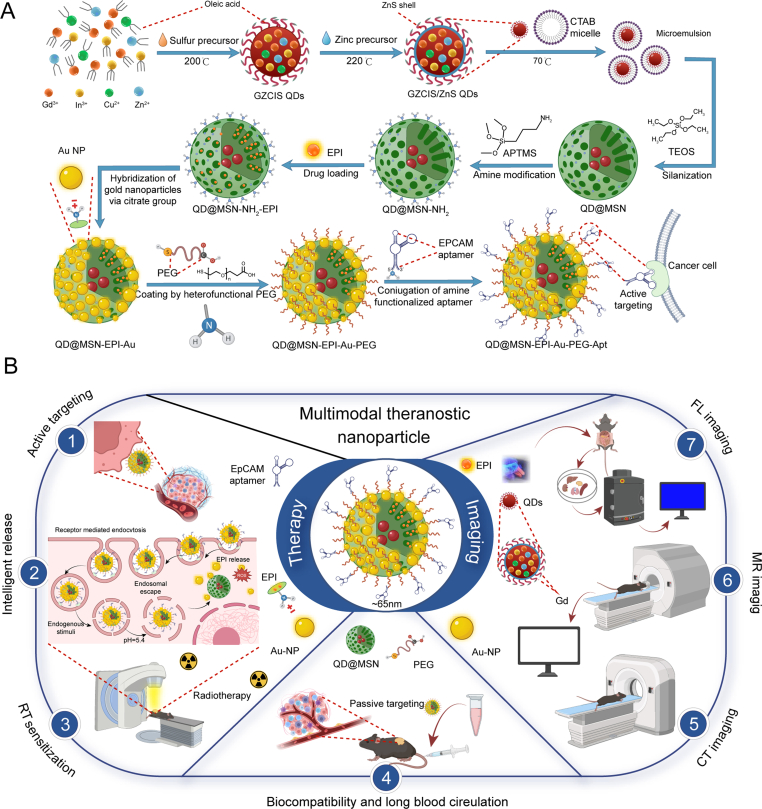
Design and operation of the quantum dots (QDs)@mesoporous silica nanoparticles (MSNs)-epirubicin (EPI)-gold (Au)-polyethylene glycol (PEG)-aptamer (Apt) nanosystem. Depiction showcasing (A) the stepwise creation of the nanocarriers and (B) their diverse multimodal capabilities for theranostic uses. CTAB: n-cetyl trimethyl ammonium bromide; TEOS: tetraethyl orthosilicate; APTMS: (3-aminopropyl) trimethoxysilane; EpCAM: epithelial cell adhesion molecule; RT: radiotherapy; CT: computed tomography; MR: magnetic resonance; FL: fluorescence. Reproduced with permission from Ref. [32].

An experiment examines the efficacy of octaarginine (R8)-modified polyethylene glycol-coated gold nanoparticles (AuNP-PEG-R8) in augmenting the radiosensitivity of LS180 CRC cells during megavoltage irradiation *in vitro*, as well as the processes involved. The synthesized AuNPs were studied using transmission electron microscopy (TEM), dynamic light scattering (DLS), UV–visible spectrophotometry, and X-ray photoelectron spectroscopy. The fundamental AuNPs were measured at 6.3 ± 1.1 nm, whereas functionalization elevated the hydrodynamic diameters to 19.7 ± 2.8 nm for AuNP-PEG and 27.8 ± 1.8 nm for AuNP-PEG-R8, with corresponding red-shifts in plasmon resonance peaks at 515 nm and 525 nm, respectively. Inductively coupled plasma mass spectrometry demonstrated a significantly increased cellular absorption of AuNP-PEG-R8 relative to AuNP-PEG (*P* < 0.001) after 1 h. Cytotoxicity evaluations revealed partial toxicity at doses under 400 nM. Clonogenic experiments demonstrated that AuNP-PEG-R8 markedly improved radiation-induced cytotoxicity, yielding a sensitizer enhancement ratio of 1.59 relative to radiation alone. Mechanistic investigations utilizing flow cytometry demonstrated that the combination treatment resulted in a significant G2/M phase cell cycle arrest, 10% greater than AuNP-PEG alone and a substantial increase in apoptosis (*P* < 0.001). Furthermore, increased levels of reactive oxygen species (ROS) and disturbance of mitochondrial membrane potential (MMP) were noted after the combined AuNP-PEG-R8 and radiation therapy (*P* < 0.001), indicating oxidative stress as a principal catalyst of radiosensitization. AuNP-PEG-R8 nanoparticles were effectively absorbed by cancer cells, demonstrating partial cytotoxicity while significantly augmenting radiation effects via ROS production, cell cycle arrest, and apoptosis induction. [31]. Complementing these efforts, a different study engineered multifunctional hybrid nanocarriers that combine diagnostic and therapeutic functions to enhance CRC targeting and treatment. Multimodal nanoparticles that integrate quantum dots (QDs), mesoporous silica nanoparticles (MSNs), and AuNPs constitute a potential approach for targeted cancer treatment and diagnostic imaging. This method involves initially coating magnetic GZCIS/ZnS QDs with mesoporous silica, thereafter loading the chemotherapeutic drug epirubicin into the silica pores. The system is then encapsulated with AuNPs, PEGylated for biocompatibility, and functionalized with epithelial cell adhesion molecule (EpCAM) aptamers to facilitate precise targeting of CRC cells. The resultant nanocarriers, known as QD@MSN-EPI-Au-PEG-Apt, possess an average particle size of around 65 nm and are subjected to comprehensive analysis post-synthesis. *In vitro* experiments demonstrate that these targeted nanoparticles display preferential cytotoxicity towards HT-29 cells, while exerting negligible effects on non-cancerous CHO cells. Furthermore, when used in conjunction with irradiation, the nanocarriers significantly increase cell mortality in HT-29 cells. *In vivo* tests further validate the targeted delivery capabilities of the nanocarriers, exhibiting enhanced anti-tumor activity and less systemic toxicity during chemo-radiotherapy. The technology also enables real-time imaging in a CRC mouse model. This hybrid nanocarrier technology presents an effective strategy for enhancing theranostics in CRC (Fig. 2) [32]. Therefore, future research should focus on improving nanoparticle surface engineering to boost selective uptake and reduce off-target interactions, especially by fine-tuning peptide and aptamer arrangements for optimal receptor binding and cellular uptake. Broadening combinatorial strategies such as combining AuNP-driven PTT and photodynamic therapy (PDT) effects with conventional chemo-radiotherapeutic treatments, may enhance tumor destruction further. Besides, adjusting nanoparticle size, shape, and ligand density may provide a better understanding of biodistribution patterns and dynamics of intracellular trafficking. Sophisticated imaging and mechanistic investigations are necessary to elucidate nanoparticle-induced modulation of oxidative stress pathways, DNA damage reactions, and apoptotic signaling pathways. Integrating components such as redox-sensitive or enzymatically degradable linkers might enhance on-demand drug release, offering a tailored foundation for targeted treatments in CRC theranostics.

#### 2.1.2 PC

The AuNPs show potential in treating PC, as they can interact with tumor cells to enhance targeted delivery and minimize side effects. AuNPs can induce hyperthermia in PC treatment via PTT, offering both therapeutic and imaging capabilities. The optical properties of AuNPs can enhance the accuracy of diagnosis. A facile, quick, and eco-friendly approach was devised for the production of AuNPs utilizing an aqueous extract of *Panax notoginseng* leaves, therefore obviating the necessity for hazardous reducing agents and aggressive chemicals. The produced AuNPs were meticulously analyzed to verify their dimensions, morphology, crystalline structure, and the existence of functional surface groups. The nanoparticles exhibited stability, with an average size of roughly 128 nm and diverse morphologies, including spherical and polygonal shapes. Their biological activity was evaluated against human PC cells (PANC-1), revealing significant growth suppression, especially at doses of 15 and 20 μg/mL. The nanoparticles were demonstrated to generate intracellular ROS, resulting in oxidative stress and initiating nuclear fragmentation and apoptosis. Increased activity of essential apoptotic enzymes was observed, and protein expression analysis demonstrated downregulation of anti-apoptotic markers and increase of pro-apoptotic markers, signifying activation of the intrinsic apoptotic pathway. The AuNPs derived from *Panax notoginseng* demonstrated significant anticancer properties via ROS-mediated apoptosis, indicating a potential and sustainable strategy for future biomedical applications [33]. To further enhance therapeutic efficacy, subsequent research focused on designing multifunctional nanocarriers combining chemotherapy with photothermal effects, offering a synergistic approach for PC treatment. A multifunctional drug delivery system was developed by encapsulating paclitaxel in poly(lactic-co-glycolic acid) (PLGA) microspheres, covering them with polydopamine, and attaching AuNPs to their surfaces to attain a synergistic chemo-photothermal therapeutic effect for PC treatment. The microspheres had a spherical shape with dimensions between 1 and 5 μm and effectively encapsulated paclitaxel in an amorphous form, hence improving its stability and bioavailability while facilitating regulated and sustained drug release without an initial burst effect. The polydopamine coating facilitated strong adherence of AuNPs, which preserved their photothermal conversion properties during near-infrared irradiation, efficiently transforming light into heat and producing localized hyperthermia. The creation of heat, along with the chemotherapeutic effects of paclitaxel, resulted in increased cytotoxicity towards PC cells by inducing apoptosis, elevating ROS, compromising cellular membranes, and diminishing long-term cell growth. The system exhibited little cytotoxicity in the absence of irradiation, suggesting the carrier's biocompatibility, whilst combination therapy revealed significantly enhanced effectiveness relative to singular therapies. The downregulation of essential antioxidant enzymes during therapy signifies increased oxidative stress, which further facilitates cancer cell death. This integrated delivery system provides a focused, stable, and efficient platform for the treatment of locally advanced PC by synergistic chemo-photothermal therapy, with prospects for the clinical translation and future use in metastatic contexts [34]. Pushing the boundaries of precision medicine, another innovative study developed a responsive phototheranostic system tailored to the unique tumor microenvironment (TME) of PC, aiming to integrate advanced imaging with potent therapeutic action.

**Fig. 3:**
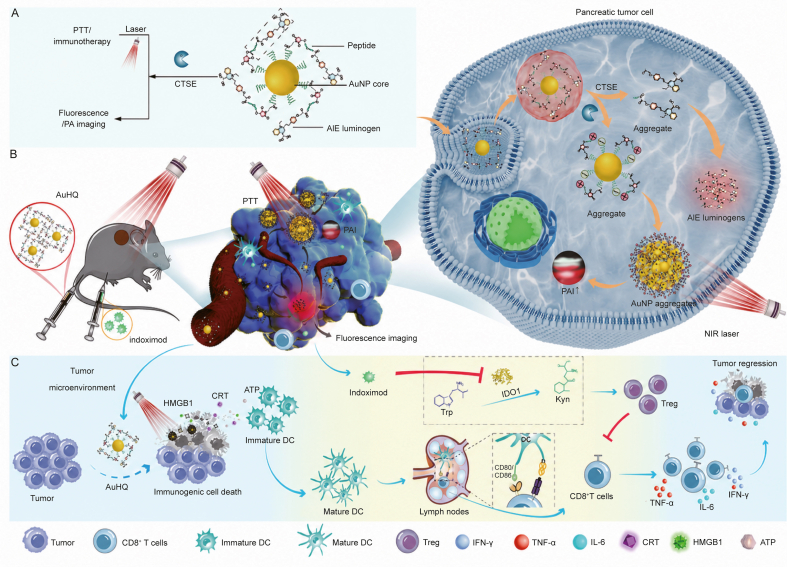
The design and application of nanoparticles in pancreatic cancer therapy. (A) The formulation of gold nanoparticle-based hybrid with aggregation-induced emission (AIE) luminogen and peptide (AuHQ) was developed. (B, C) A diagram illustrates the cathepsin E (CTSE)-activated AuHQ for fluorescence-photoacoustic imaging (PAI) (B) and the combined photothermal immunotherapy approach for pancreatic cancer (C). AuNP: gold nanoparticle; PTT: photothermal therapyNIR: near-infrared; DC: dendritic cell; CRT: calreticulin; HMGB1: high mobility group box 1; ATP: adenosine triphosphate; IDO1: indoleamine 2,3-dioxygenase 1; Trp: tryptophan; Kyn: kynurenine; Treg: regulatory T cell; IFN-γ: interferon gamma; TNF-α: tumor necrosis factor alpha; IL-6: interleukin 6; CD8^+^ T cells: cytotoxic T lymphocytes. Reproduced with permission from Ref. [35].

Efficient imaging and precise therapy are crucial for controlling the advancement of PC. Nonetheless, current theranostic approaches frequently exhibit insufficient tumor selectivity and depend significantly on invasive surgical techniques. A new phototheranostic agent, designated AuHQ, has been meticulously developed to address these issues. This method combines aggregation-induced emission (AIE) luminogens with AuNPs to provide dual-modality fluorescence and photoacoustic imaging, together with targeted PTT. AuHQ is engineered to precisely target the pancreatic TME. The sequence includes a cleavable peptide (Ala-Gly-Phe-Ser-Leu-Pro-Ala-Gly-Cys, or AGFSLPAGC) that is specifically identified and cleaved by cathepsin E (CTSE), an enzyme that is overexpressed in PCs. Following enzyme activation, two self-assembly processes commence: the aggregation of AuNPs and the clustering of AIE luminogens. The enzyme-mediated aggregation of AuNPs significantly improves PTT under near-infrared (NIR) laser irradiation, resulting in immunogenic cell death (ICD) and enhanced photoacoustic imaging efficacy. The simultaneous hydrophobic aggregation of AIE luminogens produces intense fluorescence, allowing precise and real-time imaging of PC. To enhance treatment effects, the AuHQ system may be given in conjunction with an indoleamine 2,3-dioxygenase 1 (IDO1) inhibitor. This combination targets the immunosuppressive characteristics of the TME, hence enhancing the anticancer immune response and optimizing the synergistic benefits of the therapy (Fig. 3) [35]. Therefore, it can be concluded that there is an evolving concept regarding the application of AuNP-based therapies and it provides the importance for the combination of targeted delivery, precise imaging, and combinatorial treatment strategies for more effective PC management.

#### 2.1.3 HCC (liver cancer)

AuNPs can be used in HCC therapy via various mechanisms, including targeted drug delivery, PTT, and radiation enhancement. The functionalized AuNPs containing anti-cancer drugs can be further enhanced with antibodies or peptides to improve the specific targeting of cancer cells and reduce off-target effects. In PTT, AuNPs can absorb NIR light, generating localized heat ablating tumor cells. Moreover, their elevated atomic number boosts radiotherapy by intensifying ionizing radiation uptake, enhancing tumor cell destruction, without affecting healthy tissue. Their tunable surface chemistry and compatibility with biological systems make them promising for the versatile treatment of HCC. There has been an increasing focus on the production and characterisation of AuNPs from plant-derived extracts, especially regarding their prospective anticancer properties. *Marsdenia tenacissima* (MT), a traditional Chinese medicinal plant, has been utilized traditionally to address conditions such as tracheitis, asthma, and rheumatism. A study adjusted reaction parameters to regulate the size of produced nanoparticles, which were analyzed using several analytical methods. The antitumor efficacy of MT-AuNPs was evaluated against hepatic cancer cells. The results demonstrated a notable cytotoxic impact at a concentration of 59.62 ± 4.37 μg following 24 h of treatment. The findings demonstrated that the nanoparticles triggered apoptosis by elevating oxidative stress, impairing mitochondrial activity, and suppressing migration. Moreover, the expression levels of proteins associated with apoptosis exhibited an elevation in pro-apoptotic markers and a reduction in anti-apoptotic markers, therefore validating the apoptotic action of the MT-AuNPs [36].

Liver cancer is a malignant disease that presents a considerable risk to the human health and life. The recent efforts have been directed towards the treatment of this disease. Drug delivery systems can enhance the targeted administration of drugs to liver tissue, augment their bioavailability, and mitigate adverse effects, particularly when these systems are tailored by structural modifications or the development of targeted molecules. Docetaxel-loaded gold-hydroxyapatite nanoparticles (Dtxl-GHANPs) were effectively synthesized and assessed, exhibiting favorable physicochemical, *in vitro*, and *in vivo* characteristics. X-ray diffraction (XRD) and TEM investigations validated the nanorod shape and crystalline structure of GHANPs, revealing an average particle size of around 80 nm and a polydispersity index (PDI) of 0.166, signifying homogeneity. DLS examination indicated an average particle size of 125.7 nm and a negative zeta potential of −13.19 mV, implying stability and the presence of oleic acid coating. The drug loading efficiency was elevated at 95.1%, with a loading capacity of 4.10% (*w/w*), and *in vitro* release experiments demonstrated a sustained, triphasic release of Dtxl for a duration of 100 h. Dtxl-GHANPs were non-toxic to normal HPL cells while displaying considerable, dose-dependent cytotoxicity towards HepG2 liver cancer cells, with an IC_50_ of 1.26 μg/mL. Microscopic examinations and acridine orange/ethidium bromide (AO/EB) and hoechst (HO) stains validated apoptosis-related morphological alterations in treated HepG2 cells, encompassing chromatin condensation, cellular shrinkage, and the production of apoptotic bodies. Dtxl-GHANPs also induced alteration of mitochondrial membrane potential, corroborating the apoptotic mechanism. *In vivo* histopathology examination demonstrated that Dtxl-GHANPs reduced liver cancer-related cellular abnormalities while exhibiting low systemic toxicity, since normal spleen, kidney, and lung tissues had no pathological alterations. Hence, Dtxl-GHANPs serve as a biocompatible, efficient, and targeted nanocarrier technology for the controlled drug administration, offering potential therapeutic advantages in cancer therapy, therefore necessitating additional *in vivo* effectiveness and biodistribution assessments for clinical use [37]. Further advancements introduced multifunctional nanoprobes that integrate precise imaging with synergistic phototherapy, offering a more comprehensive approach to HCC treatment.

AuNPs-based nanomaterials are widely utilized in biomedical applications, such as drug transport, imaging, and treatment, owing to their unique physicochemical properties and superior biocompatibility. A research involved the synthesis of ultra-small AuNPs, which were then coordinated with gadolinium ions to develop spherical self-assembled structures. The nanostructures were functionalized with matrix metalloproteinase-2 (MMP-2) and incorporated with the photosensitizer IR820 to provide combined PTT and PDT for liver cancer treatment. The constructed nanoprobes exhibited *in vivo* metabolization due to their acid-responsive degradation characteristics, allowing dual-mode real-time imaging. The integration of surface-modified MMP-2 endowed the nanoprobes with enhanced tumor-targeting efficacy. In animal models, laser-activated therapy utilizing these nanoprobes significantly inhibited tumor development by augmenting the synergistic effects of PTT and PDT. The result highlights the potential applicability of these multifunctional nanoprobes in liver cancer treatment, facilitated by real-time dual-mode imaging [38]. Complementing these multifunctional strategies, AuNPs were effectively manufactured utilizing *Dendrobium officinale* (DO) extract via an eco-friendly process, with the objective of establishing an innovative and biocompatible treatment approach for liver cancer. The resultant DO-coated AuNPs (Do-AuNPs) exhibited a spherical morphology with an average diameter of around 30 nm and a 13% coating of DO extract. The particles demonstrated improved stability and surface roughness, presumably attributable to the intricate and changeable phytochemical content of the DO extract. *In vitro*, a notable dose-dependent suppression of liver cancer cell survival was reported, although normal liver cells were mostly unaffected, demonstrating excellent selectivity and biocompatibility. *In vivo*, employing a subcutaneous liver tumor model in mice demonstrated that intraperitoneal administration of Do-AuNPs resulted in significant tumor growth inhibition without apparent side effects or organ toxicity, as confirmed by histological analysis and biochemical evaluation of liver and kidney function. Analysis of tumor tissue indicated increased cell mortality and decreased cell proliferation post-treatment, while higher spleen and thymus indices implied potential engagement of immune mechanisms. The Do-AuNPs synthesized by a traditional medicine-based green methodology shown significant anti-cancer efficacy in both cellular and animal models, along with outstanding biosafety, hence endorsing its advancement as a unique therapeutic alternative for liver cancer [39]. Parallel to these therapeutic innovations, research has also focused on engineering AuNPs as highly effective imaging agents to improve diagnostic accuracy for HCC. A unique approach has been devised for the synthesis of lactobionic acid (LA)-modified dendrimer-entrapped gold nanoparticles (LA-Au DENPs) intended for targeted computed tomography (CT) imaging of human HCC, applicable in both *in vitro* and *in vivo* settings. This method utilized generation 5 amine-terminated poly(amidoamine) dendrimers, pre-functionalized with fluorescein isothiocyanate and poly(ethylene glycol)-linked LA, as templates for the development of AuNPs. The subsequent acetylation of the remaining terminal amines of the dendrimer resulted in the synthesis of LA-Au DENPs. The resultant nanoparticles were characterized employing diverse approaches. The multifunctional Au DENPs, with a gold core size of around 2.7 nm, have significant durability across various pH levels (5–8), temperatures (4–50 °C), and aqueous conditions. These nanoparticles exhibited negligible cytotoxicity to the normal cells while displaying selective cytotoxic effects on targeted hepatocarcinoma cells within a defined dose range. LA-Au DENPs are effectively internalized by a hepatocarcinoma cell line through an active receptor-mediated uptake pathway, attributed to the overexpression of asialoglycoprotein receptors. Furthermore, LA-Au DENPs serve efficiently as nanoprobes for targeted CT imaging of hepatocarcinoma cells, both *in vitro* and inside a xenograft tumor model. LA-Au DENPs exhibit superior X-ray attenuation compared to traditional iodine-based CT contrast agents, indicating substantial promise as targeted imaging agents for diagnosing human HCC [40].

In pursuit of maximizing therapeutic precision, another approach paired ultrasmall AuNPs with potent cytotoxic agents to design nanomedicines with function better than conventional treatments. A considerable unmet clinical need exists for the novel therapeutic alternatives. A study assessed MTC-100038, a systemic nanomedicine comprising 2 nm ultrasmall gold core nanoparticles (MidaCore) linked with the powerful maytansine analogue DM1, for its efficacy in treating HCC. The nanoparticle formulation improved the tolerance of DM1, allowing for a dosage increase of almost threefold compared to the unencapsulated drug. Furthermore, at comparable dosages, MTC-100038 accomplished about double the intratumoral delivery of DM1 (*P* = 0.039) in comparison to free DM1. MTC-100038 demonstrated significant anticancer activity in several murine xenograft models of HCC, achieving a tumor growth index of around 102% (*P* < 0.0001), surpassing the performance of both free DM1 and sorafenib, the established standard of therapy. *In vitro* investigations demonstrated the nanomolar efficacy of MTC-100038 across many primary patient-derived HCC cell lines and multiple additional cancer cell types [41]. Another work examines the anti-cancer efficacy of AuNPs coupled with the therapeutic dye thionine (TN), resulting in two complexes, including gold-thionine complex 1 (GTN1) and gold-thionine complex 2 (GTN2), synthesized under varying circumstances. GTN1 is generated when TN functions as a stabilizer during the reduction of gold ions, whereas GTN2 is formed by the electrostatic adsorption of TN onto the surfaces of GNPs. Thorough evaluation validated the structural and optical attributes of the nanoparticles. GTN1 demonstrated robust DNA binding and elicited considerable cytotoxicity in HepG2 liver cancer cells via ROS production, resulting in nuclear fragmentation and death. GTN1 provided more anti-proliferative efficacy than GTN2, highlighting its potential in cancer nanotherapy applications [42]. While these studies demonstrate impressive advancements, they also highlight ongoing challenges in translating AuNP-based strategies to the clinical practice. Even with the encouraging therapeutic and diagnostic possibilities of AuNP-based platforms for HCC, numerous challenges persist. Attaining accurate tumor targeting while reducing off-target accumulation remains a challenge, as evidenced by biodistribution studies indicating significant uptake in organs such as liver and kidney. The intricate nature of TME, including acidic pH, enzymatic action, and immune suppression, requires engineered nanoparticles to provide stability, responsive delivery, and efficient penetration. While *in vitro* and *in vivo* findings revealed significant cytotoxic effects, ROS production, and apoptosis induction, the long-term biosafety and toxicity profiles warrant further investigation, especially concerning repeated administration and clinical application. Additionally, differences in nanoparticle size, shape, and surface chemistry can influence consistency and reproducibility in therapy. The scalability of synthesis techniques, regulatory challenges, and the incorporation of multifunctional features (merging imaging with treatment) present further obstacles to clinical application.

#### 2.1.4 GC

Therapeutic strategies for GC still present significant hurdles, while nanomedicines offer incredible potential. The advancement of multifunctional nanoparticles, particularly those showing improved targeting capabilities and anticancer effectiveness, is crucial for improving therapeutic outcomes. AS1411-conjugated AuNPs provide a highly efficient, targeted methodology for chemotherapy in GC cells, especially human gastric adenocarcinoma cell line (AGS) cells, utilizing aptamer-mediated transport, pH-sensitive drug release, and laser-activated response. TEM and UV–vis analyses validated the successful production of nanoparticles, whilst flow cytometry and confocal imaging demonstrated about two-fold enhanced binding of AS1411-based nanoparticles to AGS cells relative to random DNA-based controls, suggesting a nucleolin-mediated targeting mechanism. Cellular uptake investigations demonstrated a preferential accumulation of Dox in AGS cells compared to L929 cells, which exhibit less nucleolin expression, hence confirming the selective cytotoxicity. Nanoparticles were efficiently ingested by endocytosis and localized into lysosomes, where the acidic pH promoted the release of Dox. Laser irradiation at 808 nm significantly increased intracellular Dox release, particularly at elevated power levels, hence enhancing chemotherapeutic effectiveness. In comparison to free Dox and PolyT-based nanoparticles, AS1411-based nanoparticles demonstrated significantly enhanced cytotoxicity against AGS cells, especially under laser irradiation, while ensuring stability and little release at physiological pH, underscoring their safety. The nanoparticles, in the absence of Dox, induced minimal damage, demonstrating the system's biocompatibility. These findings indicate that AS1411-conjugated AuNPs, with laser-activated drug release and targeted delivery, may provide a novel, safer, and more efficacious therapeutic approach for gastric cancer therapy [43]. It was recently shown that the transcription factor sry-related hmg-box protein 13 (SOX13) is crucial for ferroptosis resistance in GC by upregulating SCAF1, thereby improving mitochondrial respiration and the formation of electron transport chain supercomplexes. Targeting SOX13, especially by using zanamivir, successfully reinstated sensitivity to ferroptosis, counteracted cisplatin resistance, and enhanced immunotherapy effectiveness both *in vitro* and *in vivo* [44]. GC is the fourth most prevalent cancer globally and the second main cause of cancer mortality. Notwithstanding the availability of interventions such as chemotherapy, surgery, or their combination, numerous patients encounter recurrence, drug resistance, and poor prognoses. Consequently, the pursuit of efficacious anticancer drugs for GC remains an essential objective. Nanobiotechnology has emerged as a possible option in cancer therapy, notably through the eco-friendly and cost-effective green production of metal nanoparticles utilizing plant extracts, which presents benefits over conventional chemical approaches. In this context, AuNPs were designed via an ethanol extract of *Cirsium japonicum* (CJ), resulting in the development of CJ-mediated AuNPs (CJ-AuNPs). Their stability was evaluated over a three-month duration under *in vitro* conditions. CJ-AuNPs preferentially promoted apoptosis in AGS cells while preserving normal cells. Subsequent *in vitro* investigations demonstrated that CJ-AuNPs induce oxidative stress and activate ferroptosis, an iron-dependent mode of cellular demise. This method entails the production of mitochondrial ROS, elevated Fe^2+^ concentrations, intensified lipid peroxidation, and mitochondrial impairment, via the disruption of the glutathione peroxidase-4 (GPX4) antioxidant system. Furthermore, an *in vivo* investigation utilizing a xenograft mouse model implanted with AGS cells demonstrated that the administration of CJ-AuNPs at dosages of 2.5, 5, and 10 mg/kg over 16 days resulted in a dose-dependent decrease in tumor development, without indications of systemic toxicity [45].

Alongside ferroptosis-focused approaches, another work has emphasized on human epidermal growth factor receptor 2 (HER2)-targeted AuNP systems, aiming to enhance specificity and therapeutic potency in GC. A persistent issue is the absence of efficacious HER2-targeted treatments for trastuzumab (Tmab)-resistant GC. AuNPs have emerged as effective drug carriers owing to their extensive surface area, which enhances the binding of agents including antibodies. HER2-targeted thionine-conjugated gold nanoparticles (T-AuNPs) were developed and assessed their therapeutic effectiveness and cytotoxic processes in HER2-positive trastuzumab-resistant (MKN7) and trastuzumab-sensitive (NCI-N87) GC cell lines. *In vitro* investigations revealed that T-AuNPs had more cytotoxic effects than control treatments in both MKN7 and NCI-N87 cells, while Tmab alone had no effect on MKN7 cells. The harmful effects of T-AuNPs were predominantly caused by autophagy, triggered by the cellular uptake of the nanoparticles. *In vivo* investigations also validated that T-AuNPs elicited substantial anticancer effects in subcutaneous tumor models of both NCI-N87 and MKN7. The data indicate that HER2-targeted AuNPs conjugated with Tmab provide a viable treatment strategy for addressing Tmab resistance in gastric cancer [46]. To complement therapeutic strategies, diagnostic innovation has centered on employing stellate AuNPs for sensitive miRNA detection, providing precise molecular profiling in GC. A variety of nanoprobes have been investigated for the surveillance of biological systems through the detection and visualization of intracellular biomarkers such as miRNAs. A direct one-step method was developed to design stellate AuNPs conjugated with miR-responsive molecular beacons (SGNP-MBs), wherein acidic conditions promoted effective and quick attachment of MBs to the SGNP surface. Compared to the conventional AuNP-based molecular beacons, SGNPs exhibited a 4.5-fold enhancement in beacon loading and a 6.4-fold increase in cellular absorption. The SGNP-MBs were well internalized by human GC cell lines, allowing accurate detection and imaging of intracellular miRs in a sequence-specific manner. Furthermore, they facilitated the observation of diverse miR-10b expression levelshigh, moderate, and low, across three distinct cell lines. The observations indicate that SGNP-MBs may serve as potent and effective instruments for intracellular miR analysis in live cells [47].

Ramucirumab (Ab), the first monoclonal antibody approved by the US Food and Drug Administration (FDA) for advanced GC, was linked with AuNPs to improve cellular uptake and therapeutic efficacy. AuNP-based drug delivery systems, particularly gold nanorod (AuNR) and gold nanosphere (AuSP) functionalized with PEG, the chemotherapeutic agent Dox, and the monoclonal antibody Ab, were engineered and described for the targeted treatment of GC. TEM and spectroscopy validated effective synthesis, revealing thicker nanoparticle capsules upon the addition of Ab. PEG modification induced a red shift in the absorbance spectra, and the conjugation of PEG and antibody was confirmed using Fourier transform infrared spectroscopy (FTIR) and dynamic light scattering. *In vitro* cellular uptake assays with GC cells (SNU-5 and MKN-45) demonstrated increased fluorescence intensity and accumulation in AuNR-PEG-Ab-treated cells relative to those treated with Ab alone, with significant internalization observed within 4 h. *In vivo* imaging of tumor-bearing mice demonstrated enhanced tumor formation and extended retention of AuNR-PEG-Ab compared to Ab and AuSP-PEG-Ab, even at equal antibody dosages. Ab alone had no impact on GC and normal gastric cells, but AuNR-PEG-Ab and AuSP-PEG-Ab significantly reduced GC cell survival, with the rod-shaped nanocarriers exhibiting more pronounced cytotoxic effects. The cytotoxicity was not attributable to the core materials but was associated with the biological interaction between AuNPs and antibodies. Proteomic and transcriptome studies indicated that AuNR-PEG-Ab elicited extensive immunological and apoptotic responses, incuding pathways including “phagosome”, “lysosome”, and “fragment crystallizable gamma receptor (FcγR)-mediated phagocytosis”. The high-affinity Fcγ receptor CD64 was increased in GC cells treated with AuNR-PEG-Ab, but not in normal gastric epithelial cells, hence corroborating the reported selective toxicity. ROS production was also increased in AuNR-PEG-Ab-treated cells, facilitating apoptosis. *In vivo*, AuNR-PEG-Ab significantly suppressed tumor development while preserving normal organ integrity [48]. Expanding beyond antibody-linked nanodrugs, enzymatic delivery systems using gold nanocomposites were followed to specifically target and suppress GC growth. GC ranks as the second foremost cause of cancer-related death globally, with cancer stem cells (CSCs) identified by CD44 significantly influencing disease advancement. Recombinant methioninase (rMETase) has been investigated as a chemotherapeutic treatment for GC, and polymer-based nanoparticle drug delivery methods, particularly those functionalized with hyaluronic acid (HA) a natural ligand for CD44 provide improved biocompatibility and water solubility. This study utilized HA-modified G5 PAMAM-Au nanoparticles for the delivery of rMETase (HA-G5 PAMAM-Au-METase). HA-G5 PAMAM-Au-mediated rMETase administration significantly inhibited GC cell proliferation and tumor sphere development by enhancing METase activity, therefore reducing extracellular methionine levels and boosting Cyt C and ROS levels. The mitochondrial dysfunction resulted in decreased proliferation of CD44-positive GC cells. In treated animals, the number of CD44-positive GC cells was decreased, leading to substantial tumor growth inhibition. HA-G5 PAMAM-Au-METase demonstrated significant anticancer efficacy by specifically targeting and impairing mitochondrial activity in CD44-positive gastric cancer stem cells [49].

AuNPs are widely used in bio-imaging, medical therapy, enzyme assays, and environmental applications because of their biocompatibility, large surface area, and high dispersion properties. Green synthesis of AuNPs using plant extracts is preferred over conventional methods because it reduces toxic by-products and produces highly bioavailable particles with potential applications in cancer treatment, including colon cancer [50]. While there have been major advancements in AuNP-based nanomedicines for GC, numerous important challenges persist. Attaining accurate tumor targeting while reducing off-target accumulation remains a constant challenge, as differing receptor expression (nucleolin, HER2) and tumor heterogeneity may restrict uptake and therapeutic uniformity (this is also of high importance, when the clinical application of these drugs is followed). Moreover, achieving stability and regulated drug release in the complex environment of tumors, which is often acidic, rich in enzymes, and immunosuppressive, requires precisely engineered nanoparticle designs. Although numerous studies show strong *in vitro* and *in vivo* effectiveness, the transition of these findings into clinical success is suppressed by issues related to the scalability, possible long-term toxicity, and immune system clearance. Imaging and biosensing systems also encounter difficulties in maintaining high specificity and signal fidelity within complex biological fluids. Ultimately, resistance mechanisms such as ferroptosis evasion or modulation of autophagy can diminish therapeutic outcomes, highlighting the necessity for combinatorial or multi-faceted approaches to maintain effectiveness among various patient groups.

#### 2.1.5 EC

AuNPs have emerged as a valuable resource in EC treatment because of their distinctive physicochemical characteristics, which include biocompatibility, the ability to functionalize surfaces. In drug delivery, AuNPs can be infused with chemotherapy drugs (cisplatin, doxorubicin) and directed to EC cells via ligands such as antibodies or peptides, increasing drug concentration while reducing systemic toxicity. In PTT, AuNPs (especially AuNR or nanoshells) capture NIR light, transforming it into heat to targetedly destroy tumor cells while sparing adjacent healthy tissue from significant harm. Moreover, AuNPs function as radiosensitizers by amplifying the impacts of radiotherapy via enhanced X-ray absorption, thereby boosting the effectiveness of tumor cell destruction. Their optical characteristics also aid imaging methods such as photoacoustic imaging and surface-enhanced Raman spectroscopy (SERS) for accurate tumor identification and therapy observation. In addition, AuNPs can be designed for combination therapies, merging chemotherapy, PTT, and immunotherapy to address treatment resistance in aggressive EC. These multifunctional applications position AuNPs as a flexible platform for enhancing the diagnosis and treatment of EC. Radiotherapy and chemotherapy encounter constraints owing to the diminished therapeutic effectiveness of low-dose radiation and the nonspecific biodistribution of pharmaceuticals. An acid-responsive aggregated nanosystem (AuNPs-D-P-DA) loaded with Dox is designed to tackle these problems, allowing improved radiosensitization and synergistic chemoradiotherapy. In the acidic microenvironment typical of EC, tiny AuNPs-D-P-DA convert into massive aggregates of AuNP within tumor tissues. This aggregation diminishes the probability of AuNPs re-entering circulation, resulting in enhanced accumulation and extended retention at the tumor site. The increased concentration and size of intratumoral AuNPs significantly enhance their radiosensitization impact, while Dox is preferentially transported and released into tumor cells in reaction to acidic circumstances, promoting a synergistic chemo-radiotherapeutic result. These acid-responsive AuNPs exacerbate radiation-induced DNA damage, facilitate cell death, cause cell cycle arrest, and reduce colony formation *in vitro*. *In vivo* studies indicate superior anti-tumor activity relative to non-responsive controls. The integration of acid-responsive AuNPs with Dox enhances therapeutic efficacy via a synergistic mechanism. Post-treatment in xenograft animals indicates a reconfiguration of fatty acid metabolism, highlighting prospective targets for forthcoming radiosensitization techniques [51]. Although AuNP-based approaches present encouraging progress in treating EC, numerous challenges remain. Gaining exact control over nanoparticle clustering and drug release in the acidic TME is still complicated, as differences in tumor pH and variability may influence treatment reliability along with the presence of tumor heterogeneity. A major challenge is achieving thorough tissue penetration and uniform distribution of AuNPs, particularly in large or poorly vascularized tumors. While *in vitro* and *in vivo* findings demonstrate strong radiosensitization and chemotherapeutic effects, thorough assessment of long-term biosafety, possible immunogenicity, and nanoparticle clearance is necessary before clinical use. Moreover, increasing the reproducible production of multifunctional AuNPs while ensuring physicochemical stability and functional effectiveness is technically challenging. Furthermore, integrating AuNP-based systems into current clinical workflows aiming at accurate imaging and dosing optimization presents logistical and regulatory issues that require resolution to fully utilize their therapeutic potential in EC. Another approach can be the combination of AuNPs with immunotherapy to further suppress EC progression.

### 2.2 AgNPs

AgNPs have garnered considerable interest in treating GI tumors because of their distinctive antimicrobial, anticancer, and physicochemical characteristics, establishing them as a flexible resource in oncology. Their small size, elevated surface area-to-volume ratio, and capacity to be modified with different biomolecules allow for accurate targeting and enhanced therapeutic effectiveness. The main way AgNPs exert their anticancer functions is by triggering oxidative stress, in which the nanoparticles generate ROS that negatively affect cancer cell membranes, DNA, and organelles, resulting in apoptosis or necrosis. Moreover, AgNPs can disrupt cellular signaling pathways, suppressing tumor growth, blood vessel formation, and metastasis. Their capacity to interfere with mitochondrial activity and ATP generation additionally undermines cancer cells, rendering them more vulnerable to standard treatments. In addition to their direct cytotoxic properties, AgNPs facilitate the transport of anti-cancer medications by increasing solubility, stability, and bioavailability, and they also allow for controlled release at the tumor location to reduce systemic side effects. Another important benefit is their capability in PTT, wherein AgNPs capture light and transform it into heat, selectively destroying tumor cells while causing minimal damage to the healthy tissue. Moreover, their natural antimicrobial qualities reduce the risk of infections, which is especially advantageous for GI cancer patients who frequently suffer from decreased immunity due to chemotherapy or surgery. The importance of AgNPs in treating GI cancer stems from their multifunctionality; they act as therapeutic agents and drug carriers, while also enhancing diagnostics via imaging methods such as SERS or CT. Moreover, their natural antimicrobial qualities reduce the risk of infections, which is especially advantageous for GI cancer patients who frequently experience weakened immunity due to chemotherapy or surgery. The importance of AgNPs in treating GI cancer stems from their multifunctionality; they function as therapeutic agents and drug carriers, while also enhancing diagnostics via imaging methods such as SERS or CT. Despite these benefits, issues including possible toxicity, long-term biocompatibility, and ideal dosing require to be tackled to guarantee safe clinical implementation. Nonetheless, incorporating AgNPs into GI oncology offers a hopeful approach to boost treatment results, decrease resistance, and improve the accuracy of cancer treatment.

#### 2.2.1 CRC

CRC exhibits a significant prevalence and considerable resistance to standard treatments. Curcumin (CUR), a natural chemical, has shown significant potential for the treatment of CRC *in vitro*; nevertheless, its clinical applicability is constrained by inadequate bioavailability. To resolve this issue, CUR was integrated into hydrogels formulated with chitosan (CHT) and chondroitin sulfate (CS) natural biopolymers that provide regulated and controlled release. The hydrogels were generated in ionic liquids ([Hmim][HSO_4_]), which augment the solubility of CHT and increase the overall characteristics of the hydrogel system. Furthermore, CUR was amalgamated with AgNPs and activated by visible light using PDT, whereby the metal-enhanced singlet oxygen (MEO) action precipitates the demise of cancer cells. The production of AgNPs using a green technique includes an ultrasonic bath. The CHT/CS hydrogels infused with CUR/AgNPs were meticulously characterized. The cellular experiments validated the non-cytotoxic properties of the hydrogels concerning healthy tissues. During selective illumination in PDT, the CHT/CS/CUR-AgNPs hydrogel significantly suppressed Caco-2 human colon cancer cells (CC_50_ = 91.5 μg/mL of hydrogel). Cellular uptake investigations demonstrated that CUR not only provides therapeutic benefits but also serves as a fluorescent probe for diagnostic purposes, hence providing a theranostic strategy. The use of reagents, solvents, and techniques conformed to the tenets of green chemistry [52]. Focusing on CRC, recent research has integrated AgNPs into multifunctional delivery systems to enhance both therapeutic and diagnostic potential. The anticancer actions of AgNPs in CRC were examined utilizing HCT116 cells and xenograft mice models. AgNPs were found to cause apoptosis by enhancing mitochondrial ROS generation, causing mitochondrial malfunction, and eliciting endoplasmic reticulum (ER) stress via NOX4. Pretreatment with diphenyleneiodonium (DPI) or 4-phenylbutyric acid (4-PBA) significantly decreased ROS generation, ER stress, mitochondrial impairment, and apoptosis, signifying the engagement of these mechanisms. Tumor proliferation in xenograft models was significantly inhibited by AgNPs via the activation of apoptosis ER stress responses. The anticancer effects of AgNPs were mediated through ROS- and ER stress-related mitochondrial apoptotic pathways [53]. Expanding from therapy to diagnostics, innovative biosensing platforms using AgNPs have been designed for highly sensitive detection of biomarkers.

#### 2.2.2 Liver cancer (HCC)

The advancement of nanomaterial-enhanced electrochemical sensors has become increasingly relevant owing to their extensive surface area. AgNPs are particularly appealing for biological sensing due to their cost-effectiveness, non-toxicity, biocompatibility, stability, and superior catalytic characteristics. Propolis is a naturally occurring, viscous material consisting of roughly 45% resin, 35% wax, and 20% inert chemicals. It is non-toxic, biocompatible, and possesses strong adhesive qualities. A carbon paste electrode modified with AgNPs and propolis (APCPE) was created as a highly sensitive and selective electrochemical biosensor for detecting miRNA-let 7a, a tumor-suppressor biomarker linked to HCC. Optimal conditions were determined at 25 °C with a 30-min incubation period. Electrochemical study revealed substantial changes following hybridization with the target miRNA, achieving a detection limit of 10^−3^ fM. The biosensor was utilized on blood samples from healthy persons, HCC patients, and liver cancer cell lines (Huh7, HepG2), demonstrating miRNA expression levels. Elevated selectivity was observed even in the presence of incongruent sequences. Enhanced sensitivity and operational simplicity were attained relative to the reported approaches, indicating its significant promise for clinical miRNA detection [54]. In line with this, another electrochemical approach has been introduced, leveraging lipid-embedded AgNPs to achieve precise nucleic acid detection even in complex biological fluids. An innovative electrochemical technique is introduced for the precise and selective identification of HCC up-regulated long non-coding RNA (HULC), a long non-coding RNA linked to liver cancer, utilizing a combination of antifouling lipid bilayer coatings and AgNPs as signaling probes. The LB, self-assembled on the gold electrode surface, emulates a cell membrane and significantly inhibits non-specific adsorption of proteins and other serum constituents, hence assuring excellent detection in complicated biological fluids. Two DNA probes, aDNA and cDNA, are engineered to partly hybridize with HULC, and upon target identification, they develop a Y-shaped structure that attracts AgNPs to the electrode surface, generating a robust electrochemical signal. The effective assembly and antifouling characteristics of the LB, the immobilization of cholesterol-modified DNA probes, and the conjugation of cDNA to AgNPs were confirmed using electrochemical impedance spectroscopy (EIS), fluorescence microscopy, surface plasmon resonance (SPR), UV–Vis spectroscopy, and TEM. The development of the Y-shaped structure was also validated by polyacrylamide gel electrophoresis (PAGE) and fluorescence quenching experiments. The electrochemical response, assessed using linear sweep voltammetry (LSV), demonstrated a robust association with HULC concentration in the 1–500 fM range, exhibiting exceptional sensitivity (LOD = 0.42 fM), repeatability (RSD = 2.68%), and stability (almost 90% signal retention after 21 days). Specificity assays with mutant and mismatched HULC sequences validated the method's exceptional selectivity. The approach exhibited consistent performance in both diluted and undiluted serum, showcasing its strong antifouling properties and suitability for direct use in clinical diagnostics. The platform's architecture allows straightforward customisation for the detection of various nucleic acids by the alteration of probe sequences, indicating extensive use in biomedical diagnostics [55].

Complementing synthetic approaches, biosynthesis methods have been explored, using bacteria to produce AgNPs demonstrating both antibacterial and anticancer properties. Green nanomaterials have obtained significant attention for their potential as medicinal agents. A study developed AgNPs with a silver-resistant *Bacillus safensis* TEN12 strain, isolated from metal-contaminated soil and identified via 16S rRNA gene sequencing. The existence of AgNPs in the bacterial culture was confirmed using UV–vis spectroscopy, which exhibited an absorption peak at 426.18 nm. FTIR spectroscopy demonstrated the participation of capping proteins and alcohols in the stability of the nanoparticles. X-ray diffraction (XRD), in conjunction with scanning and transmission electron microscopy (SEM and TEM), validated the crystalline structure and spherical shape of the AgNPs, with particle sizes between 22.77 and 45.98 nm. Energy-dispersive X-ray spectroscopy (EDX) examination revealed a silver concentration of 93.54% in the nano-powder. At a dosage of 20 μg/mL, the AgNPs demonstrated substantial antibacterial efficacy, yielding inhibition zones of 20.35 mm and 19.69 mm against *Staphylococcus aureus* and *Escherichia coli*, respectively, while significantly decreasing bacterial density in broth culture. The AgNPs exhibited significant anticancer efficacy against the HepG2 human liver cancer cell line in MTT experiments, while displaying minimal cytotoxicity towards the normal human embryonic kidney cell line (HEK293) [56].

Further supporting the promise of green synthesis, plant-extract-derived AgNPs have also demonstrated remarkable anticancer activity in both *in vitro* and *in vivo* liver cancer models. HCC, the predominant type of primary liver cancer, is strongly associated with chronic liver injury and inflammation, with more than 90% of cases arising under these circumstances. Chronic inflammation significantly influences the development and severity of hepatic malignancies. Recent breakthroughs have highlighted the remarkable anticancer potential of nanomaterials, especially AgNP derived from plant-based sources. A study investigated the biosynthesis, characterization, and assessment of the anticancer efficacy of AgNPs included with *Madhuca longifolia* extract (MLAgNPs) in a rat model of hepatic carcinoma. *M. longifolia* is abundant in flavonoids and phenolic compounds, recognized for their medicinal effects. A single dosage of diethylnitrosamine (DEN) (200 mg/kg body weight) was delivered to 36 Wistar rats to develop hepatic carcinoma. Treatment with MLAgNPs demonstrated a dose-dependent decrease in observable liver abnormalities compared to rats administered just with DEN. Subsequent evaluations were performed on serum and hepatic tissues to examine antioxidant concentrations, inflammatory cytokines, and histological alterations. The results demonstrated that MLAgNP therapy significantly decreased proinflammatory cytokines, including tumor necrosis factor-alpha (TNF-α), interleukin-6 (IL-6), interleukin-1 beta (IL-1β), and nuclear factor-kappa B (NF-κB). Moreover, there was an improvement in the activity of membrane-bound enzymes. Histopathological assessments demonstrated partial morphological changes in the liver tissues of MLAgNP-treated groups [57].

Despite the promising advances in AgNP-based approaches for liver cancer detection and treatment, several significant limitations persist that must be addressed before clinical implementation. The electrochemical biosensors, while demonstrating exceptional sensitivity with detection limits as low as 10^−3^ fM for miRNA-let 7a and 0.42 fM for HULC, face substantial challenges in terms of standardization and reproducibility across different laboratory settings (pre-clinical) and clinical environments (clinical). The reliance on precise incubation conditions (25 °C for 30 min) and the requirement for specialized electrochemical equipment may limit their accessibility in resource-constrained healthcare settings. Furthermore, while the antifouling lipid bilayer coatings show promise in preventing non-specific protein adsorption, the long-term stability and performance of these complicated multi-component systems under varying physiological conditions remain incompletely characterized. The biosynthesis approaches using bacterial strains such as *Bacillus safensis* TEN12, although environmentally friendly, suffer from batch-to-batch variability in nanoparticle size distribution (22.77–45.98 nm) and potentially inconsistent bioactivity. Additionally, the translation from promising *in vitro* results to clinical efficacy faces the challenge of biocompatibility assessment, as even "green" synthesized AgNPs may accumulate in the tissues and potentially cause long-term toxicological effects that are not yet fully understood. The selectivity claims, while supported by laboratory studies with specific mutant sequences, may not hold true against the complex background of genetic variations present in diverse patient populations. The future of silver nanoparticle applications in liver cancer management holds tremendous potential through the integration of emerging technologies and novel synthetic approaches that could revolutionize both diagnostic and therapeutic paradigms. Advanced surface engineering techniques are expected to enable the development of multi-functional AgNPs that combine diagnostic, therapeutic, and real-time monitoring capabilities within a single platform, potentially incorporating stimuli-responsive elements that can be activated by specific TME conditions such as pH, hypoxia, or enzymatic activity. The convergence of artificial intelligence and machine learning with electrochemical sensing could lead to the predictive biosensor systems capable of not only detecting current biomarker levels but also forecasting disease progression patterns based on temporal biomarker fluctuations. Personalized medicine approaches may leverage patient-specific biomolecular profiles to customize AgNP surface modifications, ensuring optimal targeting efficiency and minimizing off-target effects. Novel green synthesis methodologies using engineered microorganisms or plant cell cultures could provide unprecedented control over nanoparticle characteristics, potentially enabling the production of AgNPs with programmable size, shape, and surface properties tailored for specific therapeutic applications. The integration of AgNPs with emerging delivery systems such as extracellular vesicles or cell-penetrating peptides could enhance their ability to cross biological barriers and achieve targeted intracellular delivery. Furthermore, the development of biodegradable AgNP formulations that can be completely metabolized and eliminated from the body could address current toxicity concerns while maintaining therapeutic efficacy, paving the way for safer long-term treatments and repeated diagnostic procedures in chronic liver disease management.

#### 2.2.3 GC

The green synthesized silver nanoparticles using Ardisia gigantiflia extract (Arg-AgNPs) were effectively generated utilizing *Ardisia gigantifolia* leaf extract, revealing the presence of spherical nanoparticles with an average diameter of 6 nm. These nanostructures were able to suppress GC through decreasing proliferation (IC_50_ values < 1.4 μg/mL), cell cycle arrest in the G0/G1 phase, and a substantial reduction in migration. Cellular senescence was increased, since it was demonstrated by increased senescence-associated beta (SA-β)-galactosidase activity, and intracellular ROS levels were significantly enhanced in a dose-dependent manner, showing that oxidative stress is a principal mechanism behind these anti-cancer effects [58]. To build on these findings, another study demonstrated a rapid, eco-friendly synthesis of AgNPs using plant extracts, exploring both antimicrobial and anticancer effects. An effective, fast, and ecologically sustainable method has been established for the development of AgNPs via the solution plasma technique with *Paramignya trimera* (*P. trimera*) extracts. The effect of *P. trimera* extract concentration and applied voltage on AgNP synthesis was investigated. The surface plasmon resonance spectra demonstrated a significant peak at 413 nm, signifying effective nanoparticle development. FTIR validated the existence of functional groups linked to the produced AgNPs. Morphological study indicated that the nanoparticles were spherical, with an average diameter of around 8 nm. AgNPs synthesized using *P. trimera* extract exhibited superior stability in solution compared to those prepared without the extract. A mechanism for the design of AgNPs is presented. The produced AgNPs exhibited notable antibacterial efficacy against both Gram-positive *Staphylococcus aureus* and Gram-negative *Pseudomonas aeruginosa*, as well as pronounced anticancer effects on the AGS stomach cancer cell line. These findings highlight an efficient and environmentally friendly alternative to the traditional chemical reduction procedures for the synthesis of AgNPs, with significant biological implications [59]. Continuing this exploration and providing more novel treatments using nanoparticles, research compared phytosynthesized AgNPs with commercial counterparts, highlighting their superior anticancer efficacy against GC. AgNPs were biosynthesized utilizing *Artemisia turcomanica* leaf extract, and their anticancer efficacy against AGS stomach cancer cells was assessed. The synthesis of AgNPs was validated by visual color change, UV–Vis spectroscopy (exhibiting an absorption peak at 430 nm), XRD analysis (demonstrating characteristic face-centered cubic peaks), FT-IR spectroscopy (indicating interactions with functional groups), and TEM/SEM imaging (showing spherical particles averaging approximately 22 nm), complemented by EDX analysis (identifying a pronounced Ag signal at 3 keV). Phytosynthesized AgNPs demonstrated superior cytotoxicity compared to the commercial AgNPs, evidenced by a reduced IC_50_ value (4.88 μg/mL versus 6.37 μg/mL) in AGS cells, and elicited elevated apoptosis levels with less necrosis. Gene expression study revealed a significant elevation in BAX and a reduction in Bcl2 expression subsequent to treatment with biosynthesized AgNPs. Selectivity for cancer cells was noted, as a threefold greater dosage was required to inhibit normal fibroblast (L-929) cells. Therefore, AgNPs generated from *A. turcomanica* exhibited superior pro-apoptotic activity and increased anticancer effectiveness relative to other plant-derived or commercial nanoparticles [60]. Turning attention to PC, green-synthesized AgNPs have been investigated for their cytotoxic potential across several pancreatic tumor cell lines.

#### 2.2.4 PC

PC has a very high mortality rate and is influenced by several risk factors, including smoking, chronic pancreatitis, long-standing diabetes, genetic disorders, and possible environmental or lifestyle factors. The global burden of pancreatic cancer is expected to rise due to increasing smoking rates in developing countries, improved diagnostic methods, and longer population life expectancy [61]. A therapy technique uses nanoparticle to directly transport therapeutic drugs to PC cells, therefore reducing damage to healthy tissues and increasing selectivity towards cancer cells. AgNPs have exceptional efficacy owing to their robust light absorption in the NIR spectrum. A study involved the introduction of IgG-functionalized AgNPs to PC cell lines. The cells were subsequently subjected to an 808 nm, 2-W laser. The PTT with IgG-functionalized AgNPs disrupted the Golgi apparatus, hence activating the caspase-3 apoptotic pathway, which eventually resulted in the death of PC cells [62]. Further mechanistic studies explored the multiple cell death pathways activated by AgNPs, providing insights into their diverse anticancer actions in PC. AgNPs exhibit extensive biological activity and unique characteristics, rendering them potential solutions to prevalent challenges related to the chemotherapy resistance. A study examined the cytotoxic efficacy of AgNPs on PC cells and analyzed the molecular processes behind their effects. For a comparative analysis, their toxicity on non-malignant pancreatic cells (hTERT-HPNE) was evaluated. AgNPs, particularly those measuring 2.6 nm and 18 nm in diameter, significantly reduced PC cell viability and proliferation in a manner contingent upon both particle size and concentration. Ultrastructural analyses demonstrated that the internalization of AgNPs induced many types of cell death, including apoptosis, autophagy, necroptosis, and mitotic catastrophe. The cellular alterations were associated with an overexpression of the pro-apoptotic protein Bax and a downregulation of the anti-apoptotic protein Bcl-2. Furthermore, AgNPs significantly elevated the levels of the tumor suppressor protein p53, as well as proteins linked to necroptosis (receptor-interacting protein kinase 1 (RIP-1), receptor-interacting protein kinase 3 (RIP-3), mixed lineage kinase domain-like protein (MLKL)) and autophagy microtubule-associated protein 1 light chain 3-II (LC3-II)), indicating the activation of several cell death pathways [63]. Despite these promising developments, challenges associated with toxicity, dosing, and reproducibility persist as significant hurdles to the clinical application of AgNPs in GI oncology.

Although AgNPs show promising anticancer potential in GI oncology, various challenges impede their clinical application. A major concern is the possible toxicity and long-term biocompatibility of AgNPs. Numerous studies indicate that healthy cells experience minimal cytotoxic effects at specific concentrations, but sustained exposure or accumulation of nanoparticles in essential organs can result in oxidative stress, inflammation, and unintended harm to tissues. Moreover, differences in particle size, surface charge, and synthesis techniques, be they biological, chemical, or physical can greatly affect toxicity characteristics. Standardizing production methods to guarantee reproducibility, purity, and stability is essential yet challenging due to the variability inherent in green synthesis techniques that employ plant extracts or microbial systems. Besides, comprehending the exact interactions between AgNPs and biological systems, such as protein corona formation and immune reactions, is crucial to avoid unexpected negative effects. A further major challenge is optimizing dosing schedules and delivery methods to balance between effectiveness and safety. The aggregation tendency of AgNPs, their rapid removal by the mononuclear phagocyte system, and possible off-target effects prevent their therapeutic use. Although functionalization techniques such as attaching AgNPs to ligands, antibodies, or polymers improve tumor selectivity and reduce systemic exposure, these modifications complicate the production process and increase regulatory challenges.

### 2.3 MOFs

#### 2.3.1 CRC

MOFs are porous materials consisted of metal ions or clusters alongside organic ligands, offering advantages such as biocompatibility, large surface areas, and customizable pore structures. Their rich surface properties enable consistent interactions with biological macromolecules, making them beneficial for biomolecule detection. A highly sensitive and selective quartz crystal microbalance (QCM) biosensor device was developed for the detection of minuscule amounts of miR-221, a biomarker linked to early colon cancer screening. The detection technique utilized nanoscale UiO-66-NH_2_ MOFs, which were electrostatically attached to streptavidin (SA) to create a stable SA@UiO-66-NH_2_ complex. This compound was engineered to engage with biotin-modified hairpin probe DNA (PDNA) affixed to a gold-coated QCM chip. Upon hybridization with the target miR-221, the PDNA experienced a structural alteration that revealed the biotin site, facilitating its interaction with the SA@UiO-66-NH_2_ complex and resulting in a measurable frequency shift. Different UiO-66-NH_2_ particles, varying in size and surface charge, were synthesized and analyzed. The smaller, positively charged particles (UiO-66-NH_2_-85) demonstrated enhanced signal amplification owing to their more efficient adhesion to the sensor surface. Exceptional repeatability, precision, and specificity were validated, encompassing the capacity to differentiate between single and double base mismatches and to operate efficiently in diluted serum samples. To enhance detection sensitivity, the system was integrated with an exponential amplification reaction (EXPAR), which lowered the detection limit from 6.9 fM to 0.79 aM and facilitated quantification throughout a dynamic range of 1 aM to 1 nM, exhibiting a robust linear correlation (*R*^2^ > 0.99). This twofold amplification technique demonstrated that the QCM biosensor is effective for the trace detection of miR-221 and has potential as a significant instrument for the early clinical diagnoses of miRNA-related disorders [64].

Expanding on this, innovative MOF-based systems have been developed to overcome tumor hypoxia, offering enhanced PDT and synergies with immunotherapy. Immunotherapy has emerged as a potential modality for cancer treatment, however it remains efficacious for only a select group of patients. Immunogenic PDT might potentially improve immunotherapy response rates by activating the immune system; however, its efficacy is frequently obstructed by tumor hypoxia. A nanoscale MOF (nMOF) known as Fe-TBP, has been designed as an innovative nanophotosensitizer aimed at overcoming this constraint and improving PDT effectiveness, thereby preparing non-inflamed tumors for immunotherapy. Composed of iron-oxo clusters and porphyrin ligands, Fe-TBP facilitates PDT in both normoxic and hypoxic environments. In preclinical models of CRC, Fe-TBP-mediated PDT significantly enhanced the efficacy of anti-programmed death-ligand 1 (α-PD-L1) therapy and elicited strong abscopal effects, resulting in tumor regression rates over 90%. Mechanistic studies revealed that Fe-TBP-mediated PDT significantly enhances the infiltration of cytotoxic T lymphocytes into malignancies [65].

**Fig. 4:**
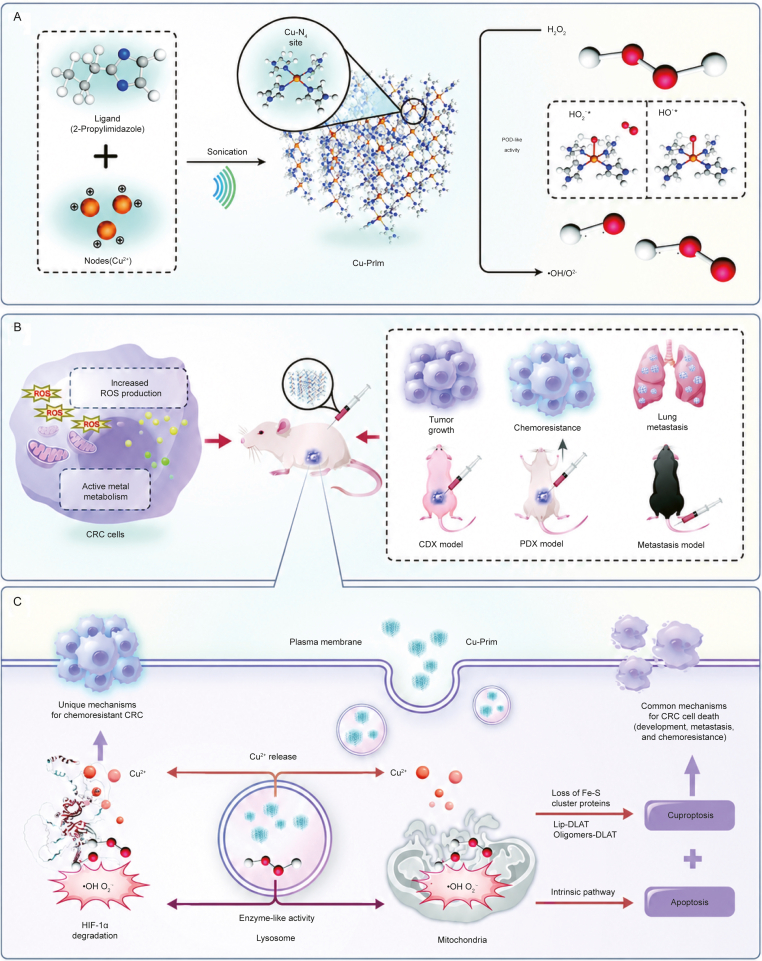
Schematic illustration of copper-based Prussian blue-like metal-organic framework (Cu-PrIm) nanozymes for treating colorectal cancer (CRC). (A) Cu-PrIm nanozymes were produced through a simple self-assembly method aided by sonication. The modified Cu-N4 catalytic site enables Cu-PrIm to exhibit enzyme-like functionality, generating reactive oxygen species (∙OH and ∙O^2−^). (B, C) Cu-PrIm nanozymes exhibited significant antitumor effectiveness in various situations, including tumor growth, chemoresistance, and lung metastasis in CRC. The nanozymes activated two cell death pathways, apoptosis and cuproptosis, through their peroxidase (POD)-like function and controlled release of copper ions. 2-PI: 2-propylimidazole; ROS: reactive oxygen species; CDX model: cell-derived xenograft model; PDX model: patient-derived xenograft HIF-1α: hypoxia-inducible factor 1-alpha; DLAT: dihydrolipoamide acetyltransferase; Lip-DLAT: lipoylated DLAT; Oligomers-DLAT: oligomerized DLAT. Reproduced withpermission from Ref. [66].

Notwithstanding substantial advancements in CRC therapy, the overall survival rate for individuals with advanced disease stages remains around 50%. This is mostly attributable to ongoing challenges such as treatment resistance and metastasis. In addressing these issues, a novel "Three-in-One" copper-based MOF nanozyme has been developed that emulates peroxidase (POD) activity. This nanozyme, designated Cu-PrIm, is designed to concurrently disturb oxidative stress equilibrium and copper ion homeostasis in CRC cells, therefore triggering cell death via both apoptosis and cuproptosis. Furthermore, it facilitates the degradation of hypoxia-inducible factor 1α (HIF-1α), which is crucial in mitigating chemoresistance. Cu-PrIm nanozymes consist of copper and 2-propylimidazole, creating a deformed Cu-N4 catalytic core that structurally mimics normal enzymes containing copper and histidine. This arrangement imparts the nanozymes with robust enzyme-like characteristics. Their anticancer efficacy has been rigorously confirmed using *in vivo* CRC models. Furthermore, these nanozymes exhibit remarkable biocompatibility, biodegradability, and low toxicity in murine experiments, highlighting their potential for the future therapeutic applications. This work introduces an innovative therapeutic approach for CRC by utilizing Cu-PrIm nanozymes to concurrently address several cancer vulnerabilities, hence providing a potent mechanism for improving treatment efficacy and overcoming drug resistance (Fig. 4) [66].

Nanoscale metal–organic frameworks (nanoMOFs) have been thoroughly investigated for drug delivery; however, their inherent bioactivity, especially anticancer efficacy, is often overlooked due to insufficient attention to the synergistic roles of their hierarchical components, including nanosized MOFs, organic ligands, and metal–organic complexes. A copper–olsalazine (Olsa) nanoMOF was constructed utilizing Olsa as both a bioactive linker and a DNA hypomethylating agent. This Cu-Olsa nanoMOF demonstrates a complicated anticancer mechanism that includes redox dyshomeostasis to improve catalytic tumor treatment, inhibition of cyclooxygenase-2 (COX-2) via the Cu^2+^-Olsa combination, and epigenetic regulation facilitated by Olsa. The nanoMOF exhibits enzyme-mimetic activity, producing deadly hydroxyl radicals (·OH) and singlet oxygen (^1^O_2_) from plentiful hydrogen peroxide in tumors. Furthermore, it enhances the expression of tumor suppressor genes including TIMP3 and AXIN2 via epigenetic regulation and specifically targets CRC cells while preserving normal cells. The alteration of HA further improves tumor-targeting efficacy, leading to the significant suppression of CT26 CRC development and metastasis in mouse models. The findings highlight the promise of Olsa-based MOFs as nanomedicines synergized with epigenetic therapy, providing a solid framework for the development of inherently bioactive nanoMOFs for effective cancer treatment [67].

**Fig. 5:**
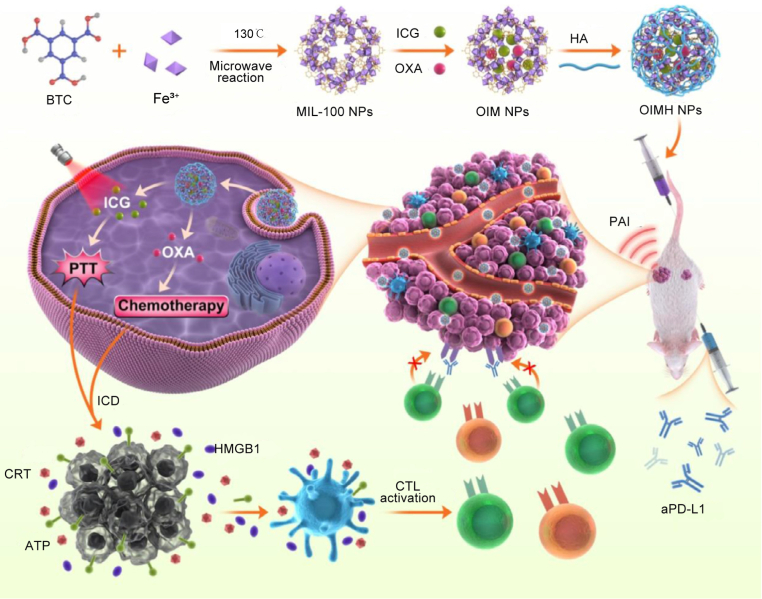
Hyaluronic acid (HA)-coated OXA/ICG-loaded MIL-100 nanoparticles (OIMH NPs)-driven photoacoustic imaging-guided chemophotothermal treatment. Upon the synthesis of nanoparticles and loading drugs including indocyanine green (ICG) and oxaliplatin (OXA), their functionalization with HA has been performed. These nanoparticles can provide a combination of chemotherapy and photothermal therapy to trigger immunogenic cell death (ICD). Such an event releases calreticulin (CRT), adenosine triphosphate (ATP), and high mobility group box 1 (HMGB1) to stimulate cytotoxic T lymphocytes (CTLs) in cancer immunotherapy. BTC: 1,3,5-benzenetricarboxylic acid; MIL-100 NPs: metal-organic framework nanoparticles; PTT: photothermal therapy; aPD-L1: programmed death-ligand 1 antibody; PAI: photoacoustic imaging. Reprinted with permission from Ref. [69].

A MOF composed of tetrakis(4-carboxyphenyl)porphyrin (TCPP) and Fe^3+^ was effectively synthesized, exhibiting a nano-shuttle shape and stability in physiological environments. Upon exposure to H_2_S, a characteristic of the CRC microenvironment, the MOF deteriorated, releasing fluorescent TCPP and reinstating its photosensitivity. This facilitated precise fluorescence imaging and efficient singlet oxygen production under laser irradiation. *In vitro*, the MOF preferentially activated in CRC cells, producing ROS and inducing substantial cytotoxicity following light exposure, while exhibiting safety in normal cells. *In vivo* investigations validated tumor-specific accumulation, extended fluorescence retention, efficient PDT targeting tumors, and no systemic damage. Consequently, this H_2_S-activatable MOF has significant potential as a CRC-specific theranostic drug for fluorescence-guided photodynamic therapy [68]. Expanding on MOF-based strategies, multifunctional nanoparticles combining chemotherapy and PTT were developed, aiming to enhance both imaging precision and immune activation in CRC treatment. Multifunctional nanoparticles (OIMH NPs) were synthesized via a straightforward one-step encapsulation method to co-deliver oxaliplatin (OXA) and ICG, utilizing HA-modified MIL-100 NPs as the carrier, with the objective of integrating chemotherapy and PTT for improved CRC treatment. Characterization validated the effective drug loading and maintenance of MIL-100's crystalline structure, whereas HA coating enhanced dispersion, stability under physiological circumstances, and regulated drug release. The OIMH NPs exhibited significant photothermal effects under 808 nm NIR irradiation, demonstrating enhanced photothermal conversion efficiency and stability relative to free ICG. The *in vitro* combination of OXA and PTT had a significantly enhanced cytotoxic impact on CT26 tumor cells compared to either treatment alone, facilitating ICD via elevated production of damage-associated molecular patterns (DAMPs) such as HMGB1, CRT, and ATP. *In vivo*, OIMH nanoparticles facilitated efficient tumor accumulation and prolonged drug retention, resulting in considerable tumor suppression in CT26 tumor-bearing mice with minimal toxicity. This synergistic treatment elicited strong immunological responses characterized by increased infiltration of CD^4+^ and CD^8+^ T cells, as well as a rise in effector memory T cells (Tem) in the spleen, hence fostering immunologic memory and inhibiting tumor recurrence. Furthermore, the integration of OIMH NPs-mediated chemotherapy-PTT with aPD-L1 immune checkpoint inhibition exhibited a pronounced abscopal impact, suppressing both primary and metastatic tumors by ameliorating the immunosuppressive TME and augmenting systemic antitumor immunity. This advanced, PAI-directed therapy provides a robust framework for accurate CRC treatment through the integration of imaging, targeted therapy, and immune activation to provide lasting therapeutic results (Fig. 5) [69]. To further improve immunotherapy responsiveness, another study engineered a pH-sensitive MOF system integrating metabolic modulation with precise immune activation in CRC. Immunotherapy has shown considerable advancement in provoking systemic anti- CRC responses. A pH-sensitive zeolitic imidazolate framework-8 (CS/NPs) has been designed to augment the responsiveness of CRC to immunotherapy. This nanoplatform facilitates the effective activation of the cGAS-STING pathway and the inhibition of immunological checkpoints by encapsulating the chemotherapeutic drug mitoxantrone (MTX) and the immunomodulator thymus pentapeptide (TP5), while being functionalized with tumor-targeting chondroitin sulfate (CS). In this framework, CS enables precise distribution to tumor locations while reducing systemic toxicity. The coordinated Zn^2+^ significantly impedes glycolysis and diminishes the expression of glucose transporter type 1 (GLUT1), thereby depriving cancer cells of energy. Zn^2+^ promotes the adenosine 5′-monophosphate activated protein kinase (AMPK) pathway, facilitating the degradation of PD-L1 protein and increasing the susceptibility of CRC cells to immunotherapy. Moreover, DNA damage caused by MTX activates the cyclic GMP-AMP synthase-stimulator of interferon genes (cGAS-STING) pathway. This route, with TP5, enhances the proliferation and development of T lymphocytes and dendritic cells, hence intensifying the anti-CRC immune response. CS/NPs augment the efficiency of chemotherapy and elicit strong systemic antitumor immune responses in both *in vitro* and *in vivo* models, presenting a viable technique to increase the effectiveness of CRC immunotherapy [70]. In addition to their applications in treatment, MOF-based platforms have also been leveraged for high-throughput metabolic fingerprinting, offering powerful diagnostic capabilities for early CRC detection. A metabolic shift initiates during the initial phases of CRC development, highlighting its pivotal importance in comprehending disease etiology and improving precision diagnostics. A new molecular method utilizing MOF hybrids has been devised to characterize metabolic alterations in CRC. This technique requires only 500 nL of plasma and eliminates the need for sample preparation, allowing the fast capture of metabolic fingerprints using laser desorption/ionization mass spectrometry in few seconds. A diagnostic model utilizing machine learning, developed from these data, effectively differentiates CRC patients from healthy individuals, attaining an average area under the curve (AUC) of 0.947 in the discovery cohort and 0.912 in an independent validation cohort. Additionally, the model delineates a CRC-specific metabolic signature comprising 34 prospective biomarkers [71].

#### 2.3.2 PC

**Fig. 6:**
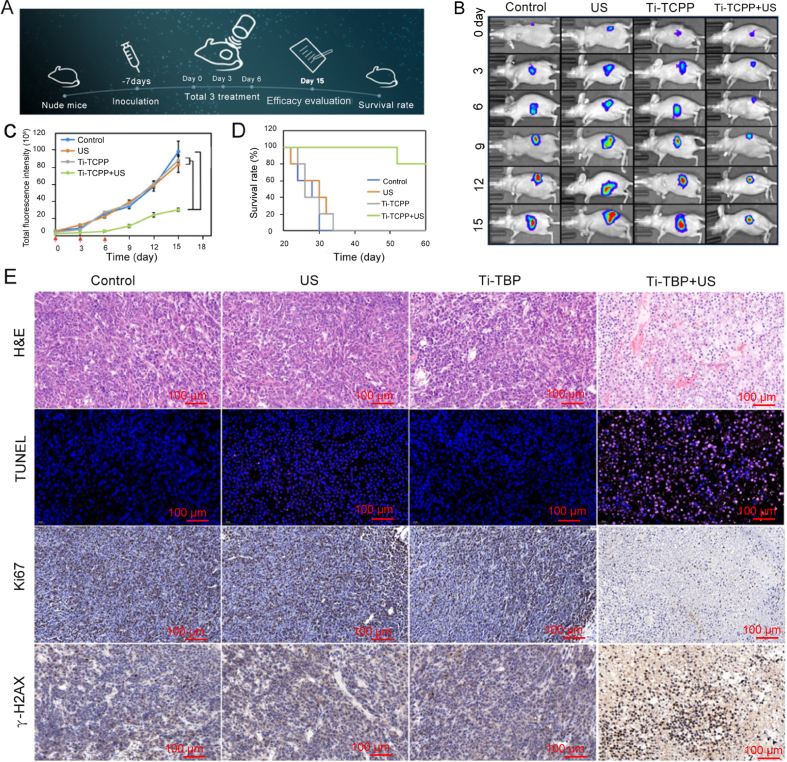
*In vivo* evaluation of cancer-fighting effectiveness. (A) Illustration of the healing process. (B) Fluorescent images of mice with tumors after various treatments. (C) Differences in tumor fluorescence intensity. (D) Survival rates of tumor-affected mice after different therapies. (E) Hematoxylin and eosin stain (H&E), terminal deoxynucleotidyl transferase dUTP nick end labeling (TUNEL), Antigen Kiel 67 (Ki67), and gamma histone (γ-H2AX) staining of tumors 15 days after treatment. Data are represented as means ± standard deviation (SD) (*n* = 5). Reproduced with permission from Ref. [73].

The use of phototherapy is still a promising approach in tumor suppression. The synthesis and assessment of a novel chlorin-based Hf-MOF (MA-HfMOF-PFC-Ni-Zn) functionalized with maltotriose for targeted PDT demonstrated enhanced efficacy relative to its porphyrin-based equivalent (MA-HfMOF-PFP-Ni-Zn) against aggressive cancer cell lines (MDA-MB-231, MIA-PaCa2, HeLa), while demonstrating minimal toxicity to healthy MCF-10a cells. The integration of chlorin linkers and zinc metalation improved singlet oxygen production, while surface modification by SNAr reaction facilitated specific targeting of cancer cells via GLUT receptor recognition, as evidenced by higher cellular uptake and alterations in zeta potential. Characterization using TEM, UV–vis, powder X-ray diffraction (pXRD), and inductively coupled plasma mass spectrometry (ICP-MS) confirmed the effective synthesis and functionalization. These multifunctional MOFs hold potential for targeted cancer therapy and may serve dual purposes as PDT and magnetic resonance imaging (MRI) agents, necessitating further *in vivo* investigations to evaluate biodistribution, clearance, and therapeutic safety [72]. Building on the promise of photosensitizer-loaded MOFs, sonodynamic therapy (SDT) approaches were explored, developing a nucleus-targeting MOF for enhanced treatment of hypoxic pancreatic tumors. An innovative nucleus-targeted ultra-small Ti-tetrakis(4-carboxyphenyl)porphyrin (Ti-TCPP) MOF has been developed to improve SDT for hypoxic malignancies, including PC. In contrast to conventional O_2_-dependent type II SDT systems, this Ti-TCPP MOF enables the formation of both type I and type II ROS under low-intensity ultrasound (0.5 W/cm^2^), preserving effectiveness under oxygen-deficient conditions. It induces DNA damage and S phase cell cycle arrest, resulting in apoptosis. It significantly suppresses orthotopic pancreatic tumor development and prolongs life in treated mice due to extended circulation, elevated tumor accumulation, and targeted nuclear delivery. Furthermore, it is almost entirely eradicated from the body within seven days and has no considerable toxicity, providing a secure and efficient method for SDT (Fig. 6) [73]. To further address the challenges of tumor penetration and immune activation, a novel MOF encapsulating collagenase was introduced, aiming to remodel the extracellular matrix and boost photoimmunotherapy efficacy. Protein-based treatments have considerable promise for addressing complex disorders such as cancer, with a principal issue being the accurate delivery of substantial quantities of bioactive proteins to tumor locations. MOFs are extensively investigated as carriers for protein therapeutics owing to their capacity to encapsulate proteins via noncovalent interactions. Conventional MOFs frequently exhibit restricted loading efficiency and potential metal ion toxicity, impeding their therapeutic use. A tumor-targeted, collagenase-loaded metal-organic framework (PMO^Col^) has been designed to overcome these constraints, utilizing a protein-metal ion-organic ligand coordination method. This system is constructed by meticulously regulating the ratio of metal ions, histidine residues in the protein, and chemical ligands. Consequently, PMO^Col^ attains an exceptional encapsulation efficiency of 80.3 wt% while preserving significant enzymatic activity of collagenase for the modification of the tumor extracellular matrix (ECM) upon arrival to the TME. In comparison to zeolitic imidazolate framework-8 (ZIF-8)-based protein carriers, PMO^Col^ exhibits significantly lower toxicity. Post-administration, the thick extracellular matrix of pancreatic tumors is efficiently destroyed, facilitating immune cell infiltration and mitigating the immunosuppressive tumor microenvironment. Additionally, the use of a photothermal agent with robust NIR absorption at 1064 nm allows PMO^Col^ to augment ICD, stimulate host immunological responses, and generate systemic immune memory that can impede tumor development and recurrence [74].

Two asymmetrical squaraine dyes, squaraine 1 (SQ1) and squaraine 2 (SQ2), were synthesized and evaluated for possible application in PDT, with SQ2 including a dicyanovinyl substitution that improved its chemical stability and ROS generation capacity. Both dyes demonstrated significant absorption inside the phototherapeutic window (650–700 nm), with SQ2 exhibiting a bathochromic shift and an elevated molar extinction coefficient. Owing to inadequate solubility and stability in aqueous conditions, both dyes were effectively encapsulated within hafnium-based MOFs, specifically microporous UiO-66(Hf) and hierarchically porous HPU-2 and HPU-18, with encapsulation efficiency corresponding to MOF pore dimensions HPU-18, possessing the largest pores, achieved the highest dye entrapment. The functionalization of HPU-18 with bromine and hydroxyl groups enhanced dye compatibility and ROS generation under physiological settings. Following encapsulation, UV–vis tests verified the development of J-aggregates within MOF pores, improving water solubility and maintaining photophysical activity. ROS generation experiments indicated that both SQ1 and SQ2 maintained their capacity for ROS production upon MOF inclusion, with SQ2 surpassing SQ1 owing to its superior structural stiffness and dipole moment. Cytotoxicity evaluations revealed superior biocompatibility of both pure and dye-impregnated MOFs at concentrations up to 200 μg/mL. A multivariate design of experiment (DoE) model was utilized to comprehensively assess the phototoxicity parameters, demonstrating that SQ2@HPU-18(Br) was the most effective formulation, eliciting substantial phototoxic effects even with brief irradiation periods (5 min) and sustaining efficacy for 72 h. This signifies the inaugural incorporation of squaraine dyes into Hafnium-based hierarchical MOFs for PDT, providing an innovative and highly efficient framework for targeted cancer treatment with adjustable photochemical properties and improved biological relevance [75]. To complement PDT-focused MOF platforms, chemotherapeutic limitations were addressed by encapsulating gemcitabine (GEM) within Fe(III)-based MOFs, aiming to enhance its delivery and efficacy in PC. GEM, a nucleoside analogue employed in cancer therapy, is constrained by suboptimal pharmacokinetics, fast breakdown by cytidine deaminase, the development of drug resistance, and schedule-dependent toxicity. To mitigate these concerns, GEM can be encapsulated in a suitable formulation to protect it from metabolic breakdown and fast urine excretion. This study involved the synthesis and evaluation of several biocompatible iron (Fe-III)-based MOFs, namely MIL-101-NH_2_ (rigid), MIL-88A, and MIL-53 (flexible), which varied in topology, connectivity, and chemical composition. MIL-53 was recognized as the most promising carrier for GEM, providing enhanced drug encapsulation and prolonged release. The release of the drug from MIL-53 adhered to zero-order kinetics, sustaining effective plasma concentrations within the therapeutic range. *In vitro* cytotoxicity experiments utilizing PC cell lines (MIAPaCa-2 and PANC1) showed that GEM encapsulated in MIL-53 (MIL53-GEM) exhibited superior cytotoxicity relative to free GEM. The regulated release profile of MIL53-GEM protected the drug against enzymatic degradation, enhancing its effectiveness, half-life, and bioavailability, while *in vivo* investigations demonstrated no major organ damage [76]. Further investigations examined how metabolic conditions, such as ketogenic environments, amplify the therapeutic impact of GEM-loaded MOFs, thereby deepening insights into their anticancer mechanisms.

The treatment of PC is impeded by inadequate therapeutic responses, resistance to chemotherapy, tumor heterogeneity, and extended treatment durations, highlighting the pressing necessity for novel strategies. This research examined the efficacy of gemcitabine-encapsulated MOFs in inhibiting the proliferation of MIA-PaCa-2 PC cells in ketogenic circumstances. GEM was effectively contained within MOFs. Various treatment groups were evaluated for their effects on cytotoxicity, apoptosis, cell cycle distribution, oxidative stress indicators (superoxide dismutase (SOD), glutathione peroxidase (GPx), and malondialdehyde (MDA)), and cell migration. The GEM-MOF combination significantly enhanced apoptosis, caused cell cycle arrest at the sub-G1 phase, diminished antioxidant enzyme activity, raised MDA levels, and suppressed cell migration. The effects were further amplified in ketogenic circumstances, marked by glucose limitation and the presence of beta-hydroxybutyrate, leading to increased cell cycle arrest, oxidative stress, and diminished migratory activity. GEM-MOF therapy, especially when combined with ketogenic conditions, has potential as a more efficacious therapeutic method for PC [77]. Shifting toward combination therapies, a novel thorium-based nMOF was introduced to synergize radiotherapy and radiodynamic treatment, offering potent tumoricidal effects in CRC and pancreatic models. nMOFs composed of high atomic number (high-Z) metals and light-sensitive ligands might enhance radiation-induced tumor eradication via a synergistic radiotherapy and radiodynamic treatment (RT-RDT) mechanism. This study introduces a thorium-based nMOF, Th-DBP, which consists of Th_6_-oxo clusters and 5,15-di(p-benzoato)porphyrin (DBP) ligands, developed by Monte Carlo simulations to improve RT-RDT effects. The thorium-based framework offers enhanced radiation dose amplification compared to the hafnium-based alternative, owing to thorium's superior mass attenuation characteristics. Under X-ray or γ-ray exposure, Th-DBP enhanced energy absorption, augmented ROS generation, and produced much more cancer cell mortality compared to the previously examined Hf-DBP nMOF. Th-DBP significantly suppressed tumor progression when administered with low-dose X-ray irradiation, resulting in an 88% reduction in growth in a colon cancer model and a 97% reduction in a PC model in mice [78]. Additionally, the mechanochemical encapsulation of GEM within aluminum-based MOFs has been explored to enable sustained drug release, yielding promising *in vitro* results for prolonged PC treatment. GEM is a commonly utilized antimetabolite agent featuring a pyrimidine structure, capable of existing in its free-base molecular form. Encapsulated drug formulations are attracting interest for their capacity to facilitate delayed and localized drug release. This study presents the inaugural use of a new mechanochemical technique, liquid-assisted grinding (LAG) to enclose GEM within a porphyrin-based aluminum MOF known as Al-MOF-TCPPH_2_ (designated as compound 2). The chemical interaction between GEM and compound 2 was examined utilizing ATR-FTIR spectroscopy and powder X-ray diffraction (XRD). The encapsulation involves the C=O functional group of GEM, hence validating the development of a composite complex. The release kinetics of GEM from the composite were assessed in PBS at 37 °C utilizing an automated drug dissolving apparatus equipped with an autosampler. Drug concentrations were quantified using high-performance liquid chromatography–ultraviolet (HPLC-UV). The composite displayed a delayed release of GEM due to its bonded form, sustaining a consistent concentration throughout time, while the unencapsulated GEM dissipated swiftly after 45 minmin. The release kinetics of GEM from the composite adhered to a pseudo-first-order rate law. Furthermore, the release mechanism was examined for the first time by altering the stirring speed of the release medium, demonstrating that the kinetic rate constant (k) diminished with decreased stirring speed, signifying a diffusion-controlled release process. The prolonged lethal effects of GEM on PANC-1 PC cells were assessed over six days utilizing the xCELLigence real-time cell analyzer (RTCA). The results indicated a significant disparity between the composite and pure GEM, further corroborating the continuous release pattern [79].

#### 2.3.3 Liver cancer (HCC)

**Fig. 7:**
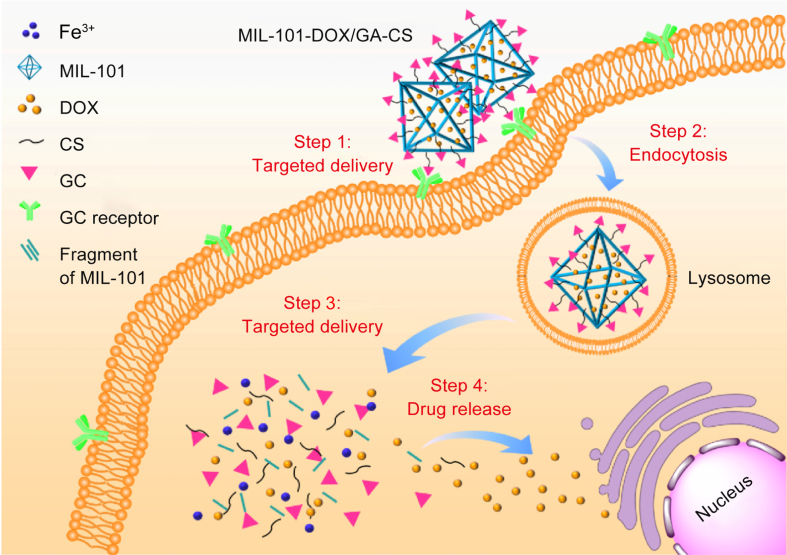
The targeted delivery and pH-responsive release mechanism of metal-organic framework MIL-101-doxorubicin/glycyrrhetinic acid-chitosan (MIL-101-DOX/GA-CS) for hepatocellular carcinoma (HCC) therapy. There are three major steps in the stimulation of anti-cancer activity of nanoparticles. In the first step, the targeted delivery occurs, and in step 2, they are localized in lysosomes through endocytosis. At steps 3 and 4, the pH-degradation occurs and the nanoparticles release the drug in cancer therapy. GC: glycyrrhetinic acid-conjugated chitosan. Reprinted with permission from Ref. [81].

In the previous years, nMOFs have arisen as potential multifunctional nanocarriers for drug delivery in oncological therapy. Nonetheless, there remain a limited number of systems that proficiently operate as targeted drug delivery platforms. This study details the strategic design and synthesis of DOX-loaded nanoscale Zr(IV)-based nMOFs (NH_2_-UiO-66), functionalized with tumor-targeting ligands including folic acid (FA), lactobionic acid (LA), glycyrrhetinic acid (GA), and a combination of LA and GA, to function as effective multifunctional drug delivery systems (DDSs) for HCC treatment. The effective surface modification was comprehensively validated by diverse structural, thermal, and microscopic characterisation methods. Biocompatibility evaluations indicated that the unmodified NH_2_-UiO-66 was non-toxic to HSF cells, however the DOX-loaded dual-ligand nMOF demonstrated increased cytotoxicity towards HepG2 cells. Moreover, fluorescent microscopy revealed enhanced cellular uptake of the dual-ligated nMOF in comparison to the non-ligated and mono-ligated variants. The dual-ligand system exhibited pH-sensitive release of DOX, rendering it a viable option for targeted cancer treatment [80]. Further refining targeted delivery, a MOF system of iron carboxylate coated with a glycyrrhetinic acid-chitosan conjugate showcased enhanced uptake, pH sensitivity, and cytotoxicity in advanced HCC studies. Stimulus-responsive nanomaterials have obtained considerable interest in the development of controlled drug delivery systems owing to their capacity to react to differences between the TME and normal tissues. Iron (III) carboxylate MOF nanoparticles, coated with a glycyrrhetinic acid–chitosan conjugate (MIL-101/GA-CS), were effectively engineered as a pH-responsive and target-selective vehicle for the delivery of DOX in the treatment of HCC. These nanocarriers exhibit homogeneous particle size, a high drug loading capacity of 28.89%, and an efficient pH-dependent drug release profile, with DOX release rates of 2.74% at pH 7.4 and 89.18% at pH 5.5 over a duration of 72 h. *In vitro* cytotoxicity evaluations shown that the DOX-loaded nanocarriers markedly suppressed HepG2 cell proliferation by continuous DOX release, while displaying no intrinsic toxicity. Cellular uptake investigations validated the targeted administration to HepG2 cells by glycyrrhetinic acid receptor-mediated endocytosis. Furthermore, research utilizing *in vitro* 3D hepatoma cell spheroids demonstrated significant penetration and tumoricidal effects. The results indicate that MIL-101-DOX/GA-CS nanoparticles possess significant promise as a pH-responsive drug delivery system for cancer treatment (Fig. 7) [81].

**Fig. 8:**
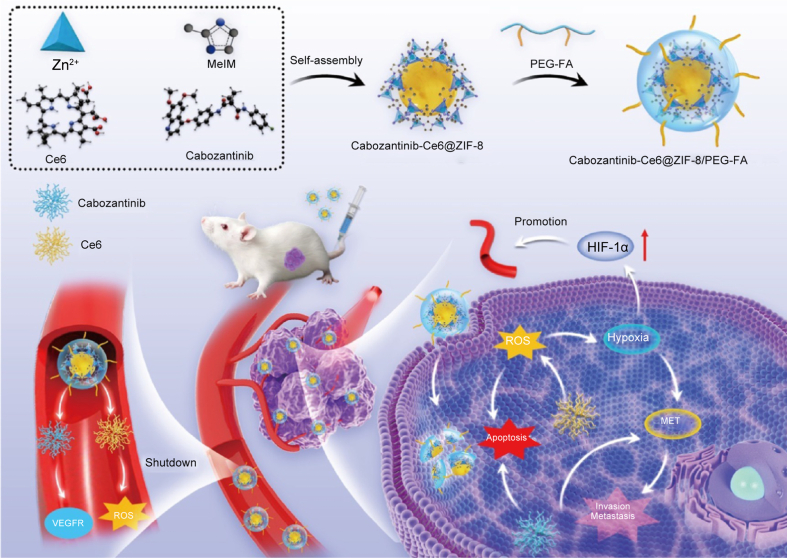
Development of CAB/Ce6@ZIF-8@PEG-FA (CCZP) nanoparticles for anti-hepatocellular carcinoma (HCC) management and suppression of metastatic mechanisms. The self-assembled nanoparticles can deliver cabozantinib and chlorin e6 (Ce6). These nanostructures enhance apoptosis and decrease invasion and metastasis. CAB: cabozantinib; MeIM: 2-methylimidazole; ZIF-8: zeolitic imidazolate framework-8; PEG-FA: polyethylene glycol-folic acid; ROS: reactive oxygen species; VEGFR: vascular endothelial growth factor receptor; HIF-1α: hypoxia-inducible factor 1-alpha; MET: mesenchymal-epithelial transition. Reprinted with permission from Ref. [82].

Multimodal treatment is an essential approach for surmounting tumor medication resistance. PDT produces ROS that can directly eradicate tumor cells, providing advantages such as reusability and a low possibility of drug resistance. Nonetheless, PDT depletes therapeutic oxygen and impairs tumor microvessels, resulting in hypoxia inside tumor tissues. The hypoxic environment induces the upregulation of receptor tyrosine kinase (c-MET) and vascular endothelial growth factor receptor (VEGFR), hence increasing tumor invasiveness and the risk of metastasis. Cabozantinib (CAB), a targeted molecular agent, inhibits many targets, including c-MET and VEGFR, successfully mitigating the growth of HCC. This study presents the development of a pH-responsive nanoparticle, CAB/Ce6@ZIF-8@PEG-FA (CCZP), co-encapsulating CAB and chlorin e6 (Ce6). Under laser irradiation, CCZP provides a synergistic therapeutic effect via both PDT and targeted molecular therapy. CAB effectively mitigates the PDT-induced upregulation of MET and VEGFR, thereby diminishing tumor cell invasiveness and metastasis (Fig. 8) [82]. In the last years, multifunctional nMOFs have experienced considerable progress in the improvement of DDSs. Nonetheless, obstacles persist, especially in attaining accurate and selective cellular targeting and facilitating the fast release of drugs adsorbed on or within nanocarriers. These constraints impede its wider utilization in drug delivery. To address these issues, a biocompatible zirconium-based nMOF was engineered with a customized core and a shell made of polyethyleneimine (PEI) conjugated with the hepatic tumor-targeting ligand glycyrrhetinic acid. This optimized core–shell architecture serves as a sophisticated nanoplatform for the regulated and targeted administration of the anticancer agent Dox to hepatic cancer cells (HepG2). The resultant nanostructure, Dox@nMOF-PEI-GA, exhibited a substantial drug loading capacity of 23%, an acidic pH-responsive release mechanism, and prolonged drug release over nine days. Moreover, it improved selectivity for neoplastic cells. The unloaded nanostructures demonstrated negligible cytotoxicity towards both normal human skin fibroblasts (HSF) and HepG2 cells, whereas the Dox-loaded variants exhibited a markedly increased cytotoxic effect against hepatic tumor cells, underscoring their potential for active drug delivery and efficacious cancer treatment [83]. To address solubility challenges, a cyclodextrin-based MOF was developed to boost the bioavailability and therapeutic potential of triptolide (TPL) in HCC.

TPL has been employed in the management of HCC; however, its restricted aqueous solubility presents a difficulty for practical use. A cyclodextrin-based MOF loaded with TPL (TPL@CD-MOF) was developed to augment the solubility and bioavailability of TPL, consequently enhancing its anti-tumor efficacy against HCC. The Brunauer–Emmett–Teller (BET) surface area and pore size of TPL@CD-MOF were determined to be 10.4 m^2^/g and 1.1 nm, respectively. XRD research validated the incorporation of TPL into the CD-MOF framework. *In vitro* release tests demonstrated that TPL@CD-MOF displayed a more gradual release profile than free TPL. The CD-MOF formulation also enhanced bioavailability. TPL@CD-MOF produced marginally superior, albeit statistically significant, anti-tumor effects relative to free TPL. This little improvement in therapeutic effectiveness is probably attributable to enhanced absorption *in vivo* [84]. Expanding on immune-modulatory strategies, a dual-action MOF nanoparticle combining cytosine–phosphate–guanine oligodeoxynucleotides (CpG ODNs) and 5,6-dimethylxanthenone-4-acetic acid (DMXAA) was engineered to reshape the TME and amplify antitumor immunity. Immunotherapy has shown remarkable efficacy in clinical applications; nevertheless, addressing HCC, characterized by its high vascularization, presents a considerable difficulty. Recent studies reveal that MOF-801 functions as a STING through Toll-like receptor 4 (TLR4), in addition to its role as a drug delivery platform. A self-assembled nanoparticle, MOF-CpG-DMXAA, is constituted by the coordination of CpG ODNs with the STING agonist DMXAA, which furthermore exhibits vascular disruptive properties. This nanoparticle efficiently transports both CpG ODNs and DMXAA to target cells, synergistically improving the TME by reprogramming tumor-associated macrophages (TAMs), facilitating the maturation of dendritic cells (DCs), and disrupting tumor vasculature. In animal models with HCC, MOF-CpG-DMXAA stimulates systemic immune activation and generates robust tumoricidal immune responses, resulting in improved immunotherapeutic effectiveness in both orthotopic and recurrent HCC [85]. Additionally, a ferroptosis-inducing iron-based MOF nanomedicine was designed to combine ablation enhancement and tumor suppression, integrating imaging and therapeutic functionalities. PFP-Apa-MOF, a nanoplatform integrating perfluoropentane (PFP), apatinib (Apa), and an iron-based MOF, was synthesized and described for the improved treatment of HCC. Spherical shape, advantageous size distribution (∼386.6 nm), excellent stability, and pH/MWA-responsive Apa release were noted. Peroxidase-like activity was validated, and significant cytotoxicity in HepG2 cells was elicited, especially when coupled with microwave ablation (MWA), resulting in increased ROS production, apoptosis, and lipid peroxidation. *In vivo* tumor accumulation was accomplished, and tumor development was significantly suppressed by the combination of PFP-Apa-MOF and MWA. Apoptosis (assessed using terminal deoxynucleotidyl transferase dUTP nick end labeling (TUNEL) staining) and ferroptosis (indicated by decreased GPX4 expression) were augmented. Ultrasound imaging demonstrated significant contrast enhancement following microwave ablation due to temperature-induced bubble formation. No significant systemic toxicity was observed, affirming safety and the possibility for integrated therapeutic and diagnostic (theranostic) applications [86]. To further intensify multimodal treatment, an advanced iron-based MOF platform was created, integrating chemotherapy, PDT, and chemodynamic therapy for synergistic cancer cell eradication. The transition from monotherapy to multimodal combination medicines designed to optimize synergistic effects is becoming prevalent in cancer treatment. This study presents a unique multifunctional nanodrug delivery system utilizing iron-based MOFs. The MIL-101(Fe) system is infused with Dox, dihydroartemisinin (DHA), and glucose oxidase (GOx), and is additionally enhanced by the covalent attachment of the photosensitizer 5,10,15,20-tetrakis(4-carboxyphenyl)porphyrin (TCPP). The whole structure is enveloped in polydopamine (PDA), yielding the composite MIL-101(Fe)-DOX-DHA@TCPP/GOx@PDA, referred to as MDDTG@P. Upon entering the TME, MDDTG@P catalyzes the conversion of hydrogen peroxide (H_2_O_2_) into highly reactive hydroxyl radicals (·OH) while concurrently depleting intracellular glutathione (GSH), thereby causing chemodynamic treatment (CDT). Furthermore, the Fe^2+^ ions released from the system activate DHA, augmenting the CDT impact and facilitating tumor cell apoptosis. The incorporated GOx utilizes glucose and oxygen, exacerbating starvation and producing more H_2_O_2_ for the Fenton reaction this dual mechanism results in both starvation treatment and CDT. Furthermore, the covalently bonded TCPP bestows robust PDT efficacy to the system, facilitating light-activated tumor eradication [87]. To complement these innovations, another study employed MIL-100-based nanoparticles for oxaliplatin delivery, merging chemotherapy with ROS generation and photothermal therapy for enhanced precision.

**Fig. 9:**
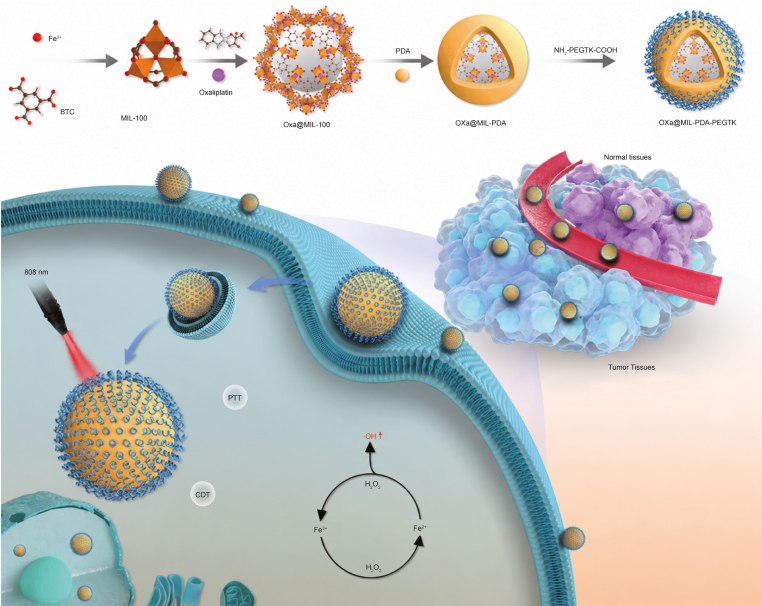
A visual representation of the manufacturing process and therapeutic function of oxaliplatin-loaded metal-organic framework MIL-100 coated with polydopamineand modified with PEGTK (Oxa@MIL-PDA-PEGTK) nanoparticles. The accumulation of nanoparticles mainly occurs in tumor tissue and after internalization, they cause photothermal therapy (PTT) upon exposure to 808 nm irradiation. BTC: 1,3,5-benzenetricarboxylic acid; NH_2_-PEGTK-COOH: polyethylene glycol–thioketal–amine; CDT: chemodynamic therapy. Reprinted with permission from Ref. [88].

The activation of nanoparticles inside the TME improves the therapeutic effectiveness of chemotherapy in several cancer types. A study developed a biocompatible nanoparticle system utilizing MIL-100 nanoparticles for the delivery of oxaliplatin in the treatment of HCC. The nanoparticles were coated with PDA and NH2-PEGTK-COOH, subsequently loaded with oxaliplatin to create the multifunctional system Oxa@MIL-PDA-PEGTK. In the TME, this mechanism is activated, resulting in the production of cytotoxic ROS via the Fenton reaction and the release of oxaliplatin. Moreover, upon exposure to NIR irradiation, the nanoparticles generate localized hyperthermia at the tumor location. The light-induced activation of Oxa@MIL-PDA-PEGTK enhances drug transport efficiency, facilitates targeted activation, and diminishes off-target toxicity, as validated by *in vitro* and *in vivo* studies (Fig. 9) [88]. Further, a novel zinc-doped cerium oxide nanocomposite derived from a MOF precursor was developed, offering selective cytotoxicity towards liver cancer cells. Zinc-doped cerium oxide nanocomposites (ZnO/CeO_2_ NCs) were designed by the combustion technique utilizing a MOF precursor. There was effective synthesis of ZnO/CeO_2_ nanocomposites, indicating a crystallite size of 31.9 nm. Field emission scanning electron microscopy (FESEM) and TEM imaging revealed both hexagonal and spherical morphologies, with the solid-phase particle size quantified at 65.03 ± 30.86 nm. Observations using DLS, TEM, and FESEM indicated a pronounced propensity for agglomeration or aggregation in both aqueous solutions and solid-state forms. The anticancer efficacy of ZnO/CeO_2_ nanocomposites was assessed against HepG2 liver cancer cells, demonstrating a concentration-dependent reduction in cell growth. The cytotoxicity evaluation with NIH-3T3 fibroblast cells revealed reduced toxicity relative to HepG2 cancer cells [89].

In a strategy blending vascular modulation with immunotherapy, a biomimetic MOF coated with tumor cell membranes was engineered to co-deliver synergistic agents for HCC treatment. The restricted quantity and inadequate functionality of tumour-infiltrating lymphocytes (TILs) significantly impede the efficacy of PD-1 inhibitors in the treatment of HCC. A combination of tanshinone IIA (TSA) and astragaloside IV (As) is employed to augment both the amount and functionality of tumor-infiltrating lymphocytes (TILs) by regulating tumor vasculature and reducing levels of immunosuppressive factors, respectively. A magnetic MOF nanoparticle, coated with a homologous tumor cell membrane (Hm) and designated as Hm@TSA/As-MOF, is engineered to enhance PD-1 antibody treatment by facilitating the co-delivery of TSA and As into the HCC microenvironment. This spherical nanoparticle has a substantial total drug loading of 16.13 wt%. The Hm coating and magnetic characteristics of Hm@TSA/As-MOF provide dual targeting homologous and magnetic, facilitating evasion of interference from tumor cells in ascites and enabling precise targeting of solid tumors. The Hm coating facilitates the evasion of nanoparticles from clearance by macrophages. Upon entering the HCC microenvironment, the release of TSA and As is initiated, consequently augmenting TIL presence and activity, and amplifying the anti-tumor effects of the PD-1 antibody through synergistic interaction [90]. Finally, metal ions have obtained considerable interest in anticancer treatment owing to their unique modes of action. The investigation of several metal-induced cell death pathways for synergistic antitumor effects is constrained, mainly due to the intricacies of developing metal-based multimodal treatment platforms. A variety of glutathione-responsive CaCu-based MOFs have been designed to include Dox and ovalbumin (OVA) utilizing a "all-in-one" technique. These MOFs are further modified with galactosamine-conjugated HA, creating a multifunctional therapeutic system designated SCC/Dox@OVA-HG, which facilitates targeted distribution and amplifies synergistic antitumor efficacy. Upon arrival to the TME, SCC/Dox@OVA-HG rapidly degrades in reaction to increased glutathione levels, resulting in the regulated release of its therapeutic constituents. The released Cu^+^ ions promote the transformation of hydrogen peroxide into extremely harmful ROS, hence enhancing CDT. Simultaneously, the release of Ca^2+^ ions disturbs intracellular calcium equilibrium, facilitating calcium overload treatment and exacerbating metal-induced cellular death processes. The combination of OVA with Ca^2+^/Cu^2+^ promotes macrophage polarization towards the M1 phenotype, facilitating immune-mediated tumor eradication. Dox, delivered concurrently, intercalates into DNA to provide its chemotherapeutic action. SCC/Dox@OVA-HG exhibits robust anticancer efficacy via a synergistic strategy that amalgamates chemotherapy, CDT, calcium overload, and immunotherapy [91].

#### 2.3.4 GC

Naringenin (NAR), a molecule characterized by low water solubility, was utilized as a model substance and integrated into porous MOFs to develop an inclusion complex designated as NAR@ZIF-8. The compound was encapsulated in liposomes via the film dispersion process to design an advanced drug delivery system. A thorough formulation procedure was conducted, optimizing critical parameters including the lipid-to-drug ratio, phospholipid-to-cholesterol ratio, and hydration temperature, utilizing a Box-Behnken design informed by initial single-factor trials. The resultant liposomal formulation had a spherical or nearly spherical shape, characterized by a homogeneous particle size distribution and preserved structural integrity. Characterization indicated an average particle diameter of 113.2 ± 1.4 nm and a zeta potential of −7.536 ± 0.264 mV. *In vitro* release tests were performed to assess the drug release patterns of free NAR, NAR@ZIF-8, and NAR@ZIF-8-loaded liposomes. Although both free NAR and NAR@ZIF-8 exhibited fast release profiles, the liposomal NAR@ZIF-8 system had a more regulated and prolonged release, attaining 79.86% cumulative release over 72 h. Cytotoxicity studies conducted on lung adenocarcinoma A549 cells and GC SGC-7901 cells demonstrated that NAR@ZIF-8 liposomes exhibited much more inhibitory effects than free NAR and NAR@ZIF-8. The increased cytotoxicity was demonstrated to be concentration-dependent, underscoring the promise of this liposomal system for enhanced anticancer drug delivery [92]. Shifting focus toward diagnostic innovations, an advanced electrochemiluminescence (ECL) biosensor integrating MOF nanosheets and Cu nanoclusters was developed for ultrasensitive detection of GC-related miRNA. An innovative ECL biosensor has been developed for the identification of miRNA in extracellular vesicles (EVs) associated with GC. The procedure started with the effective *in situ* synthesis of copper nanoclusters (Cu NCs) on MOF nanosheets. The confinement effect inside the porous structure of Zn-based MOFs resulted in Cu NCs with high quantum yield and exceptional stability. Furthermore, the Zn MOFs exhibited advantageous electrochemical activity, augmenting the ECL response. The nanosized MOFs performed multiple roles: serving as a sensing platform for Cu NC loading, enabling biomolecule attachment, and reducing steric hindrance to enhance biomolecular recognition. Additionally, the electrode surface was altered using AuNPs/MXene and a phospholipid layer, resulting in better electron transport and higher target identification efficiency. This Cu NCs/Zn MOF nanosheet-based ECL sensor facilitated the sensitive detection of miRNA-421 within a concentration range of 1 fM to 1 nM, attaining a detection limit as low as 0.5 fM. EVs obtained from the clinical ascitic fluid of GC patients were effectively examined [93]. In a complementary approach, a highly efficient MOF-based adsorbent system was engineered for precise quantification of sugar phosphates (SPx) in clinical GC samples. SPx, essential in metabolic activities, are challenging to identify in biological samples due to their instability, structural resemblance, and low prevalence. To tackle this issue, UiO-66-NH_2_ and ZrO_2_ coated SiO_2_ (SBA-15) core-shell adsorbents (UiO-66-NH_2_@SBA-15 and ZrO_2_@SBA-15) were developed for dispersive solid-phase extraction, subsequently employing gas chromatography-mass spectrometry (GC-MS) analysis. Optimization by univariate and response surface methodologies shown that the materials exhibited significant adsorption capacity and selectivity for SPx. The approach exhibited remarkable linearity (5.0–5000.0 ng/mL), minimal detection and quantification thresholds (0.001 ng/mL and 0.005 ng/mL, respectively), and superior accuracy (RSD <14.7%). Recovery rates ranging from 89.1% to 113.8% were attained in serum, saliva, and cell samples, with UiO-66-NH_2_@SBA-15 demonstrating particular efficacy in assessing SPx in stomach cancer patient samples owing to its enhanced adsorption capability. This methodology provides a valuable instrument for investigating SPx metabolism and elucidating illness processes [94]. To address *H. pylori*-related gastric pathologies, a hydrogen-generating MOF nanoparticle encapsulated in an ammonium persulfate (AP) hydrogel was created to offer targeted antibacterial and mucosal repair effects. *H. pylori* infection is a primary factor in chronic gastritis, peptic ulcers, and gastric carcinoma. Traditional antibiotic therapies exhibit a lack of specificity, frequently resulting in considerable bacterial resistance and disturbance of gut flora. Moreover, antibiotics are predominantly ineffective in mitigating the pronounced inflammatory response and the compromised stomach mucosal barrier linked to *H. pylori* infection. A unique therapeutic strategy employs a pH-responsive MOF hydrogen-generating nanoparticle, Pd(H)@ZIF-8, enclosed in an ascorbate palmitate hydrogel. *In vitro* and *in vivo* investigations demonstrate that the AP hydrogel may preferentially target and attach to inflamed regions through electrostatic interactions, subsequently degrading via MMPs abundant in inflamed tissue. Upon release, the Pd(H)@ZIF-8 nanoparticles breakdown in stomach acid, liberating zinc ions (Zn^2+^) and hydrogen, which collaboratively eradicate *H. pylori*, diminish inflammation, and facilitate the restoration of the gastric mucosa. This hydrogen-generating MOF system (Pd(H)@ZIF-8@AP) effectively preserves intestinal microbiota equilibrium, providing a targeted, efficient, and microbiota-compatible therapeutic approach for *H. pylori* infection [95]. For rapid exosome detection, an iron-based MOF with aptamer modifications was established, enabling a simple and fast visual colorimetric assay suitable for clinical applications. A quick, economical, and uncomplicated technique for exosome detection was developed by altering the surface of an iron-based metal-organic framework (Fe-MOF) with designed DNA ligands. CD63-specific aptamers, targeting the exosomal transmembrane protein CD63, functioned as both an optically sensitive layer and a selective recognition element, facilitating the creation of a bio-interface on Fe-MOF that effectively regulates its catalytic activity towards a chromogenic substrate. The self-assembly of CD63 aptamers on Fe-MOF markedly improved its intrinsic peroxidase-like catalytic characteristics. Specific binding of exosomes to CD63 aptamers induced conformational changes in the DNA ligands, which modified the catalytic activity of Fe-MOF, resulting in a discernible color shift that enabled visual identification of exosomes. The one-step "mixing-and-detection" method demonstrated superior efficacy in measuring exosomes from human breast cancer cell lines throughout a concentration spectrum of 1.1 × 10^5^ to 2.2 × 10^7^ particles/μL, with a detection threshold of 5.2 × 10^4^ particles/μL. Moreover, it facilitated the distinction of exosomal CD63 protein expression across breast, stomach, and lung cancer cell lines in 17 minmin. The successful identification of exosomes in serum samples further demonstrates the method's potential as a practical tool for quick, easy, and cost-effective clinical exosome testing [96]. To enhance drug delivery, zinc-based MOFs were used to encapsulate hydrophobic CCM, improving solubility and cytotoxicity against GC cells. Two nanoporous Zn-based MOFs with low water stability, one containing a NO_2_ functional group and the other devoid of it, were produced by the reflux approach and used for the encapsulation of curcumin (CCM). The Zn-based MOFs (DMOF-1 and DMOF-1-NO_2_) were studied by FT-IR, PXRD, ^1^H NMR, N_2_ adsorption, SEM, UV–vis, and fluorescence microscopy techniques. The drug loading capabilities were established at 22.4 wt% for DMOF-1 and 28.3 wt% for DMOF-1-NO_2_. Computational models corroborated the drug loading results. These MOFs, owing to their restricted hydrolytic stability, efficiently tackled the problem of curcumin's poor water solubility, a prevalent concern with hydrophobic anticancer agents. The findings indicated that these hydrolytically unstable MOFs improved the solubility of CCM and its cytotoxic effects on AGS cancer cells relative to free CCM [97]. To expand molecular diagnostics, a suite of MOF-based sensing platforms was designed for sensitive and specific detection of multiple GC-associated miRNAs. Five unique sensing platforms have been developed for the identification of gastric cancer-associated miRNAs. These platforms consist of hybrid materials formed from a water-stable MOF, integrated with five carboxyfluorescein (FAM)-labeled single-stranded DNA probes (P-DNA), each targeting a distinct miRNA. In the hybrid structure, MOF 1 engages intimately with the P-DNA through electrostatic and/or π-stacking interactions, leading to fluorescence quenching of the FAM label via a photo-induced electron transfer (PET) process. Upon the introduction of the corresponding target miRNAs, miR-185, miR-20a, miR-92b, miR-25, and miR-210, which are aberrantly expressed in the plasma of GC patients, the P-DNA is released from the MOF surface due to enhanced hybridization with the target miRNA. This displacement reinstates the FAM fluorescence signal. Each P-DNA@1 hybrid has increased sensitivity and specificity for its corresponding target miRNA, with detection thresholds between 91 and 559 pM, and displays no cross-reactivity with the other four miRNA sequences [98].

#### 2.3.5 EC

Although there have been significant advancements in developing MOF-based nanoplatforms for cancer treatment, there are still numerous essential challenges before complete clinical application can be achieved. A key issue is the complicated TME, which differs significantly among cancer types and can affect the effectiveness of MOFs in treatment. For example, although pH-responsiveness, redox sensitivity, and activation triggered by hydrogen sulfide have demonstrated potential in colorectal and liver cancers, the variability of TMEs might lead to unpredictable degradation rates, off-target effects, or incomplete drug release. Moreover, although multifunctionality (integrating chemotherapy, photodynamic therapy, chemodynamic therapy, and immunotherapy) brings synergistic advantages, it also heightens the risk of unexpected interactions, nanoparticle clustering, and excessive activation of the immune system. Attaining accurate regulation of cargo release dynamics, guaranteeing extensive tumor infiltration, and preventing early elimination or degradation by the immune system continue to be significant technical challenges. A notable challenge is found in maintaining a balance between effectiveness, safety, and scalability. Even though several MOF systems, such as UiO-66-NH_2_(Hf), Fe-TBP, and Cu-based nanozymes, have shown significant anticancer properties and biosafety in preclinical studies, further studies need to be carried out on long-term toxicity, biodegradability, and clearance mechanisms. Certain MOFs incorporate heavy metals (such as hafnium and thorium), which raises concerns regarding possible accumulation and toxicity, particularly with frequent or high-dose therapies. Furthermore, the consistent large-scale production of MOFs with uniform size, shape, and functionalization, while preserving therapeutic effectiveness and adhering to regulatory standards, presents a significant challenge.

### 2.4 IONPs

IONPs are a significant asset in managing GI tumors because of their distinct magnetic characteristics, compatibility with biological systems, and multifunctional abilities. Their superparamagnetic properties enable accurate targeting with external magnetic fields, improving localized drug delivery and reducing systemic toxicity. IONPs can be loaded with chemotherapy drugs or nucleic acids (siRNA) to specifically transport therapeutic substances to tumor locations, enhancing treatment effectiveness and minimizing off-target effects. Moreover, their capacity to produce heat within alternating magnetic fields allows for magnetic hyperthermia, a non-invasive method that specifically elevates tumor temperatures to trigger cancer cell death while protecting healthy tissue. IONPs additionally function as outstanding contrast agents in MRI and aid in early tumor identification, precise staging, and real-time assessment of treatment response. Additionally, their surface may be modified with targeting ligands (antibodies, peptides) to increase tumor specificity, or with polymers to boost stability and circulation duration. In addition to therapy and imaging, IONPs can alter the TME by affecting immune responses or interfering with cancer-related signaling pathways. Although they offer benefits, it is necessary to resolve issues such as possible long-term toxicity, ideal biodistribution, and scalable production for clinical application. Nonetheless, incorporating IONPs into GI cancer therapy presents a hopeful theranostic platform, merging diagnostic capabilities, targeted treatment, and real-time observation to enhance patient results.

#### 2.4.1 CRC

CRC persists as one of the most prevalent and fatal malignancies, with its advancement and chemoresistance frequently affected by *Fusobacterium nucleatum* (Fn), a bacterium that promotes tumor proliferation and diminishes therapeutic efficacy. Conventional antibiotics are ineffective in eliminating Fn at tumor locations because of variables such as biofilm development, and chemotherapy alone is unable to impede tumor growth. Consequently, innnovative techniques are required to eradicate Fn and improve antitumor effectiveness for superior CRC therapy results. A nanodrug (OPPL) was developed to tackle this issue, consisting of oleic acid-modified superparamagnetic iron oxide nanoparticles (O-SPIONs) and an amphiphilic polymer (PPL), which co-delivers a platinum-based prodrug and the antibacterial agent lauric acid (LA). This formulation enhances antibacterial efficacy and destroys Fn biofilms, with the LA and peroxidase-like activity of OPPL producing ROS. Furthermore, OPPL increases intracellular ROS levels, causes lipid peroxidation, and depletes glutathione, therefore initiating ferroptosis in CRC cells. OPPL exhibits significant cytotoxicity against CRC due to the synergistic effects of chemotherapy and ferroptosis activation. *In vivo* findings demonstrate that OPPL increases tumor accumulation, facilitates magnetic resonance imaging, limits tumor development, and reduces intratumoral Fn populations [99]. In addition to this, the application of nanoplatforms for improving sensitivity of cancer cells to therapy is of importance. A nanoplatform based on iron oxide clusters was developed to tackle the pressing clinical issue of resistance to tumor necrosis factor-related apoptosis-inducing ligand 2 (TRAIL/Apo2L), frequently caused by inadequate levels or mutations of TRAIL receptors. The method, known as NanoTRAIL (TRAIL/Apo2L-IONPs), was developed to improve treatment effectiveness and facilitate MR image-guided evaluation in CRC, a malignancy that often has inadequate response to TRAIL/Apo2L and standard chemotherapy. NanoTRAIL facilitates the activation of the JNK (c-Jun N-terminal kinase) pathway through ROS, resulting in autophagy-mediated overexpression of DR5, which substantially amplifies the anticancer efficacy of TRAIL/Apo2L. This effect was confirmed in CRC cell lines exhibiting different sensitivities to TRAIL: HT-29 (resistant), SW-480 (intermediately resistant), and HCT-116 (sensitive). In a subcutaneous CRC murine model, MR T2-weighted contrast imaging revealed effective *in vivo* retention of NanoTRAIL, and treatment significantly inhibited tumor development and prolonged life without discernible adverse effects compared to control or TRAIL/Apo2L monotherapy. Furthermore, in patient-derived xenograft models of CRC, NanoTRAIL therapy markedly enhanced survival outcomes, correlated with sustained ROS-dependent autophagy-mediated DR5 overexpression and heightened tumor apoptosis [100]. Further improvement can be provided through the introduction of combination therapies. Strategies for tumor therapy utilizing nanoparticles have been thoroughly investigated; nevertheless, their therapeutic efficacy is frequently impeded by inadequate nanoparticle accumulation in tumor tissues and the limited antitumor effects of individual treatment regimens. A tumor-targeting biomimetic sonosensitizer-conjugated iron oxide (Fe_3_O_4_) nanocatalyst system was created to provide a combination CDT and SDT for the treatment of CRC. Bovine serum albumin (BSA)-modified Fe_3_O_4_ nanoparticles were produced using a simple co-precipitation approach and subsequently coupled with chlorin e6 (Ce6) as the sonosensitizer. The nanoparticles were further coated with a CT26 cancer cell membrane to develop biomimetic nanocatalysts (MBFC) with tumor-targeting properties. The MBFC nanocatalysts exhibited significant catalytic activity and effective sonodynamic characteristics, generating a considerable quantity of ROS under ultrasonic (US) stimulation in the TME. Cellular uptake investigations demonstrated a significant internalization efficiency of MBFC, ascribed to the homologous targeting mechanism conferred by the cancer cell membrane coating. The MBFC nanocatalysts enabled a synergistic impact of CDT and SDT, markedly enhancing apoptosis in CT26 cells *in vitro* and efficiently inhibiting CT26 tumor development *in vivo* [101].

#### 2.4.2 HCC

Nanoparticles are widely utilized in biological research and oncological therapy. In the realm of HCC, several nanoplatforms have been engineered to augment therapeutic efficacy; yet, these strategies remain inadequate for complete tumor eradication. This work presents a new vanadium (V)-doped iron oxide nanoparticle (VIO) that combines chemodynamic treatment, photothermal effects, and diagnostic functionalities into a single multifunctional agent to enhance tumor suppression. *In vitro* studies revealed that VIO-based nanoagents significantly suppressed the growth of HCC. VIO increases ROS levels, inducing both apoptosis and ferroptosis, resulting in cell death. Significantly, VIO attacks both cancerous cells and endothelial cells. In addition to causing cell death, VIO impedes tube formation and cell migration in human umbilical vein endothelial cells (HUVECs) and C166 cell models, indicating its antiangiogenic characteristics. VIO inhibits tumor development and enhances apoptosis in tumor tissues. Moreover, the treated groups demonstrated significant regression of blood vessels and an increase in necrotic areas. Notably, when utilized in conjunction with photothermal treatment, VIO entirely eliminated malignant tissue. This VIO nanoplatform has significant potential as a multifunctional anticancer nanodrug [102].

A new polymeric nanoparticle drug delivery system known as YCC-DOX, comprising poly(ethylene oxide)-trimellitic anhydride chloride-folate (PEO-TMA-FA), DOX, superparamagnetic iron oxide (Fe_3_O_4_), and folate was developed to augment anticancer activity and mitigate adverse effects. In liver cancer models including rats and rabbits, YCC-DOX demonstrated superior efficacy compared to free DOX (FD) and the commercial liposomal formulation DOXIL®, significantly decreasing tumor volume and selectively targeting tumors that express folate receptors. YCC-DOX demonstrated enhanced MRI sensitivity relative to Resovist®, albeit its reduced iron concentration. Immunohistochemical study demonstrated reduced expression of angiogenesis (CD34) and proliferation (Ki-67) markers, along with enhanced apoptosis (TUNEL test) in tumors subjected to YCC-DOX treatment [103]. Hydrophilic, surface-functionalized SPIOs were engineered for magnetic fluid hyperthermia (MFH) therapy of hepatic carcinoma. The SPIOs were produced by co-precipitation and thermolysis techniques and subsequently functionalized with short-chain molecules, including 1,4-diaminobenzene (14DAB), 4-aminobenzoic acid (4ABA), 3,4-diaminobenzoic acid (34DABA), and their combinations with aromatic acids. Among the diverse formulations, 34DABA-coated SPIOs demonstrated exceptional magnetic properties (Ms = 55–71 emu/g), remarkable water dispersibility, and the highest heating efficiency in magnetic fluid hyperthermia (SAR = 432.1 W·g⁻¹ (Fe), ILP = 5.2 nHm^2^/kg at 0.5 mg/mL), owing to improved π–π conjugation and nanoparticle aggregation. Furthermore, excellent cytocompatibility and elevated cancer cell-killing efficacy (61%–88%) were highlighted in HepG2 liver cancer cells, signifying substantial promise for the application as effective nanomedicines in magnetic fluid hyperthermia-based cancer treatment [104]. Inadequate thermal ablation for HCC may result in remaining tumors that facilitate recurrence by augmenting pro-tumorigenic M2 macrophages. In response, d-mannose-chelated iron oxide nanoparticles (man-IONPs) were developed that specifically target and reprogram M2-like macrophages into anticancer M1 phenotypes. *In vitro* and *in vivo* studies demonstrated that man-IONPs accumulate in peri-ablation zones post-microwave ablation (MWA), successfully transforming the immunosuppressive TME into an immunoactivating milieu. The polarization of macrophages remarkably impeded HCC growth in a murine model, indicating that the integration of MWA with man-IONPs is a feasible approach to eradicate remaining tumors and enhance treatment results [105].

#### 2.4.3 GC

A unique nonviral and MRI-visible nanocarrier known as PEG-g-PEI-SPION, was developed for the delivery of small interfering RNA (siRNA) in the treatment of GC. The carrier was thoroughly evaluated for its dimensions, surface charge, siRNA binding capacity, cytotoxicity, transfection efficacy, cellular uptake, and MRI imaging potential. The PEG-g-PEI-SPION/siRNA combination, targeting CD44 variant isoform 6 (CD44v6), a marker associated with metastasis in gastric cancer, significantly inhibited CD44v6 expression in SGC-7901 cells at an optimum N/P ratio of 10, leading to reduced cell migration and invasion. Additionally, PEG-g-PEI-SPION served as an effective MRI contrast agent *in vivo*, suggesting its potential as a dual-function vector for targeted gene therapy and noninvasive imaging in gastric cancer [106]. MRI augmented with ultrasmall superparamagnetic iron oxide (USPIO) and novel diagnostic criteria was assessed for identifying regional lymph node metastases in GC. Out of 31 patients and 1000 dissected lymph nodes, 519 were evaluable with MRI. The technique obtained 96.2% sensitivity, 92.5% specificity, and 93.3% accuracy utilizing traditional criteria grounded in T2-weighted signal intensity patterns. The revised criteria, which further examined ambiguous nodes by T1-weighted imaging to detect adipose tissue, enhanced specificity and overall accuracy to 98.3% and 97.1%, respectively, while preserving excellent sensitivity and negative predictive value [107]. A multifunctional, targeted siRNA delivery system utilizing folic acid (FA) and disulfide-PEG-conjugated PEI complexed with SPIONs was created for PD-L1 knockdown in GC. The FA-PEG-SS-PEI-SPION/siRNA polyplexes exhibited effective siRNA binding, minimal cytotoxicity, improved transfection, and targeted cellular uptake in folate receptor-overexpressing SGC-7901 GC cells. At a nitrogen:phosphate (N:P) ratio of 10, the polyplexes successfully inhibited PD-L1 expression, functioned as T2-weighted MRI contrast agents, and influenced T-cell cytokine responses in a coculture model, underscoring their potential as a theranostic platform for targeted GC immunotherapy and imaging [108]. Another experiment examines the targeting of autophagy, the cell's internal recycling mechanism, to overcome hyperthermia resistance, a significant obstacle in cancer treatment, specifically in GC. The magnetic iron-oxide nanoparticles coated with artemisinin (ART-MNPs) were developed to augment autophagy and thus increase the susceptibility of cancer cells to hyperthermia. The ART-MNPs exhibited significant magnetic responsiveness and successfully increased the ambient temperature by 7 °C at a frequency of 580.3 kHz. They demonstrated substantial cytotoxicity against human GC AGS cells, with an IC50 of 1.9 μg/mL, markedly lower than that of MNPs (9.7 μg/mL) and artemisinin alone (9.4 μg/mL). The combination index study validated a synergistic interaction among the components. Subsequent mechanistic investigations revealed that ART-MNPs significantly augmented autophagy activity, 13.58-fold greater than artemisinin and 15.08-fold greater than MNPs alone, suggesting that this substantial autophagy enhancement plays a crucial role in surmounting hyperthermia resistance and more effectively diminishing cell viability under thermal stress conditions [109]. Lactoferrin-iron oxide nanoparticles (LF-IONPs) were examined for their efficacy in the treatment of GC. The spherical nanoparticles, averaging 5 ± 2 nm in size, demonstrated persistent connections between the iron oxide core and the lactoferrin protein. Research on hyperthermia revealed a temperature elevation of 7 °C at 242.4 kHz, signifying its appropriateness for thermal treatment. In experiments with AGS GC cells, LF-IONPs (10 μg/ml) markedly increased cytotoxicity, resulting in around 68% cell mortality upon heat, and strongly impeded cell migration, particularly after 36 h, underscoring their promise for targeted gastric cancer therapy [110].

#### 2.4.4 PC

A very sensitive nanoprobe, HA-Fe3O4 nanoparticles, has been engineered to augment the early and precise detection of PC by enhancing the accumulation of contrast agents at tumor locations. These multifunctional nanoparticles are designed with HA to specifically target CD44 receptors, which are overexpressed on the membranes of PC cells. The synthesis entails the formation of polyethyleneimine-stabilized Fe_3_O_4_ nanoparticles, then modified with fluorescein isothiocyanate (FITC), polyethylene glycol (mPEG-COOH), and HA. The HA-Fe_3_O_4_ nanoparticles, measuring around 11.9 nm and exhibiting a high r2 relaxivity of 321.4 mM^−1^s^−1^, display exceptional efficacy as T2-weighted MRI contrast agents. *In vitro* studies demonstrate minimal cytotoxicity at concentrations up to 100 μg/mL of iron and indicate significantly increased cellular uptake in HA-targeted cells, showing effective HA-mediated targeting. This technique provides a robust foundation for accurate imaging and may be applicable to many biological uses [111]. A new formulation of superparamagnetic iron oxide nanoparticles containing curcumin (SP-CUR) improves the therapeutic effectiveness of GEM in PC. SP-CUR administers bioactive CUR to PC, enhancing GEM absorption by upregulating nucleoside transporter genes and inhibiting ribonucleotide reductase subunits. Mechanistically, SP-CUR alters the TME by inhibiting the sonic hedgehog (SHH) pathway and CXCR4/CXCL12 signaling, hence disrupting tumor-stromal interactions. It additionally targets cancer stem cells by downregulating pluripotency factors (Nanog, Sox2, c-Myc, Oct-4) and restricting tumor sphere formation. *In vivo*, SP-CUR accumulates in the pancreas, amplifies GEM's anti-tumor efficacy, diminishes tumor growth and metastasis, and modifies tumor stiffness and stromal indicators [112].

A drug delivery system responsive to acidity was developed for PC utilizing iron oxide core-shell magnetic mesoporous silica nanoparticles (M-MSNs) infused with Dox and Gox. The DG@NPs system utilizes the tumor's acidic milieu to initiate the depolymerization of the polydopamine (PDA) shell, facilitating targeted drug release and glucose deprivation for a synergistic approach to chemotherapy and starvation treatment. DG@NPs exhibited efficient cellular uptake, pronounced cytotoxicity, and caused apoptosis *in vitro*, while substantially suppressing tumor development and ensuring biosafety *in vivo* [113]. Another study introduces an innovative interventional photothermal-immunotherapy approach for addressing metastatic PC, utilizing a multifunctional, TME-responsive nanoplatform (IMQ@IONs/ICG). The nanoplatform integrates MRI guiding, drug administration, and tumor regulation through the incorporation of imiquimod (IMQ), ICG, and amorphous iron oxide nanoparticles (IONs). It facilitates accurate MRI-guided PTT, enhancing drug infiltration, triggering ICD, and stimulating anticancer immunity. In aggressive Panc02-H7 mice models, the therapy eliminated primary tumors, reduced metastases, enhanced CD^8+^ T cell infiltration, and prolonged longevity without damaging healthy tissue, presenting a therapeutically viable strategy for addressing PC [114].

#### 2.4.5 EC

Magnetic resonance lymphography utilizing submucosal injection of SPIO was employed to elucidate lymphatic drainage channels in patients with thoracic EC. In 24 patients, SPIO was endoscopically administered into the peritumoral submucosal layer, followed by MRI at several time intervals. The approach efficiently illuminated lymph nodes, demonstrating considerable signal attenuation in nodes that received SPIO. Influx to cervical lymph nodes was noted in 64.3% of patients, and to upper abdominal nodes in 92.9%. The results suggest that MR lymphography with SPIO is an effective technique for delineating lymphatic outflow and detecting lymph node involvement in EC [115]. Ferumoxtran-10-enhanced MRI was assessed for its efficacy in identifying lymph node metastases in EC. Sixteen patients got MRI scans prior to and after to the administration of ferumoxtran-10, which is taken up by macrophages in nonmalignant lymph nodes but not in metastatic ones. Lymph nodes were categorized into three enhanced patterns, two of which signify metastasis. Of the 408 resected nodes, 133 were examined, revealing 24 with definite metastases. The imaging technique demonstrated 100% sensitivity, 95.4% specificity, and 96.2% accuracy, signifying that ferumoxtran-10-enhanced MRI is very proficient in differentiating benign from malignant lymph nodes in EC [116].

Despite considerable progress, various obstacles impede the clinical application of IONPs in GI cancer treatment. A significant constraint is obtaining accurate biodistribution and retention at tumor locations while reducing off-target buildup, particularly in reticuloendothelial organs like the liver and spleen. While active targeting methods (such as folic acid, MUC1, and CD44 ligands) improve tumor specificity, the heterogeneity present within tumors and variations in receptor expression can hinder targeting efficacy. Moreover, maintaining uniform particle size, surface charge, and stability is essential for reproducibility and regulatory approval, yet it continues to be challenging to accomplish at a large manufacturing scale. Additionally, although numerous studies indicate outstanding biocompatibility *in vitro* and small animal models, a thorough assessment of long-term biosafety, biodegradability, and possible immunogenicity in humans is necessary. Another key challenge is to balance between multifunctionality, safety, and performance. Systems that combine imaging (MRI), drug administration, and therapeutic activities such as PTT, CDT, or ferroptosis induction add layers of complexity that may complicate pharmacokinetics and toxicity profiles. For example, systems integrating magnetic hyperthermia or photothermal effects with chemotherapy or immunotherapy should prevent overheating or causing excessive oxidative stress, which may adversely affect healthy tissues. Meanwhile, regulatory challenges are further heightened when multifunctional systems feature various active agents (siRNA, chemotherapeutics, or immune modulators). Ultimately, although numerous platforms have demonstrated effectiveness in mouse models, applying these results to human patients, who have more intricate TME and immune responses, necessitates comprehensive clinical trials and adjustments to guarantee dependable and safe operation.

### 2.5 Zinc oxide nanoparticles (ZnO-NPs)

#### 2.5.1 CRC

Biogenic ZnO-NPs, produced from methanol extract of the stem of *Tinospora cordifolia* (ZnO-NPs TM), demonstrate significant anticancer efficacy against CRC. These nanoparticles were characterized using UV–Vis, FTIR, XRD, SEM, and TEM, and were evaluated on HCT-116 and Caco-2 cell lines, demonstrating cytotoxic effects with IC_50_ values of 31.419 ± 0.682 μg/mL and 36.675 ± 0.916 μg/mL, respectively. The anticancer effectiveness was validated by tests demonstrating decreased mitochondrial membrane potential, increased ROS production, and augmented expression of pro-apoptotic genes Bax and P53. Furthermore, *in vivo* investigations corroborated the tumor-suppressive characteristics of the nanoparticles, indicating their potential as innovative anticancer treatments [117]. The limits of chemotherapy, including adverse effects, drug resistance, and metastasis, propel the exploration of alternative cancer therapies, with nanoparticles emerging as a viable option. A study developed ZnO-NPs with ethanolic and methanolic extracts of *Swertia chirayita* leaves. The resultant ZnO nanoparticles were spherical, with average dimensions of 24.67 nm (ethanolic) and 22.95 nm (methanolic). The cytotoxicity experiments (MTT and acridine orange staining) conducted on CRC cells (HCT-116 and Caco-2) and normal cells (HEK-293) demonstrated that ZnO-NPs exhibited preferential toxicity towards cancer cells, evidenced by much lower IC_50_ values relative to control cells. Moreover, qRT-PCR analysis demonstrated elevated expression of the tumor suppressor gene E-cadherin and reduced expression of metastasis and cell cycle-related genes vimentin and CDK-1, suggesting the apoptosis-inducing and anti-cancer potential of ZnO-NPs in CRC [118].

#### 2.5.2 GC

*Morus nigra*-loaded zinc oxide nanoparticles (MN-ZnONPs) were developed, revealing spherical shape, crystalline structure, and diverse functional groups. The anticancer effects of MN-ZnONPs were evaluated against AGS GC cells by assessing cell viability, apoptotic morphology (AO/EtBr staining), MMP, cell cycle arrest, lipid peroxidation (TBARS), antioxidant levels (SOD, GSH, CAT), and ROS generation. MN-ZnONPs induced cancer cell death by enhancing ROS production, decreasing MMP, increasing lipid peroxidation, reducing antioxidant defenses, and causing cell cycle arrest. Apoptosis was further confirmed by altered expression of apoptosis-related genes (Bax, caspase-9, caspase-3, and Bcl-2), demonstrating that MN-ZnONPs effectively trigger apoptosis in GC cells via multiple cellular and molecular mechanisms [119]. Meanwhile, a study introduces an eco-friendly manufacturing technique for ZnO-NPs utilizing *Teucrium polium* extract (TP-ZnO-NPs) and assesses their anticancer efficacy on HGC-27 GC cells. Zinc acetate dihydrate was utilized for the development of nanoparticles. The nanoparticles revealed considerable antiproliferative and anticancer properties. Furthermore, quantitative real-time polymerase chain reaction (qRT-PCR) analysis revealed modified expression of genes associated with apoptosis and epithelial-mesenchymal transition (EMT) markers. TP-ZnO-NPs, produced by a sustainable and environmentally friendly method, have potential as a new therapeutic agent for the treatment of gastric cancer [120]. ZnO-NP have demonstrated the ability to suppress proliferation, migration, and invasion, while also causing apoptosis in both normal and cisplatin (DDP)-resistant GC cells. ZnO-NP significantly reduces the DDP IC_50_ in resistant cells and inhibits autophagy, an adaptive process associated with chemoresistance by lowering LC3II/LC3I and Beclin-1 levels while elevating p62 expression. ZnO-NP also mitigates DDP-induced autophagy and significantly suppresses tumor development *in vivo* [121].

#### 2.5.3 HCC (liver cancer)

ZnO-NPs demonstrate dose-dependent toxicity and antitumor efficacy, as demonstrated by elevated liver enzyme levels (alanine aminotransferase/aspartate aminotransferase (ALT/AST)), alterations in liver and spleen weight, and histological liver injury in rats. In human hepatocyte cell lines (HepG2 and HUH7), ZnO-NPs augmented the expression of pro-apoptotic genes (Bax and P53) while diminishing the expression of the anti-apoptotic gene Bcl-2, signifying the activation of apoptosis. These findings underscore the potential of ZnO-NPs as anticancer agents and indicate a dose range that yields therapeutic benefits with minimum damage [122].

### 2.6 Copper nanoparticles (CuNPs)

CuNPs have distinctive features that render them appropriate for several biological applications. The toxicity and underlying processes of CuNPs were assessed to investigate their effects on oxidative stress and apoptosis in the human CRC cell line SW480, a topic not previously studied. Following 24 h of exposure to CuNPs, oxidative stress levels were assessed by quantifying ROS generation. The activity of antioxidant enzymes was assessed by a colorimetric method. Apoptosis was examined using Hoechst 33258 staining, and the expression levels of the Bax, Bcl-2, and p53 genes were quantified by qRT-PCR. CuNPs reduced the viability of SW480 cells. A significant increase in ROS generation was detected at all examined doses (31, 68, and 100 μg/mL). Treatment with CuNPs resulted in the elevation of Bax and p53 expression and the downregulation of Bcl-2 expression. Apoptotic alterations were verified using Hoechst staining. CuNPs promote apoptosis and demonstrate potential anticancer properties [123]. CuNPs, functionalized with glutamic acid and conjugated with thiosemicarbazone (CuO@Glu-TSC NPs), were engineered as a prospective anticancer treatment for GC. The nanoparticles were analyzed for ligand binding, elemental composition, crystallinity, and physical characteristics, revealing a hydrodynamic size of 168 nm and a positive zeta potential. CuO@Glu-TSC nanoparticles significantly suppressed the growth of AGS gastric cancer cells while exhibiting reduced toxicity to normal HEK293 cells. Apoptosis induction was validated using flow cytometry, Hoechst staining, and elevated caspase-3 activity, highlighting the efficacy of CuO@Glu-TSC NPs as a targeted therapy for GC [124].

### 2.7 Titanium dioxide nanoparticles

Different types of TiO_2_ nanoparticles, amorphous, brookite, anatase, and rutile, each coated with PEG or bovine serum albumin (BSA) were observed to significantly diminish the viability and proliferation of MKN-45 GC cells in a dose- and time-dependent manner while promoting apoptosis. Notably, PEG-amorphous TiO_2_ enhanced cell invasion, but brookite BSA significantly reduced it. The structural and surface properties of TiO_2_ nanoparticles significantly influence cancer cell mortality and invasiveness, underscoring their therapeutic potential and the necessity for more investigation [125]. Zwitterionic chitooligosaccharide (COS)-modified ink-blue titanium dioxide nanoparticles (BTC NPs) were designed to enhance PTT by transforming TAMs from a pro-tumor M2-like form to a tumor-killing M1-like type, thus improving tumor suppression rates [126]. TiO_2_ NPs are widely used in cancer research and medical applications, including photodynamic therapy, sonodynamic therapy, imaging enhancement, radiation therapy, and drug delivery systems, although they may also cause harmful effects on some cells. TiO_2_ NPs are considered promising tools for developing advanced cancer therapeutics, and nanotechnology is expected to become increasingly important in future cancer treatment strategies [127].

The increasing global prevalence of liver cancer and the constraints of existing chemotherapy highlight the necessity for more effective therapies. TiO_2_ nanoparticles conjugated with thiosemicarbazone through glutamine functionalization (TiO_2_@Gln-TSC NPs) were synthesized. The resulting spherical particles measured 10–80 nm in size, exhibited a zeta potential of −57.8 mV, and had a hydrodynamic size of 127 nm. The nanoparticles demonstrated selective cytotoxicity, showing significantly higher toxicity to HepG2 liver cancer cells (IC_50_ = 75 μg/mL) than to normal HEK293 cells (IC_50_ = 210 μg/mL). Flow cytometry demonstrated an increase in apoptosis (from 2.8% to 27.3%) and a greater percentage of cells detained in the sub-G1 phase (34.1% compared to 8.4%). Hoechst staining validated nuclear damage, demonstrating that TiO_2_@Gln-TSC NPs successfully trigger apoptosis and show potential as a targeted therapy for liver cancer [128]. The effects of TiO_2_ nanoparticles on ER stress in liver cancer cells were investigated. TiO_2_ was found to significantly inhibit cancer cell growth, induce apoptosis, increase ROS levels, and cause G1 phase cell cycle arrest. Upregulation of ER stress markers PERK and ATF6, along with the pro-apoptotic protein Bax, was observed in a dose-dependent manner. These effects were specific to liver cancer cells, with no significant changes detected in normal liver cells. *In vivo*, tumor volume was reduced and expression levels of PERK, ATF6, and Bax were increased in tumor tissues following TiO_2_ administration, indicating that ER stress and apoptosis were induced through activation of the PERK/ATF6/Bax axis [129]. Table 1 shows the use of MNPs in the treatment of GI tumors [52,[54], [55], [56], [57],[59], [60],[63], [64], [65],^68^,[130], [131], [132], [133], [134], [135], [136], [137], [138]].

**Table 1:** A summary of the application of magnetic nanoparticles (MNPs) for gastrointestinal (GI) therapy.

Nanoparticle type	Cancer type/target	Nanoparticle size and Zeta potential	Delivery mechanism/targeting strategy	Therapeutic application	Diagnostic utility	Observed outcomes/efficacy	Toxicity/biocompatibility	Refs.
CUR-AgNPs (Curcumin-conjugated silver nanoparticles)	Colorectal cancer (Caco-2 cells)	Size: ∼5–10 nm (dominant smaller fraction); Zeta potential: Negative (AgNPs), becomes less negative upon CUR binding	Encapsulated in CHT/CS hydrogels; pH-responsive swelling (optimal in SIF); Passive diffusion and EPR effect assist in targeting	Photodynamic therapy (PDT); Antibacterial action	MEF (Metal-enhanced fluorescence) of CUR enables imaging; theranostic agent	High singlet oxygen generation (Φ1O2 increased from 0.01 to 0.14); Effective phototoxicity (CC50 = 91.5 μg/mL)	Non-toxic to healthy fibroblasts (>80% viability); Biocompatible hydrogels; [Hmim][HSO_4_] removed post-processing	[52]
AgNPs/Propolis-based APCPE biosensor	Hepatocellular carcinoma (HCC); liver cancer cell lines (Huh7, HepG2); miRNA-let 7a detection	Size: ∼35 nm (AgNPs from TEM)	AgNPs + propolis adsorbed on carbon paste electrode (CPE); DNA probe immobilization for miRNA-let 7a via propolis	–	Electrochemical impedance spectroscopy (EIS); Highly selective miRNA-let 7a detection with LOD of 10^−3^ fM	High sensitivity and selectivity; Differentiated HCC patients and healthy samples; Linear range: 10^−3^ fM to 1 μM; *R*^2^ = 0.993	Biocompatible sensor components (AgNPs, propolis, carbon paste); No interference from mismatched miRNAs; real sample validated	[54]
AgNPs functionalized with cDNA (cDNA@AgNPs)	Liver cancer (highly upregulated in liver cancer (HULC))	Size: ∼8 nm (80% of AgNPs)	Target-induced Y-shape structure recruits AgNPs to lipid bilayer (LB) modified Au electrode via hybridization with HULC.	–	Electrochemical detection (LSV) of HULC with antifouling LB; Detection limit: 0.42 fM; High selectivity	Linear detection from 1 to 500 fM; *R*^2^ = 0.99; Signal dependent on Y-shape formation; Stable over 21 days; Recovery in serum: 95.6%–104.6%	Excellent antifouling from lipid bilayer; High specificity in serum and biological fluids (PBS, FBS, HS); No interference from mismatched sequences	[55]
Biogenic AgNPs synthesized by *Bacillus safensis* TEN12	Liver cancer (HepG2 cell line); bacterial pathogens (*E. coli*, *S. aureus*)	Size: 22.77–45.98 nm (avg. ∼27.78 nm); spherical shape	Biogenic (intracellular synthesis via AgNO_3_ tolerance); no targeted delivery mechanism in study	Anticancer (against HepG2); Antibacterial (against *E. coli* and *S. aureus*)	–	Dose-dependent anticancer activity (cell viability dropped to 36.25% at 20 μg/mL); Effective antibacterial zones of inhibition up to ∼20 mm	No cytotoxic effect on normal HEK293 cells; selective cytotoxicity to HepG2; capped by functional proteins enhancing stability and biocompatibility	[56]
MLAgNPs (AgNPs synthesized using *Madhuca longifolia* leaf extract)	Liver cancer (HUH-7 cell line; DEN-induced hepatocarcinoma in Wistar rats)	Size: 5–20 nm (TEM), 20–65 nm (FESEM); spherical	Oral administration in rats; plant extract-based biogenic synthesis; capping by phytochemicals (e.g., flavonoids, glycosides)	Anticancer and hepatoprotective; reduced tumor nodules, improved liver histology	–	IC_50_ = 41.01 μg/mL in HUH-7 cells; ↓ liver tumor nodules, AFP, ALT/AST/ALP; ↑ antioxidants (SOD, CAT, GSH); restored ATPase activity; histologically improved liver structure	Non-toxic to normal tissue *in vivo*; improved liver function in rats; no systemic toxicity observed; phytochemical capping enhances biocompatibility	[57]
AgNPs synthesized via solution plasma method using Paramignya trimera extract	Gastric cancer (AGS cell line); antibacterial (*S. aureus*, *P. aeruginosa*)	TEM: 2–28 nm; Avg: ∼8–12 nm; spherical	Stabilized by phytochemicals in *P. trimera*; No targeted delivery; applied *in vitro*	Anticancer (AGS cell line); Antibacterial	–	IC_50_ = 30 μg/mL (AGS cells); ↓ cell viability to ∼1% at 50 μg/mL; Effective against Gram(+) and Gram(−) bacteria with inhibition zones up to 31 mm	Biocompatible due to plant capping agents; high colloidal stability up to 15 months; safe under tested *in vitro* conditions	[59]
Phytosynthesized AgNPs using Artemisia turcomanica	Gastric cancer (AGS cell line); also tested on normal L-929 fibroblast cells	Size: 20–60 nm (avg. 21.22 nm); spherical (TEM/SEM)	Biosynthesis using plant extract (green synthesis); no active targeting	Anticancer (AGS cells); apoptosis induction	–	IC_50_ = 4.88 μg/mL (AGS); ↑ apoptosis (38.94%), ↑ BAX (10.58 × ), ↓ Bcl2; Stronger than commercial AgNPs	IC_50_ = 14.56 μg/mL (L-929 normal cells); limited toxicity at low doses; apoptosis > necrosis; biocompatible at therapeutic levels	[60]
Chemically synthesized AgNPs (sizes: 2.6 nm and 18 nm)	Pancreatic cancer (PANC-1); compared with normal pancreatic cells (hTERT-HPNE)	Size: 2.6 ± 0.8 nm and 18 ± 2.6 nm; Zeta potential: −31.1 mV (PANC-1), −28.9 mV (hTERT); PDI ∼0.21; spherical (TEM)	Non-targeted; uptake via caveolae and passive diffusion; tested *in vitro*	Anticancer (PANC-1); apoptosis, necroptosis, autophagy induction	–	IC_50_ (MTT): 2.6 nm = 1.67 μg/mL; 18 nm = 26.81 μg/mL; ↓ proliferation (BrdU): IC_50_ = 1.91 and 21.76 μg/mL; ↑ Bax, p53, LC3-II, RIP1/3, MLKL; Nec-1 reversed cytotoxicity in PANC-1	IC_50_ (hTERT): 3.74 μg/mL (2.6 nm), 58.46 μg/mL (18 nm); Less cytotoxic to non-cancerous cells; no effect of Nec-1 in hTERT; size-dependent cytotoxicity	[63]
UiO-66-NH_2_ MOF nanoparticles (positively charged variants) with streptavidin (SA)	miR-221 (biomarker relevant in cancers, such as breast, liver, and pancreatic)	Sizes: 85, 150, 300, 600 nm; Zeta potential: +49.8 to +55.4 mV (positive), +3.7 mV (neutral), −37 mV (negative)	Immobilized on QCM chip via PDNA hairpin probe and SA–biotin binding; EXPAR amplification used for sensitivity	–	Detection of miR-221 with ultrahigh sensitivity using QCM + EXPAR	LOD: 0.79 aM; LOQ: 2.4 aM (with EXPAR); linear range from 1 aM to 1 nM; High specificity for miR-221 over mismatches	Biocompatible; tested in Tris buffer and diluted bovine serum; good recovery (94.4%–107.1%) and low RSD (<10%)	[64]
Fe-TBP (nanoscale MOF constructed from Fe_3_O clusters and TBP ligand)	CT26 colorectal adenocarcinoma (hypoxic tumor model)	∼100 nm	Passive tumor targeting; catalyzing intracellular H_2_O_2_ to O_2_ for enhanced PDT; used in combination with α-PD-L1 for immunotherapy	Photodynamic therapy (PDT); enhancing immune checkpoint blockade (ICB)	–	Under hypoxia, IC_50_ = 3.10 μM vs > 50 μM for controls; regressed primary and distant tumors (>90%) via abscopal effects; activated T/B cell response	No systemic toxicity; well-tolerated *in vivo*; cleared from tumor in 10 days; no dark toxicity	[65]
Fe-TCPP MOF (metal-organic framework with TCPP ligand and Fe^3+^)	Colorectal cancer (CRC), H_2_S-rich microenvironment	∼250 nm length, ∼100 nm width; Hydrodynamic size ∼300 nm in physiological media; PDI: 0.136–0.238	H_2_S-triggered degradation releases TCPP for fluorescence imaging and singlet oxygen (^1O_2_) generation; passive accumulation in tumors; tumor-selective activation	Photodynamic therapy (PDT)	Fluorescence switch "off-on" in CRC via H_2_S activation; targeted tumor imaging	*In vitro*: 60% killing at 10 μg/mL (laser); *In vivo*: significant tumor inhibition; stronger than TCPP control; high selectivity in CRC over normal cells	Excellent biocompatibility; no toxicity in normal cells/organs; stable in various media before activation; negligible side effects *in vivo*	[68]
Magnetic gold nanoparticles (Fe_3_O_4_@Au core with PEG and amine groups)	Colorectal cancer (LoVo and HCT116 cells); Target gene: Bag-1	∼95.4 ± 7.2 nm; Zeta potential: +35.0 ± 3.1 mV	Electrostatic binding to RNAi plasmid; internalization via nanoparticle-plasmid complex; some groups tested magnetic field-enhanced uptake	RNA interference (RNAi) targeting Bag-1 gene; inducing apoptosis and inhibits proliferation	GFP fluorescence reporter for transfection efficiency tracking	*In vitro*: ∼47.5% apoptosis in LoVo cells; ∼22% transfection efficiency; *In vivo*: significant tumor volume reduction in nano-plasmid groups vs. controls (*P* < 0.05); downregulation of Bag-1, C-myc, and β-catenin	Good biocompatibility; negligible cytotoxicity in normal colon cells (FHC); no significant tissue damage in major organs	[130]
TS265-loaded PEGylated gold nanoparticles (NanoTS265) and anti-EGFR-targeted version (TargetNanoTS265)	Colorectal cancer (HCT116); also tested on lung adenocarcinoma (A549, H1975)	AuNPs: 14 nm; NanoTS265: larger due to BSA (∼increased); TargetNanoTS265: including 2–3 anti-EGFR/particle; NanoPTX: 410 ± 19 nm	Passive targeting (EPR) for NanoTS265; Active targeting via anti-EGFR mAb for TargetNanoTS265	Chemotherapy using TS265 (novel drug); Paclitaxel (for comparison)	–	*In vitro*: HCT116 cell viability ↓ by 71.1% (NanoTS265) and 69.2% (TargetNanoTS265) vs. 48.7% (free TS265); 2.7-fold selectivity in co-culture; *In vivo*: tumor suppression 93% (TargetNanoTS265) vs. 69% (free TS265); gold accumulation confirmed in tumor	Excellent safety: minimal liver/spleen/kidney accumulation; no RES activation or hepatic toxicity; negligible gold in brain; selective cytotoxicity with minimal effect on fibroblasts	[132]
Bif@PAu-NPs (Bifidobacterium-coated polydopamine-gold nanoparticles)	Colorectal cancer (CT-26); also tested in A549 and 4T1 cells	Au-NPs: 33.4 ± 2.8 nm → PAu-NPs: 185.5 ± 2.1 nm; Zeta potential: −36.9 mV → −40.6 mV	Active targeting via hypoxia-seeking Bifidobacterium; EPR effect; laser-triggered release; immune activation via GM-CSF	Photothermal therapy (PTT) with near-infrared (808 nm) + immunotherapy (GM-CSF to activate DCs and T-cells)	Micro-PET/CT, fluorescence imaging (ICG), ICP-MS tracking, flow cytometry for T-cell and DC response	Tumor inhibition: up to 98.32% with PTT + GM-CSF; complete regression of primary tumors and no growth of secondary tumors; survival 100 % after 80 days	No significant toxicity: normal body weight, no hemolysis, no liver/kidney pathology; good hemocompatibility and immune activation without adverse effects	[133]
PD-L1-AuNP-DOX (Gold nanoparticles conjugated with anti-PD-L1 and Doxorubicin)	Colorectal cancer (CT-26 cells with PD-L1 overexpression)	Size: 23.1 nm (AuNP) → 62.0 nm (PD-L1-AuNP-DOX); Zeta potential: −29.6 mV → −11.1 mV	Active targeting via anti-PD-L1 antibody; receptor-mediated endocytosis; LA-PEG linker for DOX & antibody conjugation	Chemo-photothermal therapy (DOX + NIR-induced PTT)	Fluorescence microscopy (AlexaFluor488, DOX), CLSM, flow cytometry for uptake and apoptosis, ROS, and cell cycle analysis	IC_50_ = 0.25 μg/mL (lower than DOX or NT-AuNP-DOX); 60-fold increase in uptake over 0.5 h; Significant S/G2-M arrest, increased ROS, and apoptosis; Cell survival reduced to 10.5% with NIR	Excellent biocompatibility *in vitro*; Minimal toxicity from AuNP or antibody alone; ROS detox enzymes downregulated only with DOX formulations; No toxicity in absence of DOX or laser	[134]
GNP–F19 (gold nanoparticles functionalized with C-PEG-2S and anti-F19 mAb)	Pancreatic cancer (tumor stroma via FAP-α targeting)	Core: ∼15 nm → Hydrodynamic: ∼25 nm (after PEGylation); Antibody-conjugated particles: up to ∼100 nm (aggregates)	Passive + active targeting: PEG-dithiol coating for stability; F19 mAb targets FAP-α in tumor stroma; minimal aggregation	–	Darkfield microscopy (560 nm), scattering-based imaging of labeled tissues, SEC fraction analysis	Strong, specific labeling of pancreatic cancer stroma; minimal labeling of healthy tissue or controls; stable conjugates with long shelf life; 4–6 antibodies per AuNP; minimal aggregation in selected SEC fractions	High stability; no aggregation under protein-rich conditions; suitable for biological staining; long-term storage at 4 °C retains activity; biocompatibility presumed good, but no *in vivo* toxicity assay reported	[135]
Au@Tat-R-EK (Ultrasmall gold nanoparticles with cathepsin B-responsive peptide coating)	Liver cancer (LM3 orthotopic tumor; responsive to tumor microenvironment with cathepsin B overexpression)	Core size: ∼3.5 nm (dry); Hydrodynamic: ∼3.9 nm (bare), ∼4.9 nm (peptide coated); Zeta potential: negative → near-neutral (zwitterionic peptide) → positive (after cathepsin B activation)	Cathepsin B-responsive surface ligand (GFLG linker); exposes cell-penetrating Tat peptide in tumor; enhanced uptake & radiosensitization	Radiotherapy enhancement (X-ray, 6 Gy)	Indirectly via imaging of γ-H2AX, ROS, apoptosis; biodistribution tracked via ICP-MS	*In vitro*: ROS ↑, apoptosis ↑, DNA damage ↑; tumor volume reduced by 90% with Au@Tat-R-EK + X-ray; γ-H2AX foci ↑ to 27.8 (vs. 7.9 X-ray only); Survival ↑; tumor accumulation ↑3.6 × vs. control	Excellent *in vivo* biocompatibility: No change in IL-6, TNF-α, AST, ALT, BUN, CRE; No histopathological organ damage; ∼62% excretion in 48 h; Rapid renal clearance due to <5.5 nm size	[136]
siPLK1-StAv-SPIONs (Superparamagnetic iron oxide nanoparticles loaded with siRNA against PLK1)	Pancreatic ductal adenocarcinoma (PDAC) – orthotopic& genetically engineered mouse models	–	Dual strategy: Tumor-targeting peptide (EPPT1) for PDAC specificity + Myristoylated polyarginine for membrane translocation & endosomal escape	Gene silencing therapy (siRNA against PLK1) to inhibit tumor proliferation	MRI used to track nanoparticle accumulation and delivery efficacy *in vivo*	PLK1 expression ↓; Tumor growth halted; Proliferation ↓; Apoptosis ↑; Accumulation confirmed in tumor via MRI; Effective in both orthotopic and genetically engineered models	No specific toxicity mentioned; presumed well-tolerated as repeated biweekly treatment was conducted with therapeutic effect; MRI confirms tumor-specific localization	[137]

Abbreviations: MGNPs: magnetic gold nanoparticles; AuNPs: gold nanoparticles; PEG: polyethylene glycol; PDA: polydopamine; DOX: doxorubicin; TS265: cobalt(II) complex TS265; Co-Ox-AuNPs: oxaliplatin-loaded, anti-DR5 antibody-conjugated gold nanoparticles; TargetNanoTS265: anti-EGFR antibody-conjugated gold nanoparticles delivering TS265; Bif@PAu-NPs: *Bifidobacterium infantis*-conjugated polydopamine-coated gold nanoparticles; PD-L1-AuNP-DOX: PD-L1 antibody and DOX-conjugated gold nanoparticles; GNP–F19: PEGylated gold nanoparticles conjugated to F19 antibody; Au@Tat-R-EK: ultrasmall gold nanoparticles coated with multifunctional peptide; siPLK1-StAv-SPIONs: siRNA-loaded streptavidin-conjugated superparamagnetic iron oxide nanoparticles; CHT/CS/CUR-AgNPs: curcumin-loaded chitosan/chondroitin-sulfate hydrogel with silver nanoparticles; AgNPs: silver nanoparticles; APCPE: AgNPs/propolis-modified carbon paste electrode; cDNA@AgNPs: cDNA-functionalized silver nanoparticles; LB: lipid bilayer; CRC: colorectal cancer; PDAC: pancreatic ductal adenocarcinoma; HCC: hepatocellular carcinoma; PD-L1: programmed death-ligand 1; EGFR: epidermal growth factor receptor; DR5: death receptor 5; FAP-α: fibroblast activation protein alpha; uMUC1: under-glycosylated mucin 1; PLK1: polo-like kinase 1; HULC: highly upregulated in liver cancer; PTT: photothermal therapy; PDT: photodynamic therapy; PET/CT: positron emission tomography/computed tomography; MRI: magnetic resonance imaging; MEF: metal-enhanced fluorescence; CTL: cytotoxic T lymphocyte; IC_50_: half-maximal inhibitory concentration; CC_50_: cytotoxic concentration for 50% of cells; CC_90_: cytotoxic concentration for 90% of cells; ALT: alanine aminotransferase; AST: aspartate aminotransferase; RES: reticuloendothelial system; ROS: reactive oxygen species; siRNA: small interfering RNA; FBS: fetal bovine serum; PBS: phosphate-buffered saline; SIF: simulated intestinal fluid; SGF: simulated gastric fluid.

**Fig. 10:**
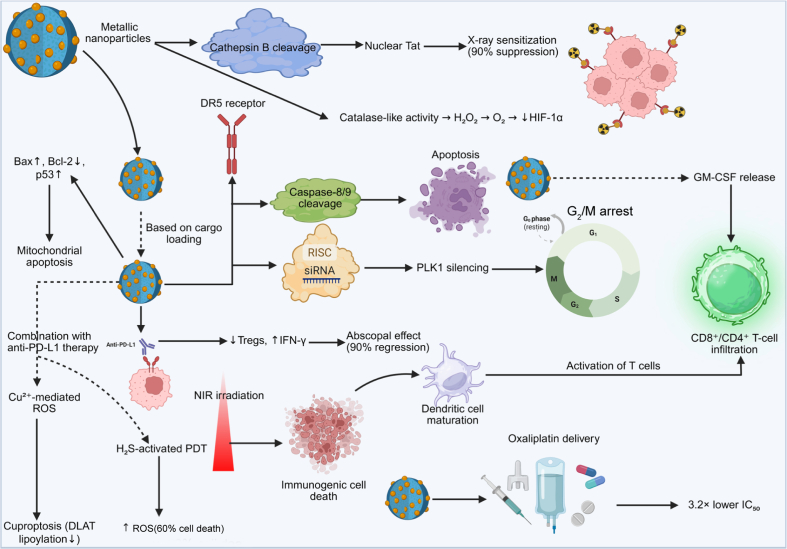
The molecular interactions of metallic nanoparticles in the treatment of gastrointestinal cancers. The cell death mechanisms including cuproptosis and apoptosis are affected. More focus should be on the regulation of other death pathways such as ferroptosis and autophagy. In addition, siRNA-polo-like kinase 1 (siPLK1) delivery can increase G2/M arrest. The release of granulocyte-macrophage colony-stimulating factor (GM-CSF) from nanoparticles can enhance the infiltration of cytotoxic T lymphocyte (CD^8+^)/T-helper cell (CD^4+^) T cells. These nanoparticles can stimulate immunogenic cell death to increase dendritic cell maturation in T-cell activation and enhance cancer immunotherapy. The reduction in Treg cells and increase in interferon gamma (IFN-γ) can promote an abscopal effect to mediate 90% regression. MNPs: metallic nanoparticles; nuclear Tat: nuclear trans-activator of transcription; DR5 receptor: death receptor 5; HIF-1α: hypoxia-inducible factor 1-alpha; Bax: Bcl-2-associated X protein; Bcl-2L1: B-cell lymphoma 2-like protein 1; p53: tumor protein p53; ROS: reactive oxygen species; anti-PD-L1: programmed death-ligand 1 antibody; PDT: photodynamic therapy; RISC: RNA-induced silencing complex; siRNA: small interfering RNA; PLK1: polo-like kinase 1; Tregs: regulatory T cells; IC_50_: half-maximal inhibitory concentration; DLAT: dihydrolipoamide acetyltransferase. Created with Biorender.com.

A major obstacle in developing metallic-based nanoparticles for GI tumor therapy is achieving a potent anticancer effect while ensuring selective biocompatibility across different subtypes of cancer subtypes and healthy tissues. Although numerous biogenic nanoparticles (ZnO, Cu, TiO_2_) exhibit significant tumor suppression *in vitro* and *in vivo* through mechanisms such as ROS generation, mitochondrial dysfunction, and the induction of apoptosis, maintaining consistent selectivity is challenging, particularly when moving from cell cultures to more complex biological systems. Moreover, the physicochemical characteristics of nanoparticles, including size, shape, and surface charge, significantly affect cellular absorption, distribution within the body, and treatment results; however, improving these factors while maintaining stability and scalability remains a constant challenge. A further concern pertains to the subtle effects of nanoparticle functionalization, such as PEGylation and ligand attachment, on immune responses and the modulation of the TME, where unintended consequences such as increased invasiveness (observed in certain TiO_2_ morphologies) highlights the necessity for precise design. Standardizing the synthesis process of nanoparticles and conducting comprehensive and long-term biosafety assessments across different GI cancer models are of utmost importance for guaranteeing clinical viability and reducing unexpected adverse effects. Fig. 10 demonstrates the regulation of molecular mechanisms by MNPs in cancer therapy.

## 3 Diagnosis of GI cancers

MNPs have emerged as pivotal tools in advancing diagnostic strategies for GI tumors, offering enhanced sensitivity, specificity, and multifunctionality compared to traditional agents. Their unique physicochemical properties, including high surface area-to-volume ratios, tunable surface chemistry, and the capability of being conjugated with diverse biomolecules, allow MNPs such as AuNPs, AgNPs, and IONPs to improve early tumor detection via modalities such as enhanced imaging contrast and targeted molecular recognition. In GI oncology, these nanoparticles have been integrated into platforms for MRI, CT, PA imaging, and SERS, enabling precise localization and characterization of tumors at early stages.

The application of nanotechnology in the diagnosis and treatment of CRC was thoroughly examined, with a particular emphasis on the use of nanoparticles for achieving precise detection and therapeutic interventions. Guanylyl cyclase C (GCC), a receptor exclusively expressed on CRC cells and lacking in extraintestinal organs, has been recognized as a viable molecular target, with nanoparticles coupled to the heat-stable enterotoxin (ST) suggested for precise targeting. Numerous nanostructures, including IONPs for MRI imaging and gold nanoshells for near-infrared thermal ablation, were delineated, emphasizing their potential to augment imaging sensitivity and therapeutic effectiveness. The methodologies for conjugating the ST to nanoparticles while maintaining the receptor-binding functionality were comprehensively elucidated. This highlighted the practical viability of employing these complexes for the targeted localization and subsequent destruction of tumors. The diagnostic capabilities of nanotechnology were examined for enhancements to both *in vitro* tests and *in vivo* imaging, whereas therapeutic applications emphasized targeted medication administration and the reduction of collateral harm to healthy tissues. It was determined that despite existing regulatory and practical challenges, nanotechnology had considerable potential to transform CRC management through novel approaches for detection, imaging, and treatment [138]. The efficacy of AuNP-based SERS for non-invasive detection of CRC has been also examined. Blood samples from patients and healthy volunteers were studied to reveal significant biochemical changes, including increased nucleic acids and decreased proteins and saccharides in cancer patients. SERS measurements were conducted, and diagnostic algorithms were formulated utilizing both empirical peak intensity ratios and multivariate statistical methods such as principal component analysis–linear discriminant analysis (PCA-LDA). The empirical technique exhibited moderate sensitivity and good specificity, but the PCA-LDA approach provided enhanced diagnostic accuracy, indicating significant promise for clinical use in CRC detection [139].

Peptides p.C, p.L, and p.14, derived from phage libraries, exhibit specific binding affinity to CRC. These biotinylated peptides were bound to 14 nm AuNPs through biotin-streptavidin interaction to improve cancer targeting for CRC diagnosis. Male Wistar rats were administered intravenous injections of citrate-AuNPs, PEG-AuNPs, or peptide-conjugated PEG-AuNPs (p.C-PEG-, p.L-PEG-, p.14-PEG-AuNPs) at a dose of 100 μg/kg. Behavioral, biochemical, oxidative injury and histological evaluations of the liver and colon were performed 14 and 84 days post-injection. Most AuNPs exhibited particle toxicity, except for p.L-PEG-AuNPs, which caused liver damage (*P* < 0.05) after 14 days, indicated by elevated liver weight, malondialdehyde levels, and histological changes. Consequently, with the exception of p.L-PEG-AuNPs, the peptide-linked AuNPs are mainly safe and biocompatible for *in vivo* use [140]. Moreover, a single-step aqueous synthesis technique was established to generate UiO-66-NH_2_(Hf) nanoparticles, a hafnium-derived MOF that acts as a radiosensitizer to improve the effectiveness of radiotherapy. These nanoparticles, with an approximate size of 95 nm and a hydrodynamic diameter around 128 nm, demonstrated remarkable physiological stability and a significant hafnium content of 44.1 wt%, thus ensuring effective X-ray absorption. Characterization (PXRD, BET, TEM, SEM) validated crystallinity, porosity, and an extensive surface area. In *in vitro* experiments, UiO-66-NH_2_(Hf) demonstrated low cytotoxicity by itself. However, it remarkably enhanced radiotherapy results against KYSE 150 EC cells by elevating DNA damage (γH2AX staining), intracellular ROS, and apoptosis (Bax upregulation, Bcl-2 downregulation). Clonogenic and wound healing tests verified decreased growth and movement. *In vivo*, intratumoral injection accompanied by 8 Gy X-ray irradiation resulted in significant suppression of tumor growth, with one instance reaching complete tumor regression. CT imaging confirmed increased X-ray accumulation at tumor locations, thereby validating its dual role in diagnosis and therapy. This straightforward, scalable synthesis and significant radiosensitization underscore UiO-66-NH_2_(Hf) as a potential, clinically applicable agent for radiotherapy [141]. MNPs play a crucial role in cancer imaging. Beyond that, they can also be integrated into theranostic platforms, which combine diagnostic and therapeutic functions seamlessly. IONPs are significantly utilized as contrast agents in MRI due to their superparamagnetic properties [142]. SnS nanorods demonstrating near-infrared photoelectric conversion properties were developed via a simple hydrothermal technique. AuNPs were subsequently self-assembled onto the surface of SnS nanorods, leading to the design of SnS/AuNPs nanocomposites. The integration of AuNPs significantly improved the photocurrent responsiveness of the SnS nanorods under 808 nm near-infrared light exposure. A near-infrared photoelectrochemical immunosensor was created utilizing SnS/AuNPs nanocomposites for the identification of the gastric cancer biomarker CA72-4. Experimental settings were meticulously refined to improve the immunosensor's efficacy in detecting CA72-4. A linear correlation was noted between the variation in photocurrent of the immunosensor (pre- and post-interaction with CA72-4) and the logarithm of CA72-4 concentrations spanning from 0.01 to 50 U/mL. The detection limit was established at 0.008 U/mL. The immunosensor's practical use was confirmed by the effective detection of CA72-4 in human serum samples [143].

## 4 Limitations and gaps: Perspectives for the future studies

Although MNPs hold great promise for transforming GI cancer theranostics, there are still notable gaps in converting preclinical achievements into reliable clinical results. A significant constraint exists due to the absence of standardized methods for synthesizing and functionalizing nanoparticles, thus resulting in inconsistencies in physicochemical characteristics such as size, surface charge, and ligand density. These discrepancies inhibit reproducibility among studies and make it difficult to compare efficacy and safety profiles. For example, although AuNPs are highly recognized for their biocompatibility, studies focusing on green-synthesized AuNPs derived from plant extracts frequently report inconsistent outcomes when compared to chemically synthesized counterparts, leading to concerns about scalability and consistency between batches. In a similar vein, AgNPs show strong anticancer properties but face issues with off-target toxicity, especially in healthy tissues that have high metabolic rates. To tackle these gaps, it is essential to develop modular synthesis platforms that separate nanoparticle core synthesis from surface modifications, allowing for accurate control over targeting ligands and therapeutic payloads. Moreover, the existing regulatory frameworks must be adjusted to accommodate the evolving characteristics of MNPs, including their potential to experience surface corona changes in biological fluids, which could affect their designed functionality. Another less-explored area is the relationship between MNPs and the GI microbiome, an intricate ecosystem that affects immune modulation, metabolic balance, and drug metabolism. Although MNPs such as IONPs have shown potential in magnetic targeting and enhancement of MRI contrast, the mechanism by which they disrupt microbial communities through oxidative stress or antimicrobial actions remains poorly understood. For instance, the wide-ranging antimicrobial effects of AgNPs might worsen gut dysbiosis. This, in turn, has the potential to impede the efficacy of immunotherapy treatments or prolong the recovery period for patients. Future studies should focus on creating microbiome-resilient MNPs, like those covered with pH- or enzyme-responsive polymers that restrict interaction with beneficial bacteria. Moreover, utilizing the microbiome as a therapeutic partner by means of probiotic-coated MNPs or microbiome-derived EVs infused with anticancer drugs might pave the way for novel synergistic treatment options. This strategy would correspond with new findings indicating that gut microbiota composition affects the effectiveness of immunotherapies such as checkpoint inhibitors, implying that maintaining microbial diversity could improve the therapeutic range of MNP-based treatments.

Combining MNPs with innovative technologies such as gene editing, artificial intelligence (AI), and closed-loop therapeutic systems presents a significant unexplored potential. AuNPs can affect the function of tumor suppressor genes (such as p53) by modulating oxidative stress, yet there are limited comprehensive studies connecting nanoparticle design features to particular genetic or signaling results. For example, MNPs carrying CRISPR-Cas9 might facilitate accurate modification of oncogenic drivers such as KRAS in PC or epigenetic regulators including EZH2 in CRC. Simultaneously, AI-based predictive modeling may enhance nanoparticle formulations by modeling their biodistribution, toxicity, and interactions with tumor-specific microenvironments. Closed-loop therapeutic systems incorporating real-time imaging (such as AuNP-enhanced photoacoustic imaging) and adaptive drug release, which are activated by biomarker thresholds identified through wearable sensors, would promote the personalization of treatment. These advancements would tackle the diversity of GI cancers and reduce dependence on standardized treatment strategies, finally bridging the gap between the aspirations of precision medicine and clinical practice.

## 5 Conclusion and perspectives

MNPs (AuNPs, AgNPs, IONPs, MOFs) have emerged as a groundbreaking tool in the identification and treatment of GI tumors, offering innovative solutions to persistent challenges in cancer care. The unique physicochemical properties of AuNPs, such as their high surface area-to-volume ratio, customizable surface chemistry, and biological interactions, have enabled significant advancements in targeted drug delivery, photothermal ablation, and radiosensitization for different GI cancers, including CRC, PC, and GC. The ability of AuNPs to convert light energy into heat, aiding in localized hyperthermia to eliminate cancer cells, is extremely important. Similarly, AgNPs have shown promise in enhancing the effectiveness of chemotherapy and PDT through the generation of ROS. IONPs have demonstrated significant potential in magnetic drug targeting, enhancing MRI contrast, and in synergistic chemo-photothermal therapy. The development of MOFs has expanded the applications of MNPs, showing potential in targeted drug delivery, multimodal imaging, and combination therapies, such as PDT and immunotherapy, for CRC and PC. These accomplishments highlight the versatility of MNPs in addressing various biological mechanisms linked to GI cancers, such as tumor hypoxia, drug resistance, and immune evasion. The ability of AuNPs to enhance drug retention in tumors through the EPR effect, coupled with the use of IONPs for targeted magnetic drug delivery, demonstrates the innovative approaches being explored. The combination of AuNPs with antibodies for directed action and the employment of AgNPs to induce apoptosis through mitochondrial disruption illustrates the various methods by which MNPs can tackle cancer. Despite these significant advancements, the clinical application of MNPs faces various limitations. Off-target impacts and long-term toxicity are major issues, as the small size and reactivity of nanoparticles can lead to unintended accumulation in healthy tissues, which may cause harmful effects. The scalability and reproducibility of nanoparticle manufacturing pose significant challenges, particularly for complex structures such as MOFs. Precise control over the size, shape, and surface alterations of nanoparticles is crucial for achieving the best therapeutic outcomes; however, current manufacturing techniques can show inadequate reliability. The long-term biological impacts of MNPs are not well comprehended, necessitating thorough investigation to assess potential risks, such as chronic inflammation or tissue harm.

Addressing these challenges requires a multidisciplinary approach, integrating materials science, biology, and clinical medicine to improve nanoparticle design, sharpen targeting accuracy, and minimize side effects. The development of uniform synthesis procedures and quality assurance protocols is vital for guaranteeing the reliability and safety of therapies utilizing MNPs. An in-depth comprehension of the prolonged biological interactions of these nanoparticles is crucial to reduce potential risks. The prospects for theranostics utilizing MNPs in GI cancer treatment are promising but demand-focused research. Future efforts should prioritize the development of biocompatible and biodegradable MNPs to reduce toxicity and enhance safety features. Integrating MNPs with advanced technologies, such as immunotherapy and gene editing, could provide combined advantages and overcome resistance mechanisms. Meanwhile, the combination of AuNPs with immune checkpoint inhibitors could enhance antitumor immunity, while the use of AgNPs for delivering gene-editing tools may aim at specific cancer mutations. Additionally, personalized medicine approaches that employ patient-specific biomarkers and AI-driven diagnostics may enhance MNP treatments for individual patients, increasing therapeutic efficacy and reducing side effects. By surmounting current limitations and utilizing innovative strategies, MNPs may significantly reduce the global challenge of GI cancers, offering new hope for improved patient results and survival rates. The continuous exploration of new uses and the improvement of existing technology will be crucial in realizing the full potential of MNPs in fighting GI cancers.

Despite progress in comprehending cancer biology and formulating novel therapeutics, conventional treatments such as chemotherapy, radiation, and surgery frequently prove inadequate due to toxicity, suboptimal drug delivery to tumors, and resistance. Nanotechnology provides viable answers to these constraints by facilitating the development of structures with distinctive characteristics, including small size, elevated surface-area-to-volume ratios, drug encapsulation, and biocompatibility, therefore improving targeting and minimizing side effects. These particles can administer numerous drugs concurrently, circumventing resistance mechanisms by simultaneously targeting many routes. Nonetheless, nanotechnology presents novel problems, including as toxicity arising from nanoparticle size, shape, charge, and composition, in addition to immune system identification and clearance. Strategies include PEGylation, use of natural lipids, and surface functionalization are implemented to mitigate these toxicities. Passive targeting employs the EPR effect along with the TME's attributes, such as acidity and enzyme overexpression, whereas active targeting entails the conjugation of nanoparticles with antibodies, aptamers, peptides, or ligands that promote receptor-mediated internalization into cancer cells. While PEG coatings can improve circulation duration, they may hinder tumor uptake, necessitating a compromise in nanoparticle design. Certain targeted nanoparticle systems have advanced to clinical trials, demonstrating potential in enhancing cancer therapy. The complete potential of cancer nanotechnology relies on interdisciplinary collaboration to address current obstacles in toxicity and targeted delivery, facilitating safer and more effective therapy [144].

A crucial yet neglected aspect in nanoparticle-driven GI cancer treatment is the alteration of intracellular signaling pathways that control cancer growth, survival, and resistance. Among these pathways, the phosphoinositide 3-kinase/protein kinase B/mammalian target of rapamycin (PI3K/AKT/mTOR) pathway has become a key regulator of cancer development and resistance to treatment. Dysregulation of this pathway leads to unchecked growth, blood vessel formation, and evasion of programmed cell death. The glucagon-like peptide-1 (GLP-1) receptor agonist liraglutide inhibits the PI3K/AKT/mTOR signaling pathway in CRC cells, which decreases proliferation, migration, and invasion, triggers apoptosis, and provides possibilities for combined targeted treatment [145]. MNPs, particularly gold and silver ones, have demonstrated interactions with this pathway by modifying phosphorylation cascades or changing upstream receptor signaling. Therefore, one of the prominent aspects for the research in the future can be understanding the impact of MNPs on the molecular pathways, especially PI3K/AKT. Additionally, it has been shown that nanoparticle-assisted drug delivery can enhance tumor sensitivity to pathway inhibitors or reduce mTOR activity, thereby providing synergistic benefits with current chemotherapeutic agents. Even with these progressions, a mechanistic grasp of how various metal cores, surface chemistries, and delivery methods affect PI3K/AKT/mTOR signaling is still inadequate and necessitates thorough molecular studies. Alongside affecting oncogenic pathways, MNPs might affect the expression and stability of tumor suppressor genes such as TP53, PTEN, and RB1, which are commonly modified in GI cancers. These tumor suppressor proteins control cell cycle checkpoints, DNA repair, and apoptosis, and their inactivation frequently correlates with a poor prognosis and resistance to treatment. However, the future research should provide a focus on the impact of nanoparticle design on gene expression in the different GI tumors. The studies should focus on delineating the gene-nanoparticle interaction framework through integrated omics methods, facilitating precise modulation of genetic pathways for improved therapeutic effectiveness. Moreover, bioinformatics evaluation and machine learning techniques have proven helpful in comprehending the biological development of GI tumors such as colon cancer [146]. Therefore, combining bioinformatics with MNPs may enhance the prospects for treating GI tumors.

Cancer therapies using nanoparticles excel at precisely targeting tumor locations; however, they frequently cause considerable dysbiosis in the GI microbiome due to collateral damage and oxidative stress. This disturbance undermines immune balance and could extend patient recovery time. The soft robot, designed with low friction and magnetic actuation offers a hopeful solution to tackle these side effects. Thanks to its terrain-adaptive movement and accurate payload delivery, the robot could be employed to transport microbiota-restoring substances such as prebiotics, probiotics, or postbiotics straight to dysbiotic areas. Additionally, its capacity to physically break apart biofilms and produce localized thermal effects provides a focused approach to eliminate harmful bacteria without systemic antibiotic administration, which in turn helps maintain commensal populations [147]. Although strategies using nanoparticles in GI therapy provide notable benefits such as targeted delivery, improved drug stability, and localized effects, a crucial limitation is often ignored: their ability to disturb the gut microbiome. The gut microbiome is essential for immune modulation, metabolic balance, and barrier function, all of which can be unintentionally influenced by stress from nanoparticles, immune activation, or antimicrobial effects. Recent studies have primarily concentrated on the treatment effectiveness and biodistribution while paying little attention to the microbiome changes after treatment. Future research should systematically analyze the short- and long-term impacts of different nanoparticle formulations on microbial diversity, composition, and functionality. This involves assessing the interactions of nanoparticles with mucosal surfaces, microbial biofilms, and immune-microbiome interactions. Developing nanoparticles that are beneficial to the microbiome or can modulate it might pave new paths for GI therapy, harmonizing therapeutic results with ecological conservation and host-microbe balance. Although MNPs possess significant potential in GI cancer theranostics, the literature presents contradictory results concerning their safety and efficacy, primarily due to differences in design, synthesis, and testing methods. Among MNPs, AgNPs have consistently demonstrated significant cytotoxic effects on various cancer cells, frequently via mechanisms such as oxidative stress induction, mitochondrial impairment, and DNA damage. Nonetheless, this potency presents a double-edged sword; AgNPs have also demonstrated greater toxicity to healthy cells than AuNPs and IONPs. These discrepancies can be attributed to variations in particle size, shape, surface charge, and the existence or nonexistence of biocompatible coatings. Additionally, even with the same core material, research utilizing green synthesis approaches frequently presents varying results compared to those utilizing chemical or physical synthesis methods, emphasizing the influence of nanoparticle formulation on biological activity and therapeutic range. Concerns about the reproducibility of findings across various studies have arisen due to the impact of nanoparticle size, surface modification, and fabrication methods on experimental results. Size is essential in pharmacokinetics, biodistribution, and cellular absorption; particles under 10 nm can undergo rapid renal elimination, whereas those exceeding 100 nm face removal by the reticuloendothelial system. Likewise, alterations to the surface such as PEGylation, antibody linking, or peptide functionalization significantly affect how MNPs interact with target tissues and the immune system. These factors can lead to the different outcomes even under the same experimental conditions. Moreover, varying applications of *in vitro* and *in vivo* models, discrepancies in cancer cell lines, and differences in dosing schedules have added to the difficulties in reaching clear conclusions about safety and effectiveness. In the absence of standardized procedures for nanoparticle development and biological assessment, the sector encounters a reproducibility challenge that hinders clinical application.

To assist in the informed choice of MNPs for upcoming research and clinical use, a comparative summary of frequently utilized nanoparticle categories in GI cancer studies is suggested. AuNPs are remarkable for their excellent biocompatibility, simple functionalization, and diverse functions in imaging and therapy, although they tend to be rather expensive. AgNPs, while being effective in causing cytotoxicity, pose issues associated with unexpected effects and safety over long periods. IONPs are particularly valuable in magnetic targeting and MRI imaging, providing dual capabilities; however, their therapeutic effect is typically less potent than that of AuNPs or AgNPs. MOFs exhibit significant potential for combination therapy and drug delivery due to their high porosity and customizable chemistry, but they are still in the early stages of development, facing unresolved challenges concerning *in vivo* stability and degradation.

## CRediT authorship contribution statement

**Xu Han:** Writing – review & editing, Conceptualization, Writing – original draft. **Ding Ding:** Writing – review & editing, Conceptualization, Writing – original draft. **Milad Ashrafizadeh:** Writing – review & editing, Writing – original draft. **Gautam Sethi:** Writing – review & editing, Conceptualization. **Ziwen Wang:** Writing – review & editing, Conceptualization. **Yuting Zhang:** Writing – review & editing, Conceptualization.

## Declaration of generative AI in scientific writing

During the preparation of this work the author(s) used AI tool known as ChatGPT in order to improve writing and grammar of this manuscript. After using this tool/service*,* the author(s) reviewed and edited the content as needed and take(s) full responsibility for the content of the published article.

## Declaration of competing interest

The authors declare that they have no known competing financial interests or personal relationships that could have appeared to influence the work reported in this paper.
